# Bonding and Reactivity of a Pair of Neutral and Cationic Heterobimetallic RuZn_2_ Complexes

**DOI:** 10.1021/acs.inorgchem.1c02072

**Published:** 2021-10-18

**Authors:** Fedor M. Miloserdov, Anne-Frédérique Pécharman, Lia Sotorrios, Nasir A. Rajabi, John P. Lowe, Stuart A. Macgregor, Mary F. Mahon, Michael K. Whittlesey

**Affiliations:** † Department of Chemistry, University of Bath, Bath BA2 7AY, U.K.; ‡ Institute of Chemical Sciences, School of Engineering and Physical Sciences, Heriot-Watt University, Edinburgh EH14 4AS, U.K.; § Laboratory of Organic Chemistry, Wageningen University, Stippeneng 4, Wageningen 6708, WE, The Netherlands

## Abstract

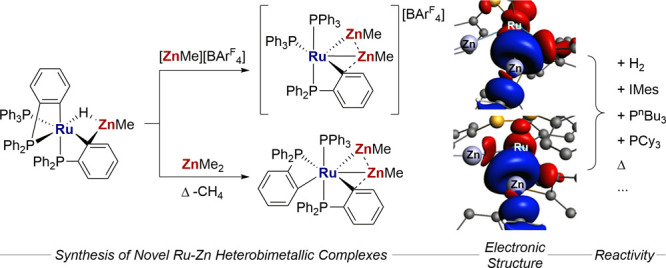

A combined experimental and computational study of the structure and reactivity of two [RuZn_2_Me_2_] complexes, neutral [Ru(PPh_3_)(Ph_2_PC_6_H_4_)_2_(ZnMe)_2_] (**2**) and cationic [Ru(PPh_3_)_2_(Ph_2_PC_6_H_4_)(ZnMe)_2_][BAr^F^_4_] ([BAr^F^_4_] = [B{3,5-(CF_3_)_2_C_6_H_3_}_4_]) (**3**), is presented. Structural and computational analyses indicate these complexes are best formulated as containing discrete ZnMe ligands in which direct Ru–Zn bonding is complemented by weaker Zn···Zn interactions. The latter are stronger in **2**, and both complexes exhibit an additional Zn···C_aryl_ interaction with a cyclometalated phosphine ligand, this being stronger in **3**. Both **2** and **3** show diverse reactivity under thermolysis and with Lewis bases (P^n^Bu_3_, PCy_3_, and IMes). With **3**, all three Lewis bases result in the loss of [ZnMe]^+^. In contrast, **2** undergoes PPh_3_ substitution with P^n^Bu_3_, but with IMes, loss of ZnMe_2_ occurs to form [Ru(PPh_3_)(C_6_H_4_PPh_2_)(C_6_H_4_PPhC_6_H_4_Zn(IMes))H] (**7**). The reaction of **3** with H_2_ affords the cationic trihydride complex [Ru(PPh_3_)_2_(ZnMe)_2_(H)_3_][BAr^F^_4_] (**12**). Computational analyses indicate that both **12** and **7** feature bridging hydrides that are biased toward Ru over Zn.

## Abstract 2

Experimental and computational studies of two [Ru-Zn_2_R_2_] heterobimetallic complexes, neutral [Ru(PPh_3_)(Ph_2_PC_6_H_4_)_2_(ZnMe)_2_] and cationic [Ru(PPh_3_)_2_(Ph_2_PC_6_H_4_)(ZnMe)_2_]^+^, reveal Ru−ZnR and Zn···Zn interactions suggesting that the complexes are best formulated as [Ru(ZnR)_2_] species. The Ru−Zn bonding is supplemented by peripheral Zn···C_aryl_ interactions to a cyclometalated phosphine ligand. Studies of the reactivity of the two complexes with respect to thermolysis, a range of Lewis bases (PR_3_ and NHC), and H_2_ reveal diverse behavior, with both Ru and Zn acting as the reaction center.

## Introduction

**Scheme 1:**
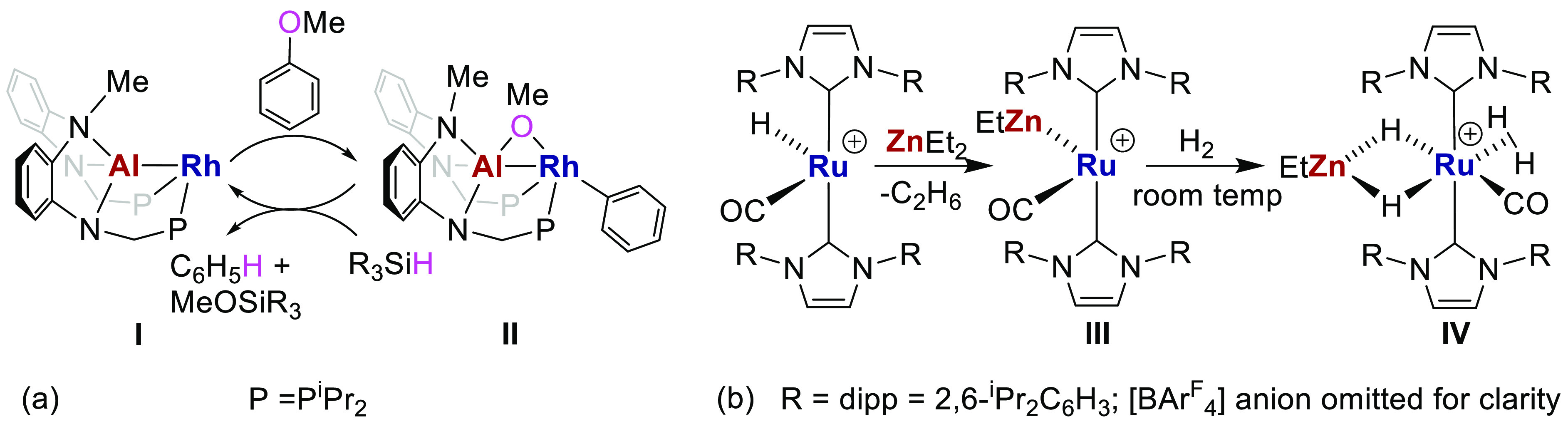
Examples of Cooperativity in Small Molecule Activation by TM–MGM Heterobimetallic Complexes

Heterobimetallic complexes comprised of a transition metal (TM) and a main group metal (MGM) are the focus of considerable interest^1−3^ because of the possibility that the disparate chemistry of the two partners could combine cooperatively to bring about the novel stoichiometric and/or catalytic activation of small molecules.^4−9^ In one recent example, shown in Scheme 1a, the challenging C–O activation of an anisole takes place across the Rh–Al bond of complex **I** to afford **II**, which upon addition of a silane, mediates catalytic C–O bond reduction.^10^ Complex **I** represents one class of heterobimetallic complexes in which the MGM forms part of a multidentate ligand on the TM center.^11^ Another class of complex is represented by **III** in Scheme 1b, in which the MGM is unsupported and unconstrained. In this particular case, both Ru and Zn centers are coordinatively unsaturated, and this “dual unsaturation” allows them to act cooperatively in the stoichiometric activation of H_2_ to give **IV**.^12^ We have interpreted Ru–Zn bonding within complex **III** and other related Ru–Zn complexes^13,14^ in terms of a donor–acceptor interaction between a Ru(0) metal center and Z-type Zn-based acceptor ligands.

**Scheme 2:**
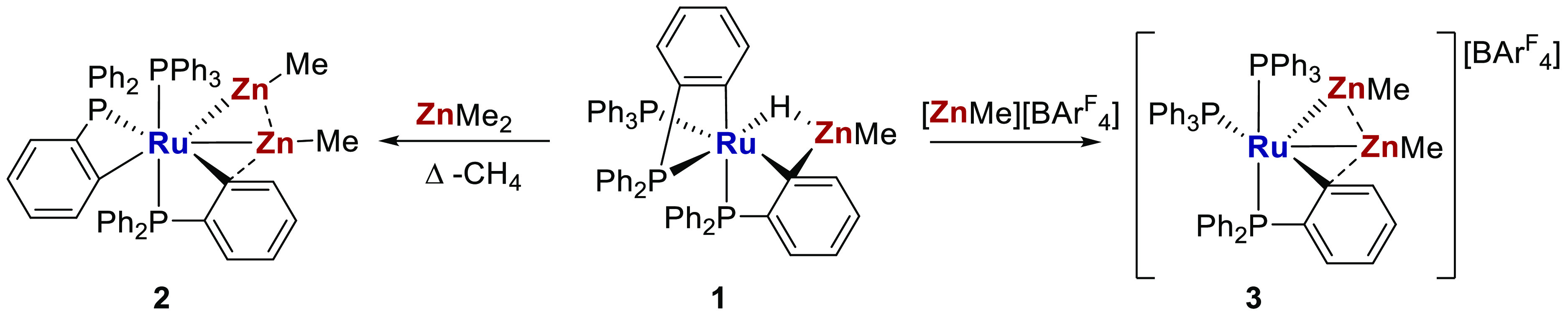
Syntheses of Neutral and Cationic [RuZn_2_Me_2_] Complexes **2** and **3** [BAr^F^_4_] = [B(3,5-(CF_3_)_2_C_6_H_3_)_4_]. The nature of the bonding between centers connected by dashed lines is investigated herein.^22^

Complex **III** is formed upon elimination of an alkane,^15−18^ an approach we have used to prepare other Ru and mono-Zn-containing products, including complex **1** in Scheme 2 that features bridging hydride and aryl ligands.^12−14,19−21^ Accordingly, the reaction of complex **1** with ZnMe_2_ resulted in further alkane elimination and formation of neutral [RuZn_2_Me_2_] complex **2**.^14^ Alternatively, reaction with a source of [ZnMe]^+^ induced C–H reductive coupling in **1** and formation of cationic [RuZn_2_Me_2_] complex **3**.^13^

Another strategy for the preparation of [TM-Zn_2_R_2_] species^23−25^ involves addition of Carmona’s Cp*Zn–ZnCp* dimer to low-valent precursors.^26,27^ On the basis of the isolobal nature of Cp*Zn and a hydrogen atom, the coordination of the TM center to an intact Zn–Zn bond can be considered to form an all-metal analogue of a TM(η^2^-H_2_) complex. Likewise, weakening of the Zn–Zn interaction to the point where it gives two ZnCp* ligands has been compared to the oxidative cleavage of the η^2^-H_2_ ligand to form two M–H bonds, although such Zn–Zn bond cleavage is proposed to proceed without any change in the formal oxidation state.^23,26^ In such cases, ZnCp* and related ZnR (R = alkyl or aryl) ligands have been formulated as monovalent one-electron donors.^26^ Computational studies have suggested that the extent to which the Zn–Zn interaction in Cp*Zn–ZnCp* is retained upon approach to a TM is dependent on the nature of the metal itself, the surrounding ancillary ligands, and the ZnR substituents.^26−28^

In this context, the availability of the closely related neutral and cationic [RuZn_2_R_2_] complexes, **2** and **3**, respectively, provides an opportunity to explore the analogy between {RZn-ZnR} and H_2_. Herein, we report computational and experimental studies to this end.

## Results and Discussion

### Structure and Bonding in **2** and **3**

**Figure 1:**
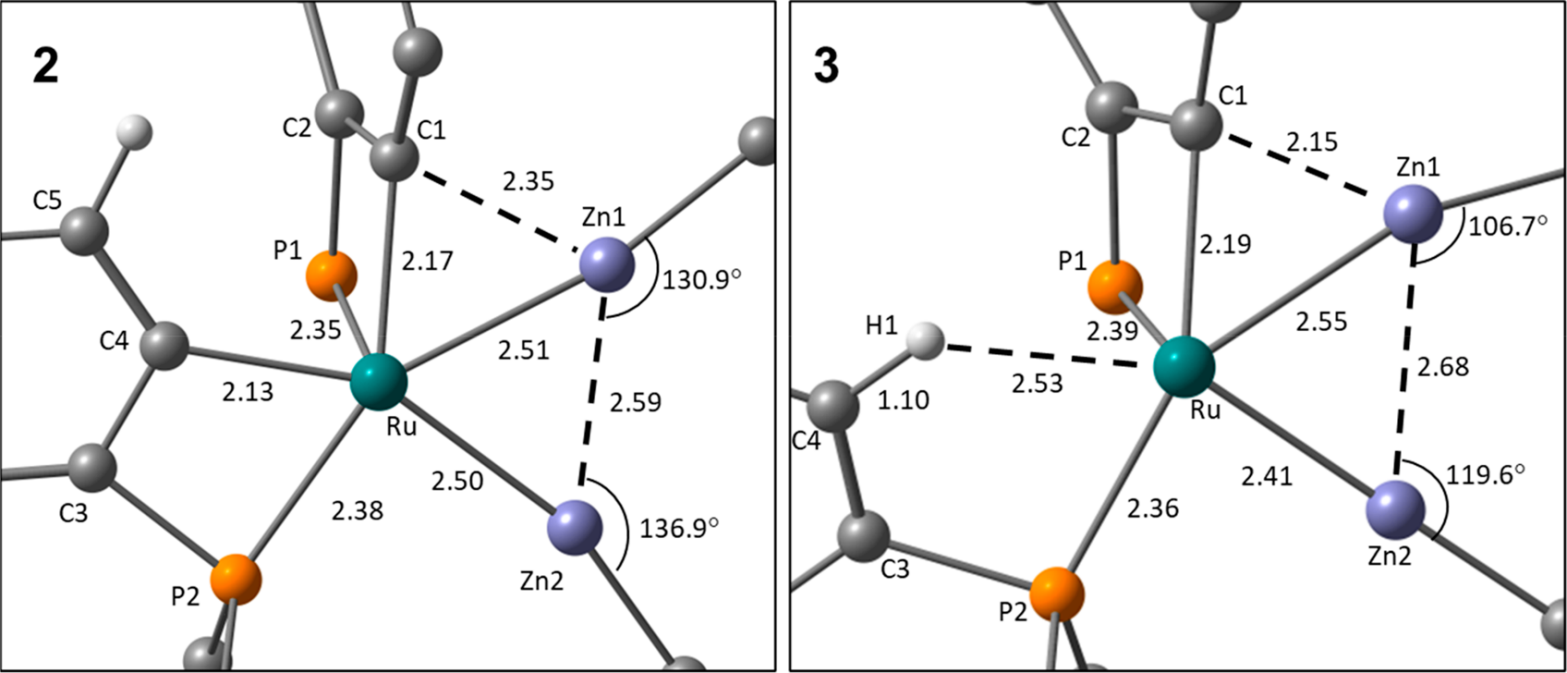
Geometries and labeling scheme used in the analyses of **2** and **3**, focusing on the central RuZn_2_ core. Selected distances are given in angstroms.

Figure 1 shows the geometries and labeling system used in the discussion of **2** and **3**. In general, good agreement was seen between the experimental and fully optimized structures; however, the Zn1···C1 distances were overestimated by 0.06–0.20 Å depending on the functional used (Supporting Information). Therefore, to analyze the observed geometries, we have taken the heavy atom (i.e., non-H) positions from the crystallographic studies and optimized the H atom positions with the BP86 functional. This approach also allows for a consistent treatment of the new hydride-containing structures that we describe below, where the H atom location is intrinsically less precise.

Both **2** and **3** feature triangular RuZn_2_ moieties with Ru–Zn distances that are shorter than Zn–Zn distances. The Ru–Zn distances are more symmetrical in **2** (2.50/2.51 Å) than in **3** (2.41/2.55 Å) and are all within the sum of the covalent radii (Ru, 1.46 Å; Zn, 1.22 Å),^29^ suggesting direct Ru–Zn bonds in all cases. Zn–Zn bonding in related systems has been discussed within the limits of the Zn–Zn distance in Zn_2_Cp*_2_ (2.31 Å), and the metallic radius of Zn (1.339 Å) and the Zn–Zn distances in **2** and **3** (2.59 and 2.68 Å, respectively) are at the upper end of this range.^26^ A [TMZn_2_R_2_] unit with an intact η^2^-RZn–ZnR moiety would also be characterized by near-linear Zn–Zn–R angles and M–Zn–R angles approaching 150° (the limit for an equilateral triangle).^26^ In **2**, the average Zn–Zn–R and Ru–Zn–Me angles are 133.9° and 165.8°, respectively, while in **3**, the average Zn–Zn–R angle is 113.2° and the Ru–Zn2–Me angle is 179.4°. These data indicate that **2** and **3** are best formulated as Ru(ZnMe)_2_ complexes, but that **2** is slightly displaced along the continuum toward a Ru(η^2^-RZn–ZnR) species.^26^ Note that in **3**, the Ru–Zn1–Me angle is smaller than expected at 161.3°, but in this case, the Me group is bent away from C1, suggesting that it is the short Zn1···C1 contact of 2.15 Å that drives this distortion. This is explored further in the electronic structure analyses below.

**Figure 2:**
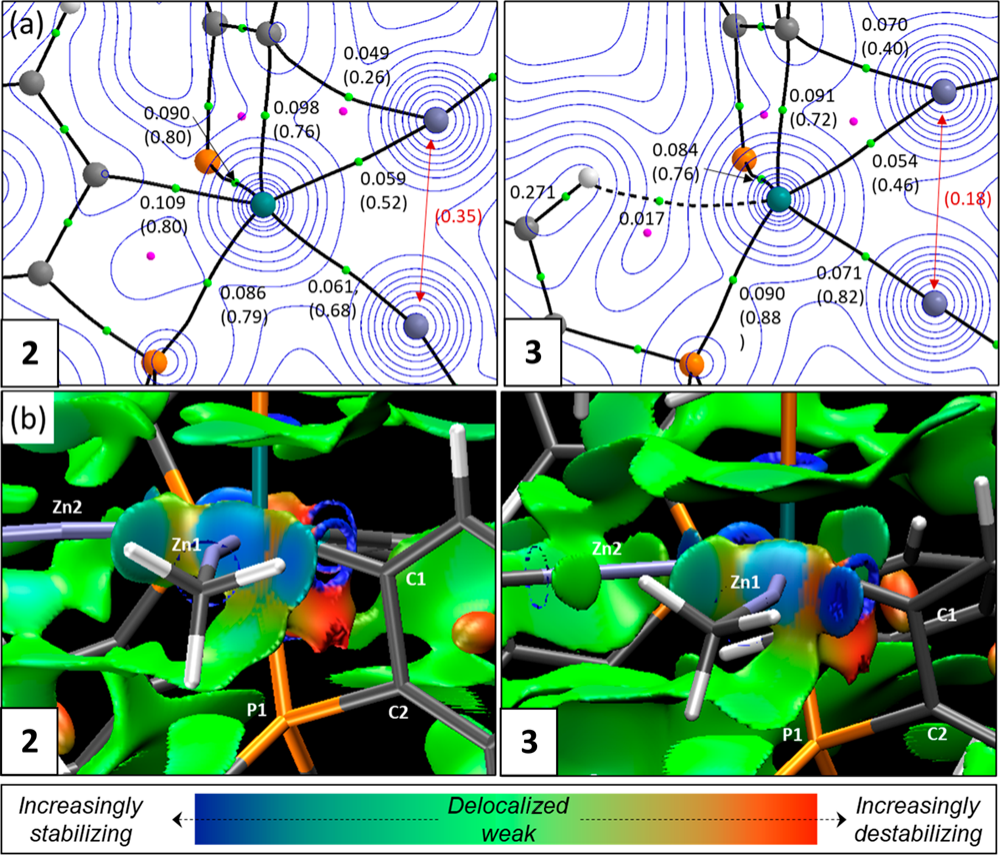
Electronic structure analysis of (left) **2** and (right) **3** focusing on key interactions around the {RuZn1Zn2} plane. (a) QTAIM molecular graphs with bond critical points (BCPs) in green and ring critical points (RCPs) in pink. Electron density, ρ(*r*), contour plots are shown in the {RuZn1Zn2} plane along with selected BCP (au) and delocalization indices in parentheses; delocalization indices between atoms not linked by a bond path are colored red. (b) Detail of the NCI plots viewed from above the {RuZn1Zn2} plane and looking down the Ru–Zn1 vector. Isosurfaces are generated for σ = 0.3 au and −0.07 < ρ < 0.07 au; a key showing the color scheme employed is also provided.

Figure 2a provides details of quantum theory of atoms in molecules (QTAIM) analyses of **2** and **3** with electron density contours plotted in the {RuZn1Zn2} plane. These are complemented by noncovalent interaction (NCI) plots shown in Figure 2b. For **2**, bond paths between Ru and both Zn centers are consistent with the presence of Ru–Zn bonds. The associated bond critical points (BCPs) show similar electron densities, ρ(*r*), of ∼0.06 au, and this relatively low value, coupled with positive values of the Laplacian and small negative total energy densities (Figures S39 and S40), is consistent with a donor–acceptor (i.e., Ru → Zn) interaction between two heavy atoms.^30,31^Figure 2a also shows the computed delocalization indices (DI) in parentheses. These reflect the degree of shared electron density between two atomic centers^32,33^ and proved more discriminating than ρ(*r*) for the Ru–Zn interactions. Thus, a larger DI of 0.68 is associated with the shorter Ru–Zn2 bond compared to a DI of 0.52 for the Ru–Zn1 bond. DIs can also be measured between atoms not linked by a bond path and can be useful for identifying interactions in areas of flat electron density.^34^ A Zn1···Zn2 DI of 0.35 suggests a weak Zn···Zn interaction is present. For Zn1, this is supplemented by interaction with C1 to which a curved bond path [2.35 Å; ρ(*r*) = 0.049; DI = 0.26] indicates a degree of bridging character for the cyclometalated aryl group, albeit biased toward Ru [2.17 Å; ρ(*r*) = 0.098 au; DI = 0.76]. These stabilizing interactions are confirmed by the NCI plot of **2** that displays turquoise and blue regions along the Zn1···Zn2 and Zn1···C1 vectors, respectively.

For **3**, variations in these different interactions are seen relative to **2** that reflect changes in the interatomic distances. Thus, the Ru–Zn2 interaction strengthens [2.41 Å; ρ(*r*) = 0.071; DI = 0.82] while Ru–Zn1 interaction weakens [2.55 Å, ρ(*r*) = 0.054; DI = 0.46]. The Zn1···Zn2 interaction also weakens significantly (2.68 Å; DI = 0.18); for Zn2, this is compensated by the stronger interaction with Ru, whereas for Zn1, the interaction with C1 strengthens [2.15 Å; ρ(*r*) = 0.070; DI = 0.40]. The stronger Zn1···C1 interaction is also apparent in the NCI plot where a sharper blue disk along the Zn1···C1 vector is seen, and this is also consistent with the bending of the Ru–Zn1–Me angle away from C1 as noted above. A similar Zn···C_aryl_ interaction has been noted before in a related Ru–Zn bimetallic complex [Zn···C, 2.282(2) Å^13^], while the longer Zn–C_aryl_ distances in the asymmetrically bridged [ZnPh]_2_ dimer average 2.40 Å.^35^

**Figure 3:**
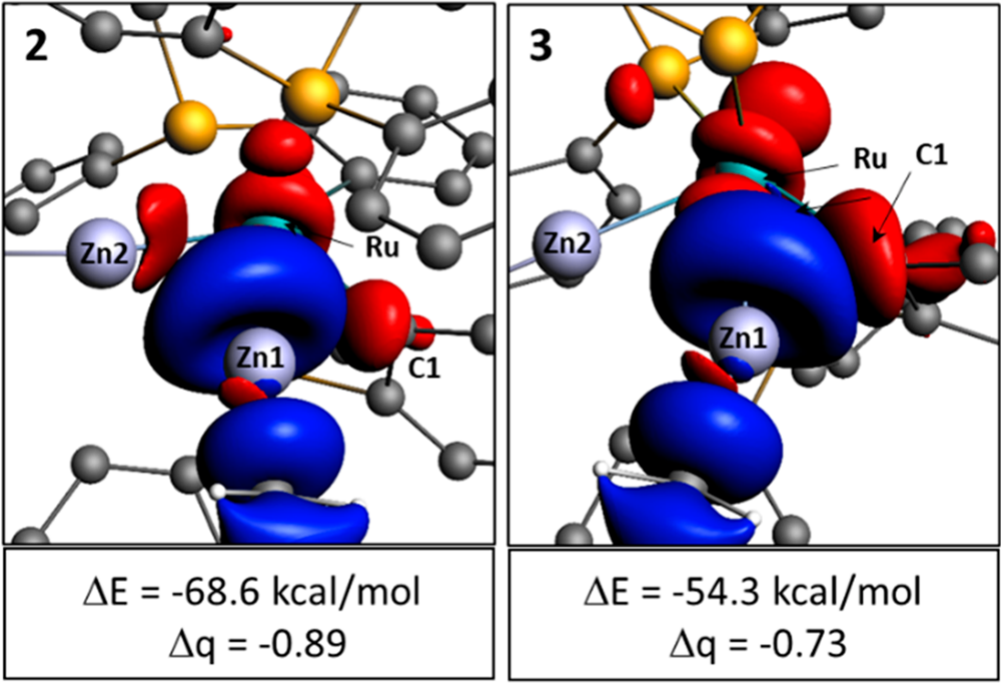
NOCV contour plots (isovalue of 0.0025 au) of the major deformation density channels in the interaction of the {Zn1Me}^+^ fragment with [Ru(PPh_3_)(PPh_2_C_6_H_4_)_2_(ZnMe)]^−^ in **2** and with [Ru(PPh_3_)_2_(PPh_2_C_6_H_4_)(ZnMe)] in **3**. Electron flow is shown from red to blue, and H atoms have been omitted for the sake of clarity.

A natural orbitals for chemical valence (NOCV) analysis confirmed the differences in the additional stabilizing interactions at the {Zn1Me}^+^ fragments in **2** and **3** (Figure 3). In each case, the key deformation density channel is dominated by donation from the *dsp* hybrid HOMO of the Ru-based fragment into the σ* LUMO of {ZnMe}^+^. For **2**, this also shows contributions from both Zn2 and C1, whereas for **3**, a larger component from C1 is apparent but no contribution from Zn2 is seen. Equivalent plots for the {Zn2Me}^+^ fragments are provided in Figures S46 and S47.

### Reactivity Studies

**Scheme 3:**
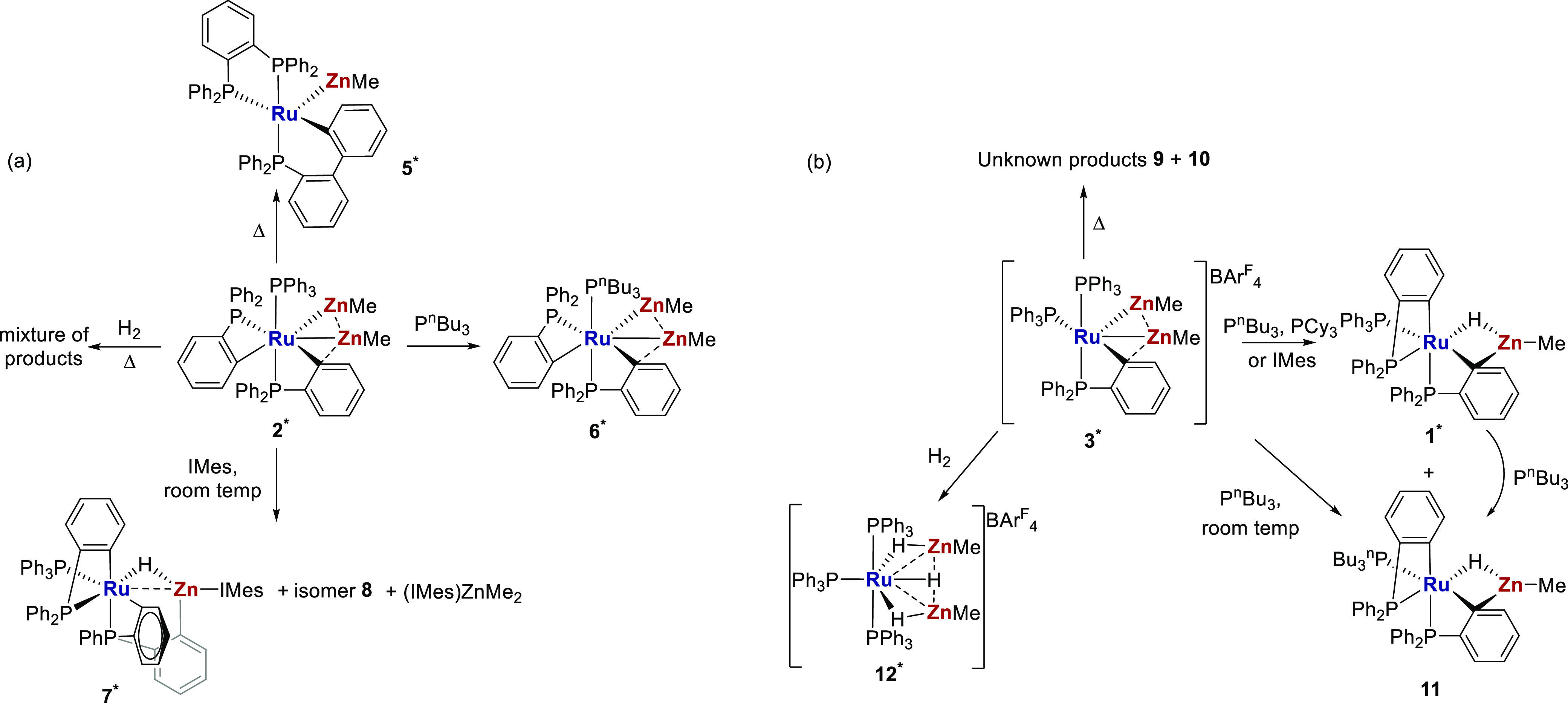
Reactions of (a) [Ru(PPh_3_)(C_6_H_4_PPh_2_)_2_(ZnMe)_2_] **2** and (b) [Ru(PPh_3_)_2_(C_6_H_4_PPh_2_)(ZnMe)_2_][BAr^F^_4_] **3** Structurally characterized complexes (reported here or previously in refs (13), (14), and (20)) are marked with asterisks.

Given that **2** and **3** result from the formal introduction of ZnMe and [ZnMe]^+^, respectively, into **1**, a series of reactivity studies were undertaken to probe the potential to reverse this process, through either thermolysis or reactions with Lewis bases. Such processes probe further the isolobality of ZnR and H, for example, the deprotonation of TM-hydrides. The reactions of **2** and **3** with H_2_ were also attempted, and the results are summarized in panels a and b of Scheme 3.

#### Thermal Stabilities of **2** and **3**

Heating **2** in toluene (80 °C, 2 days) resulted in the loss of ZnMe to give the previously reported [RuZnMe] complex, [Ru(dppbz)(PPh_2_(biphenyl′))(ZnMe)] [**5**; dppbz = 1,2-bis(diphenylphosphino)benzene; PPh_2_(biphenyl)′ = cyclometalated PPh_2_(biphenyl)], as the major product.^20^ Alongside formal elimination of ZnMe,^36^ the formation of **5** also requires C–H/P–C activation and C–C coupling steps to generate the dppbz and metalated Ph_2_P(biphenyl) ligand, although the exact sequence in which these steps take place remains unknown.^37^ For **3**, the result of heating proved to be much less clear. ^31^P NMR monitoring of a reaction mixture refluxed in benzene (2 days) or refluxed in toluene (2 h) revealed formation of an initial product **9** (characterized by two coupled doublet resonances at δ 78 and 48) that, upon further heating (2 days) in toluene, converted to a second product, **10**, which showed a similar pair of coupled signals at δ 53 and 46. Both compounds gave oils in all tested combinations of solvents,^38^ which, together with an absence of any diagnostic (i.e., non-aromatic) ^1^H NMR signals, makes their identities hard to determine.

#### Reactivity of **2** and **3** with Lewis Bases

**Figure 4:**
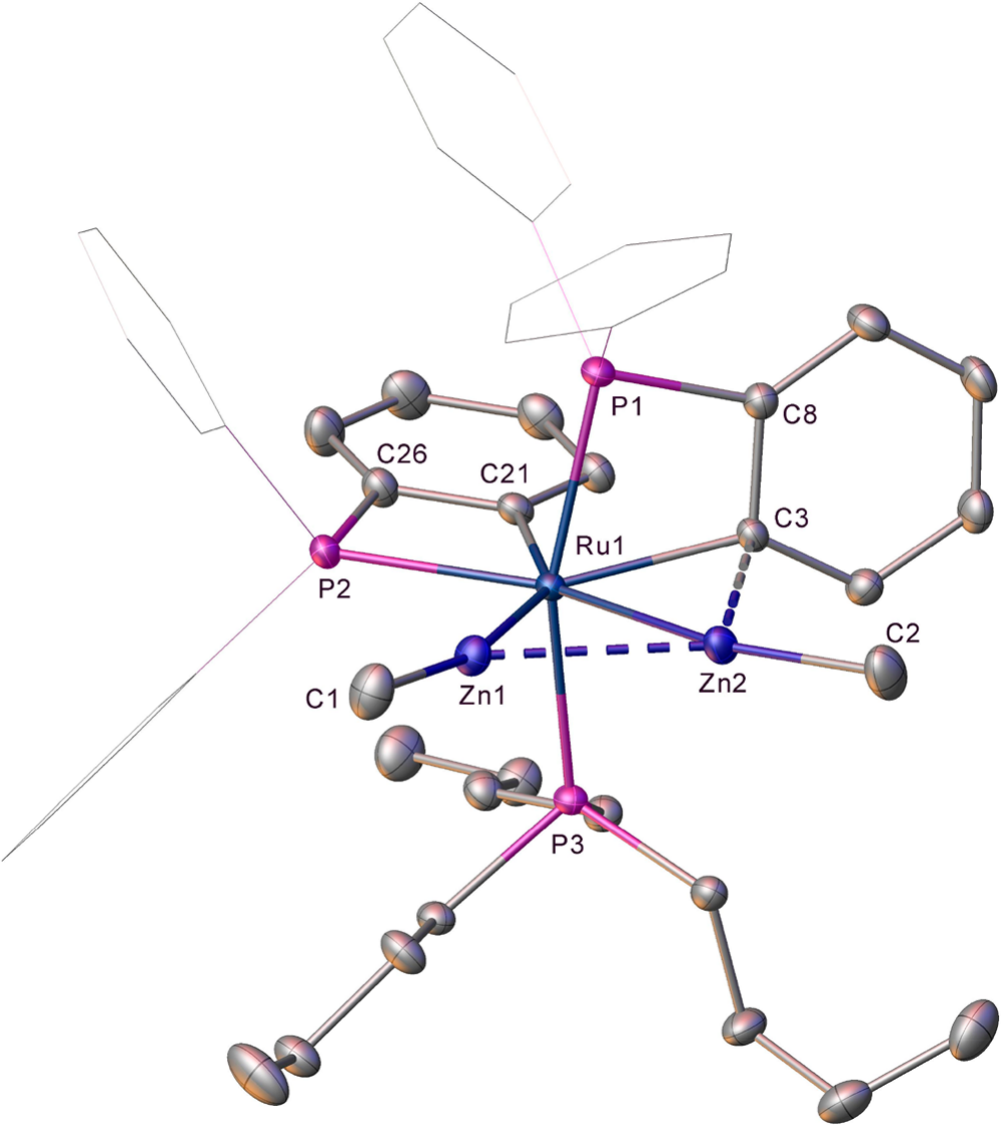
Molecular structure of **6**. Ellipsoids are represented at 30% probability. Hydrogen atoms and a minor disordered component have been omitted for the sake of clarity.

Rather than removing either of the ZnMe ligands, the reaction of **2** with P^n^Bu_3_ at room temperature led to substitution of the PPh_3_ ligand and formation of [Ru(P^n^Bu_3_)(C_6_H_4_PPh_2_)_2_(ZnMe)_2_] (**6**). X-ray characterization (Figure 4) revealed a structure that was broadly similar to that of **2** in terms of metrics (Table S2). The ease of phosphine substitution in **2** contrasts with the difficulties reported by Fischer in attempting to exchange phosphine ligands in multi-Zn-containing Ni species.^39^ At the same time, the lack of reaction between **2** and PCy_3_ indicates how sensitive these systems are to the choice of Lewis base. In contrast, **3** reacted with both P^n^Bu_3_ and PCy_3_, and in this case, this did incur the loss of [ZnMe]^+^ to give **1**, together with **11** in the case of P^n^Bu_3_.^40,41^ The findings fit with the previously observed complete conversion of **3** into **1** that is seen in THF.^13^ The fate of the eliminated [ZnMe]^+^ could not be established, but when the Lewis base is changed to the N-heterocyclic carbene IMes,^42^ trapping as the NHC adduct [(IMes)_2_ZnMe]^+^ was found,^43^ alongside formation of **1**.

A very different outcome was found when IMes was reacted with **2**. This afforded [Ru(PPh_3_)(C_6_H_4_PPh_2_)(C_6_H_4_PPhC_6_H_4_Zn(IMes))H] (**7**) through substitution of a Me group by IMes on Zn. The formally eliminated ZnMe_2_ was trapped as (IMes)ZnMe_2_ by the second equivalent of carbene necessary to bring about the full consumption of **2**.^44,45^

**Figure 5:**
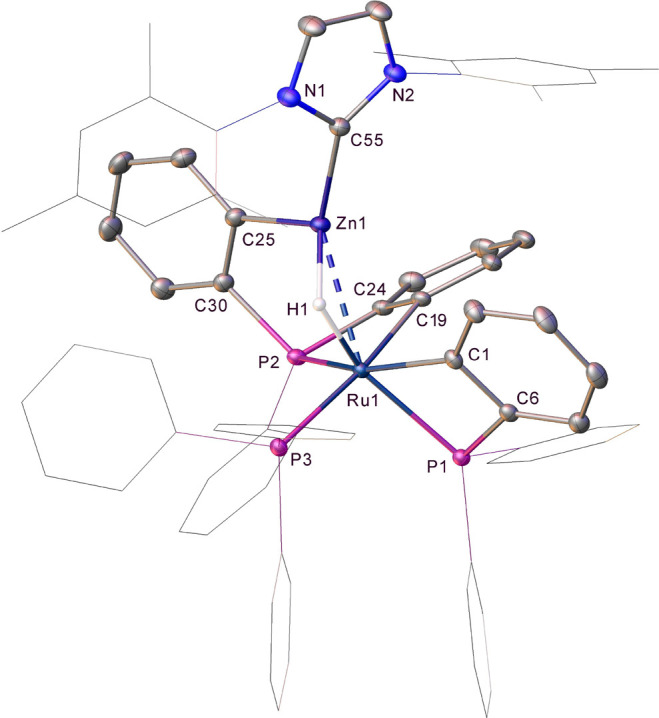
Molecular structure of **7**. Ellipsoids are represented at 30% probability. Hydrogen atoms (except for H1) and the solvent have been omitted for the sake of clarity.

The X-ray structure of **7** (Figure 5) showed a fac arrangement of an intact PPh_3_ (based on P3), one phosphine that was cyclometalated onto ruthenium (based on P1), and a third phosphine ligand (based on P2) unusually metalated through two phenyl rings, one onto Ru [Ru1–C19, 2.1438(16) Å] and the second onto Zn [Zn1–C25, 2.0206(18) Å]. The sixth transition metal coordination site was occupied by a hydride ligand bridging the Ru and Zn centers [Ru1···Zn1, 2.6541(3) Å]. Computational analysis of the bonding in **7** is reserved until after discussion of the product of the reaction of **3** with H_2_.

Redissolving a crystalline sample of the compound gave NMR signals for **7** together with a second, minor species, **8**.^46^ The signals for **7** were consistent with the solid state structure, a doublet of doublet of doublets Ru–H–Zn resonance in the ^1^H NMR spectrum, with one large (pseudotrans) and two smaller ^2^*J*_HP_ splittings, and one high-frequency ^31^P triplet (δ 52) for the PPh_3_ group, together with two lower-frequency (δ −12 and −24) doublet of doublet signals for the two cyclometalated phosphines.^47,48^ The presence of similar ^31^P chemical shifts for the minor species **8** supports it being an isomer. While ^1^H{^31^P} NMR measurements showed that both isomers feature hydride *trans* to a metalated phosphine, the presence of two surprisingly small ^2^*J*_HP_ couplings (9 and 6 Hz), in addition to a large, pseudotrans splitting (46 Hz) in the ^31^P-coupled ^1^H NMR spectrum, leaves it unclear as to exactly what the structure of **8** is. Closer inspection of NMR spectra recorded shortly after combining IMes and **2** indicates that **8** is formed in the initial stages (mixing for <15 min) and is thus a kinetic product of the reaction formed prior to subsequent growth of the thermodynamic product, **7**.^49^ The signals of **8** seen in the NMR spectra of **7** may therefore arise due to co-crystallization.

#### Reactivity of **2** and **3** toward H_2_

During our previous studies of Ru mono-Zn complexes,^12,13,19,20^ H_2_ was typically found to add across the Ru–Zn bond, as shown in Scheme 1b. Very different, contrasting behavior was seen with **2** and **3**. Thus, the former did not react with H_2_ at room temperature and, upon being heated to 60 °C, gave only a complex mixture of products. In contrast, **3** reacted rapidly with two molecules of H_2_ at room temperature to reverse the phosphine cyclometalation and form the cationic dizinc trihydride complex, [Ru(PPh_3_)_3_(ZnMe)_2_H_3_][BAr^F^_4_] (**12**). Remarkably, this transformation could also be carried out in the solid state simply by stirring a powdered sample of solid **3** under 1 atm of H_2_.

**Figure 6:**
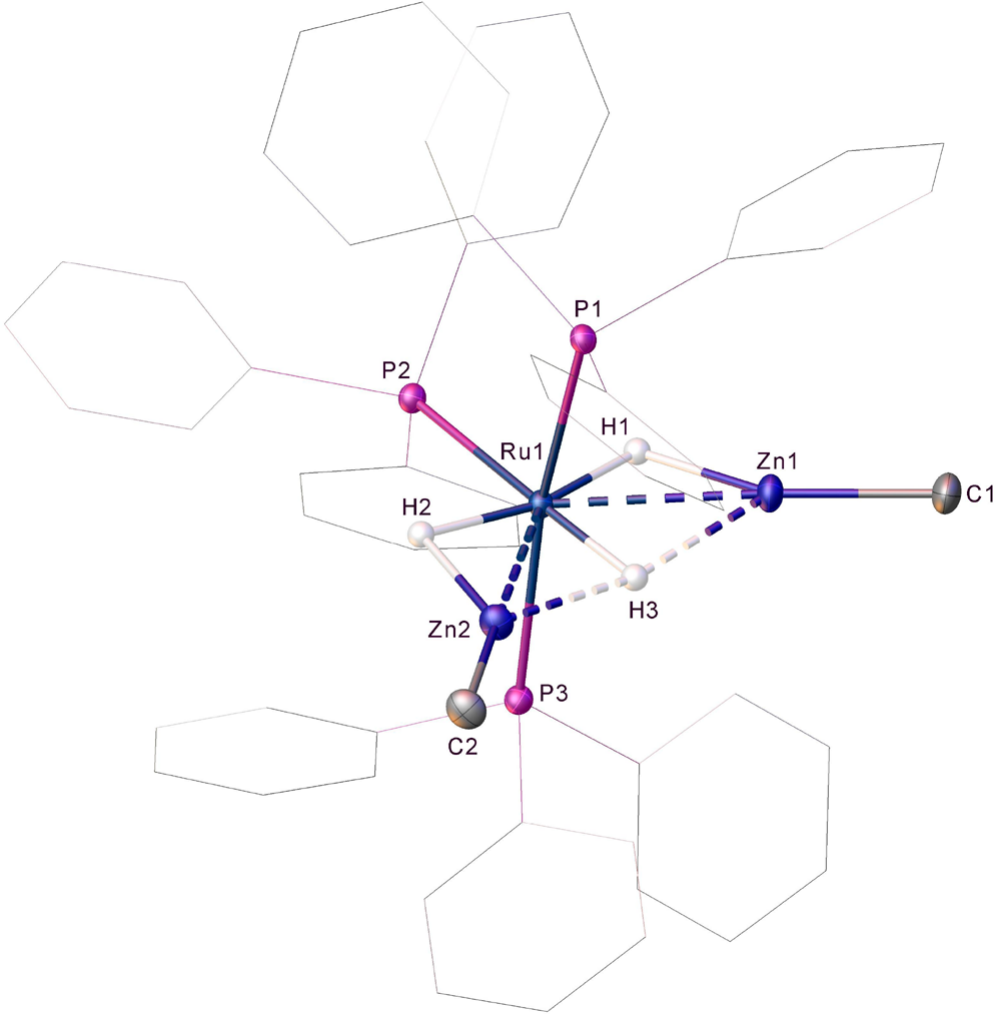
Molecular structure of the cation of **12**. Ellipsoids are represented at 30% probability. Phosphine and zinc methyl hydrogen atoms have been omitted for the sake of clarity.

Complex **12** displayed high-frequency doublet and triplet ^31^P NMR resonances, consistent with the mer-RuP_3_ geometry apparent in the X-ray crystal structure (Figure 6). The ^1^H NMR spectrum showed two hydride signals at δ −7.3 (dtd) and −11.1 (dtt) in a 2:1 ratio.^50^ Upon being heated to 60 °C, **12** decomposed as evidenced by the precipitation of an insoluble red oil at the bottom of reaction solutions.

The molecular structure of the cation in **12** is shown in Figure 6. The equatorial positions comprised two ZnMe ligands, one PPh_3_ ligand, and three hydrides (which were located and refined without restraints). The coordination sphere was completed by two phosphines in a distorted *trans*-axial arrangement [P1–Ru–P3, 164.804(17)°]. Two of the hydrides were disposed approximately *trans* to one another [H1–Ru1–H2, 163.3(16)°], while the third was located *trans* to the equatorial PPh_3_ ligand [H3–Ru1–P2, 175.7(12)°]. The two Ru–Zn distances [2.5188(3) and 2.5397(3) Å] are considered further in the computational analysis of **12** below.

### Structure and Bonding in **12** and **7**

**Figure 7:**
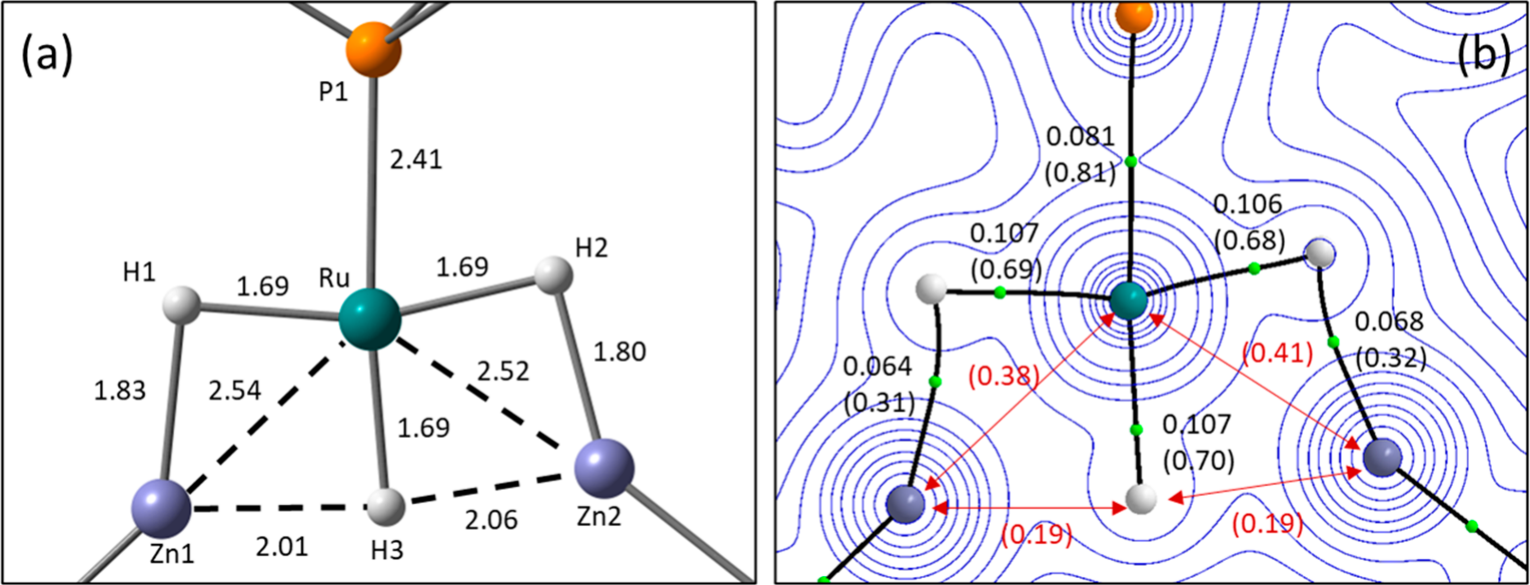
Electronic structure analyses of **12** highlighting key interactions in the {RuZn1Zn2} plane. (a) Computed structure of **12** (based on the experimental heavy atom positions with H atoms optimized with the BP86 functional). (b) QTAIM molecular graph with electron density contours shown in the {RuZn1Zn2} plane, with BCPs colored green with associated ρ(*r*) (au) and delocalization indices in parentheses. Delocalization indices between selected atoms not linked by a bond path are denoted in red.

The computed structure of **12** and details of the QTAIM analyses are shown in panels a and b, respectively, of Figure 7, with the related NCI and NOCV analyses presented in the Supporting Information. In this case, QTAIM reveals an absence of Ru–Zn bond paths, despite Ru–Zn distances that are similar to those in **2** and **3**. However, the computed DIs (Ru···Zn1, 0.38; Ru···Zn2, 0.41) indicate significant Ru···Zn interactions are still present, and this is supported by the NCI plot that shows blue stabilizing features between Ru and both Zn centers. For the outer hydrides, H1 and H2, the computed Ru–H distances of ∼1.69 Å are typical for a *trans* H–Ru–H arrangement and bond paths are characterized by a ρ(*r*) of ∼0.106 au and DIs of ∼0.69. These Ru–H bonds appear stronger than the Zn1–H1 and Zn2–H2 bonds [∼1.82 Å; ρ(*r*) ∼ 0.065 au; DI ∼ 0.31], and these are in turn significantly weaker than the unperturbed Zn–H bond in MeZnH [1.52 Å; ρ(*r*) = 0.11 au; DI = 0.89; see Supporting Information]. H1 and H2 are therefore bridging the respective Ru–Zn vectors but are strongly biased toward Ru over Zn. This is also reflected in the very low ellipticities of the Ru–H BCPs (average of 0.028) that are indicative of terminal hydride character, compared with the higher ellipticities of the Zn–H BCPs [average of 0.48 (Figure S42)].

The properties of the Ru–H3 bond [1.69 Å; ρ(*r*) = 0.107 au, DI = 0.70] are similar to those of the Ru–H1 and Ru–H2 bonds. In this case, no bond path to either Zn center is seen but DIs of 0.19 indicate some residual interactions are still present, and these are confirmed in the NCI plot that shows turquoise regions along the Zn1···H3 and Zn2···H3 vectors. The significant ellipticity of the Ru–H3 BCP (0.145) also suggests a distortion of the electron density away from a terminal Ru–H σ-bond due to the presence of the two {ZnMe}^+^ moieties.^51^ An NOCV analysis of {ZnMe}^+^ bonding in **12** indicates the major deformation density channels exhibit donation from Ru and both adjacent hydrides (see the Supporting Information).

A similar analysis of the bonding in compound **7** shows the hydride present, H1, to have characteristics similar to those of H1 and H2 in **12** [Ru–H1, 1.68 Å, ρ(*r*) = 0.108, DI = 0.68; Zn–H1, 1.79 Å, ρ(*r*) = 0.067, DI = 0.33]. The Ru–Zn distance of 2.65 Å is the longest of the species studied here, and no Ru–Zn bond path is computed; however, a Ru···Zn DI of 0.29 suggests some interaction between the two metal centers (Figure S41).

Overall, the {Ru(H1)Zn1(H3)} and {Ru(H2)Zn2(H3)} moieties in **12** and the {RuH1Zn} moiety in **7** can be considered as featuring asymmetrically bound bridging hydride ligands that interact more strongly with the Ru centers. A similar situation is seen in [Ru(IPr)_2_(CO)(ZnR)(η^2^-H_2_)(H)_2_]^+^ and [Ru(IPr)_2_(CO)(ZnR)(H)_2_]^+^ species (R = Et or Me),^12^ where, depending on the nature of the *trans* ligand, the hydride within a {RuHZn} moiety shows different degrees of Ru–H or Zn–H bonding character. These add to the continuum of structures that can be accessed in TM-MG heterobimetallic complexes featuring hydride ligands, the precise nature of which will depend on the coordination environment of the TM partner.^52,53^

## Conclusions

A combined computational and experimental study has been undertaken on two [RuZn_2_Me_2_] species, neutral **2** and cationic **3**. Geometrical considerations supported by computational analyses confirm the presence of direct Ru–Zn bonds in both species and suggest these are best formulated as Ru(ZnMe)_2_ complexes featuring discrete ZnMe ligands. Some additional stabilization may be achieved via Zn···Zn interactions, and **2** and **3** both exhibit Zn···C_aryl_ interactions, with these being more significant in **3**.

Experimentally, the two complexes exhibit diverse reactivities with thermolysis and the addition of a range of Lewis bases bringing about different outcomes with no apparent correlation to either the overall charges of the complexes or the different strengths of the Ru–Zn interactions present. **2** reacted with H_2_ to give a mixture of products, while in contrast, reaction of H_2_ with **3** led cleanly to [Ru(PPh_3_)_3_(ZnMe)_2_H_3_][BAr^F^_4_] (**12**). Computational analyses of this complex suggest the presence of three hydride ligands that bridge the Ru–Zn vectors asymmetrically toward Ru.

**12** adds to the range of transition metal complexes that feature multiple main group metals and multiple hydride ligands that have recently attracted a great deal of attention due to the unusual bonding interactions and unusual geometries they can possess.^54,55^ Studies of their reactivity, however, remain rare.^56^ In the study presented here, we have shown that both the TM and the MGM can be centers of reactivity in these heterobimetallic complexes and the factors that govern the site of reactivity will be the subject of future reports from our groups.

## Experimental Section

### General Comments

All manipulations were carried out under argon using standard Schlenk, high-vacuum, and glovebox techniques using dry and degassed solvents. C_6_D_6_ and THF-*d*_8_ were vacuum transferred from potassium. NMR spectra were recorded at 298 K (unless otherwise stated) on Bruker Avance 400 and 500 MHz NMR spectrometers and referenced as follows: C_6_D_6_ (^1^H, δ 7.16; ^13^C, δ 128.0), THF-*d*_8_ (^1^H, δ 1.72; ^13^C, δ 25.3), and toluene-*d*_8_ (^1^H, δ 2.09). ^31^P spectra were referenced externally to 85% H_3_PO_4_ (δ 0.0). Elemental analyses were performed by Elemental Microanalysis Ltd. (Okehampton, Devon, U.K.). Compounds **1**,^13^**2**,^14^**3**,^13^ and IMes^57^ were prepared according to literature methods.

### [Ru(P^n^Bu_3_)(C_6_H_4_PPh_2_)_2_(ZnMe)_2_] (**6**)

C_6_D_6_ (0.5 mL) was added to a mixture of **2** (40 mg, 0.038 mmol) and P^n^Bu_3_ (9.5 μL, 0.038 mM) in a J. Young’s resealable NMR tube. After 15 h at room temperature, the volatiles were removed and the resulting solid was recrystallized from benzene/hexane. The microcrystalline solid was washed with hexane and dried under vacuum to give **6** as a yellow solid (18 mg, 48%). ^1^H NMR (500 MHz, C_6_D_6_): δ 8.21–8.16 (m, 2H, Ar), 7.97–7.92 (m, 2H, Ar), 7.91–7.86 (m, 2H, Ar), 7.76 (m 1H, Ar), 7.26 (tt, *J* = 7.6 Hz, *J* = 1.5 Hz, 1H, Ar), 7.18 (dt, *J* = 7.6 Hz, *J* = 1.5 Hz, 2H, Ar), 7.11–7.04 (m, 2H, Ar), 7.03–6.85 (br, 8H, Ar), 6.84–6.72 (m, 4H, Ar), 6.54–6.49 (m, 2H, Ar), 6.40–6.35 (m, 2H, Ar), 1.71–1.52 (br m, 6H, PC*H*_2_), 1.35–1.02 (br m, 12 H, PCH_2_C*H*_2_C*H*_2_), 0.84 (t, ^3^*J*_HH_ = 7.5 Hz, 9H, PCH_2_CH_2_CH_2_C*H*_3_), 0.47 (s, 3H, ZnMe), −0.64 (s, 3H, ZnMe). ^31^P{^1^H} NMR (202 MHz, C_6_D_6_): δ 21.3 (dd, ^2^*J*_PP_ = 246 Hz, ^2^*J*_PP_ = 17 Hz), −23.2 (dd, *J*_PP_ = 25 Hz, *J*_PP_ = 17 Hz), −26.3 (dd, ^2^*J*_PP_ = 246 Hz, ^2^*J*_PP_ = 25 Hz). ^13^C{^1^H} NMR (126 MHz, C_6_D_6_): δ 173.9 (dt, *J*_CP_ = 13 Hz, *J*_CP_ = 10 Hz, *C*_quaternary_), 158.4 (dd, *J*_CP_ = 47 Hz, *J*_CP_ = 4 Hz, *C*_quaternary_), 158.2 (dd, *J*_CP_ = 48 Hz, *J*_CP_ = 9 Hz, *C*_quaternary_), 152.8 (dd, *J*_CP_ = 47 Hz, *J*_CP_ = 2 Hz, *C*_quaternary_), 140.7 (dt, *J*_CP_ = 20 Hz, *J*_CP_ = 3 Hz, aryl *C*H), 140.5–140.0 (m, *C*_quaternary_), 138.1 (d, *J*_CP_ = 21 Hz, aryl *C*H), 133.6 (br d, *J*_CP_ = 12 Hz, aryl *C*H), 132.7 (d, *J*_CP_ = 9 Hz, aryl *C*H), 132.3 (d, *J*_CP_ = 11 Hz, aryl *C*H), 131.9 (d, *J*_CP_ = 10 Hz, CH), 130.8 (br d, *J* = 4 Hz, aryl *C*H), 130.6 (app t, *J*_CP_ = 3 Hz, aryl *C*H), 130.0 (br s, aryl *C*H), 128.8 (br d, *J*_CP_ = 16 Hz, aryl *C*H), 128.6 (d, *J*_CP_ = 9 Hz, aryl *C*H), 128.3 (d, *J*_CP_ = 9 Hz, aryl *C*H), 127.3 (d, *J*_CP_ = 9 Hz, aryl *C*H), 127.1 (br s), 127.0 (br s), 124.2 (d, *J*_CP_ = 7 Hz, aryl *C*H), 122.0 (d, *J*_CP_ = 8 Hz, aryl *C*H), 31.1 (br m, *J*_CP_ = 22 Hz, *C*H_2_), 26.8 (d, *J*_CP_ = 3 Hz, *C*H_2_), 24.8 (d, *J*_CP_ = 11 Hz, *C*H_2_), 14.0 (s, *C*H_3_), 11.5 (br m, Zn*C*H_3_), −3.6 (s, Zn*C*H_3_). Anal. Found (%): C, 60.51; H, 6.22. Calcd for C_50_H_61_P_3_RuZn_2_: C, 60.86; H, 6.23.

### [Ru(PPh_3_)(C_6_H_4_PPh_2_)(PPh(C_6_H_4_)_2_Zn(IMes))H] (**7**)

A mixture of **2** (40 mg, 0.037 mmol) and IMes (23 mg, 0.074 mmol) was added to a J. Young’s resealable NMR tube. Addition of C_6_D_6_ (0.5 mL) led to an instantaneous change in color from red to orange-yellow. NMR spectroscopy revealed that consumption of the starting material took place over 3 h to give **7** as the main product. Removal of the volatiles under reduced pressure and recrystallization of the residue from benzene/hexane gave yellow crystals of **7**, which were washed with hexane and dried under vacuum (33 mg, 70% yield). ^1^H NMR (500 MHz, THF-*d*_8_): δ 7.41 (s, 2H, Ar), 7.38 (br d, *J* = 6.8 Hz, 1H, Ar), 7.27 (t, *J* = 7.1 Hz, 1H, Ar), 7.22–7.14 (m, 4H, Ar), 7.11–7.07 (m, 1H, Ar), 7.02 (t, *J* = 8.0 Hz, 2H, Ar), 6.95–6.62 (m, 22H, Ar), 6.60–6.39 (m, 11H, Ar + NCH=C*H*N), 6.12 (t, *J* = 8.0 Hz, 1H, Ar), 5.87 (t, *J* = 7.5 Hz, 1H, Ar), 5.64 (m, 1H, Ar), 5.42 (t, *J* = 7.5 Hz, 1H, Ar), 2.16 (s, 6H, C_6_*Me*_3_H_3_), 2.11 (s, 6H, C_6_*Me*_3_H_3_), 1.92 (s, 6H, C_6_*Me*_3_H_3_), −10.50 (ddd, ^2^*J*_HP_ = 53.5 Hz, ^2^*J*_HP_ = 23.0 Hz, ^2^*J*_HP_ = 3.5 Hz, 1H, Ru*H*). ^31^P{^1^H} NMR (162 MHz, C_6_D_6_): δ 52.4 (t, ^2^*J*_PP_ = 25 Hz), −11.6 (dd, ^2^*J*_PP_ = 27 Hz, ^2^*J*_PP_ = 15 Hz), −24.0 (dd, ^2^*J*_PP_ = 25 Hz, ^2^*J*_PP_ = 15 Hz). Selected ^13^C{^1^H} NMR (126 MHz, C_6_D_6_): δ 182.9 (br d, *J*_CP_ = 3 Hz, *C*_NHC_), 176.9 (br dm, *J*_CP_ = 65 Hz, *C*_ipso_), 168.8 (dt, *J*_CP_ = 40 Hz, *J*_CP_ = 4 Hz, *C*_ipso_), 166.5 (d, ^1^*J*_CP_ = 59 Hz, *C*_ipso_), 160.6 (dd, ^1^*J*_CP_ = 56 Hz, ^3^*J*_CP_ = 12 Hz, *C*_ipso_), 154.4 (dd, *J*_CP_ = 48 Hz, *J*_CP_ = 4 Hz, *C*_ipso_), 148.3 (d, ^1^*J*_CP_ = 36 Hz, *C*_ipso_), 21.0 (s, C_6_*Me*_3_H_3_), 19.0 (s, C_6_*Me*_3_H_3_), 18.5 (s, C_6_*Me*_3_H_3_). Selected NMR data for **8**. ^1^H NMR (500 MHz, C_6_D_6_): δ −9.03 (ddd, ^2^*J*_HP_ = 45.9 Hz, ^2^*J*_HP_ = 8.8 Hz, ^2^*J*_HP_ = 6.1 Hz, 1H, Ru*H*). ^31^P{^1^H} NMR (162 MHz, C_6_D_6_): δ 57.4 (t, ^2^*J*_PP_ = 18 Hz), −26.9 (t, ^2^*J*_PP_ = 20 Hz), −30.9 (app t, ^2^*J*_PP_ = 18 Hz). Anal. Found (%): C, 72.97; H, 5.46; N, 1.99. Calcd for C_75_H_67_N_2_P_3_RuZn·0.4C_6_H_6_·0.1C_6_H_14_: C, 72.31; H, 5.51; N, 2.16 [NMR spectroscopy confirmed the presence of benzene and hexane (Figure S31)].

### Thermal Decomposition of **2**

A C_6_D_5_CD_3_ solution (0.5 mL) of **2** (10 mg, 0.01 mmol) was heated at 80 °C for 50 h, affording a dark red-brown colored solution. ^31^P{^1^H} NMR spectroscopy showed complete consumption of the starting material together with the formation of **5**(20) and a new species **4**. Selected NMR data for **4**. ^31^P{^1^H} NMR (162 MHz, C_6_D_5_CD_3_): δ 87.0 (t, ^2^*J*_PP_ = 25 Hz), 49.3 (dd, ^2^*J*_PP_ = 249 Hz, ^2^*J*_PP_ = 24 Hz), 44.4 (dd, ^2^*J*_PP_ = 249 Hz, ^2^*J*_PP_ = 26 Hz).

### Thermal Decomposition of **3**

A C_6_D_6_ solution (0.5 mL) of **3** (40 mg, 0.02 mmol) was heated at 80 °C for 2 days. ^31^P{^1^H} NMR spectroscopy showed no remaining starting material and the formation of two doublet resonances for a new product **9** at δ 77.5 (*J*_PP_ = 38 Hz) and δ 47.7 (*J*_PP_ = 38 Hz). When a second sample was prepared and heated at 110 °C for 3 days, ^31^P{^1^H} NMR spectroscopy revealed total consumption of **3** and the appearance of two doublets at δ 52.8 (*J*_PP_ = 31 Hz) and δ 46.5 (*J*_PP_ = 31 Hz), which we assign to a second product, **10**.

### Reaction of **3** with Lewis Bases

(i) PBu_3_ (2.6 μL, 0.01 mmol) was added to a C_6_D_6_ (0.5 mL) solution of **3** (20 mg, 0.01 mmol) in a J. Young’s resealable NMR tube to give a homogeneous red solution. After ∼15 min, deposition of an unknown, insoluble red oil started to occur. A ^1^H NMR spectrum of the sample at this time showed the presence of a doublet of doublet of doublets hydride signal at δ −8.25 corresponding to **1** and a second doublet of doublet of doublets hydride signal at δ −9.07 (^2^*J*_HP_ = 50.9, 21.8, and 12.3 Hz), for [Ru(P^n^Bu_3_)(C_6_H_4_PPh_2_)_2_(ZnMe)H] (**11**). (ii) PCy_3_ (3 mg, 0.02 mmol) and **3** (20 mg, 0.01 mmol) were dissolved in C_6_D_6_ (0.5 mL) in a J. Young’s resealable NMR tube to give a homogeneous red solution. After ∼15 min, deposition of an unknown, insoluble red oil started to occur. A ^1^H NMR spectrum of the sample at this time showed the presence of a doublet of doublet of doublets hydride signal at δ −8.25 corresponding to **1**. (iii) IMes (3 mg, 0.01 mmol) and **3** (20 mg, 0.01 mmol) were dissolved in C_6_D_6_ (0.5 mL) in a J. Young’s resealable NMR tube to give a homogeneous red solution. After ∼30 min, deposition of an unknown, insoluble red oil started to occur. A ^1^H NMR spectrum of the solution displayed resonances for **3** alongside the diagnostic hydride of **1**. Addition of a second equivalent of IMes (3 mg, 0.01 mmol) afforded full conversion of **3** to **1**. Isolation of a small number of crystals confirmed the same unit cell parameters reported for **1**.

### [Ru(PPh_3_)_3_(ZnMe)_2_H_3_][BAr^F^_4_] (**12**)

A J. Young’s resealable ampule was charged with a C_6_H_6_ (5 mL) suspension of **3** (96 mg, 0.05 mmol). After being gently heated to fully dissolve the solid, the resulting red solution was degassed (three freeze–pump–thaw cycles) and H_2_ (1 atm) added with vigorous stirring. After 5 min, this gave a pale-yellow solution, which upon treatment with hexane (5 mL) afforded a pale-yellow crystalline sample of **12**. This was collected and dried under vacuum. Yield: 76 mg (79%). An alternative route to **12** involved stirring a solid sample of **3** under H_2_ (1 atm) for ∼2 h, by which time the sample had changed color from red-orange to off-white. A ^31^P NMR spectrum in C_6_D_6_ revealed complete conversion to **12**. ^1^H NMR (500 MHz, C_6_D_6_): δ 8.47 (s, 8H, BAr^F^_4_), 7.71 (s, 4H, BAr^F^_4_), 7.07 (br t, 1H, *J* = 9.1 Hz, 6H, Ar), 7.04–6.99 (m, 12H, Ar), 6.85 (t, *J* = 7.3 Hz, 9H, Ar), 6.78 (t, *J* = 7.4 Hz, 12H, Ar), 6.71 (td, *J* = 7.8 Hz, *J* = 1.4 Hz, 6H, Ar), −1.02 (s, 6H, Zn*Me*), −7.34 (dtd, ^2^*J*_HP_ = 16.1 Hz, ^2^*J*_HP_ = 12.5 Hz, ^2^*J*_HH_ = 3.3 Hz, 2H, Ru*H*), −11.06 (dtt, ^2^*J*_HP_ = 39.5 Hz, ^2^*J*_HP_ = 18.1 Hz, ^2^*J*_HH_ = 3.3 Hz, 1H, Ru*H*). ^31^P{^1^H} NMR (202 MHz, C_6_D_6_): δ 47.4 (t, ^2^*J*_PP_ = 26 Hz), 41.8 (d, ^2^*J*_PP_ = 26 Hz). ^13^C{^1^H} NMR (126 MHz, C_6_D_6_): δ 162.8 (1:1:1:1 q, ^1^*J*_CB_ = 50 Hz, BAr^F^_4_), 135.6–135.1 (m), 133.9 (d, *J*_CP_ = 11 Hz, *C*_ortho_-PPh_3_), 133.7–133.2 (m, 133.6, *C*_*ipso*_-PPh_3_ overlapped with 133.4, vt, *J*_CP_ = 6 Hz, *C*_*ortho*_-PPh_3_), 131.3 (s, *C*_para_-PPh_3_), 130.4–129.5 (m, 130.3, s, *C*_para_-PPh_3_ overlapped with 130.0, qq, *J*_CF_ = 32 Hz, *J*_CF_ = 3 Hz, BAr^F^_4_), 129.2 (vt, *J*_CP_ = 5 Hz, *C*_meta_-PPh_3_), 128.3 (d, *J*_CP_ ∼ 5 Hz, *C*_meta_-PPh_3_, overlapped with C_6_D_6_), 125.3 (q, ^1^*J*_CF_ = 273 Hz, BAr^F^_4_), 118.1 (s, BAr^F^_4_). −5.7 (s, Zn*Me*). Anal. Found (%): C, 55.19; H, 3.48. Calcd for C_88_H_66_BF_24_Zn_2_P_3_Ru: C, 55.19; H, 3.47.

### X-ray Crystallography

Data for **6**, **7**, and **12** were obtained using an Agilent SuperNova instrument and a Cu Kα radiation source. All experiments were conducted at 150 K, and models refined using SHELXL^58^ via the Olex2^59^ interface. Refinements were largely straightforward, and only points of note will be detailed herein. First, the phenyl rings based on C9 and C15 were treated for 80:20 disorder in the structure of **6**. In **7**, the asymmetric unit was seen to contain one molecule of the bimetallic complex and two molecules of benzene. The hydride in the former was located and refined without restraints as were the hydride ligands in **12**. Unsurprisingly, the anion in the latter structure required some disorder modeling. In particular, fluorine atoms F4–F6 were treated for three-way disorder in a 0.425:0.425:0.15 ratio, while F22–F24 were modeled to take into account 50:50 disorder. Distances and ADP restraints were employed in disordered regions, to assist convergence.

### Computational Details

Density functional theory calculations were performed with Gaussian 16 (revision C.01).^60^ Ru, Zn, and P centers were described with the Stuttgart RECPs and associated basis sets,^61^ and 6-31G** basis sets were used for all other atoms.^62,63^ A set of d orbital polarization functions was also added to P (ζ^d^ = 0.387).^64^ Electronic structure analyses were performed on geometries using the heavy atom positions derived from the crystallographic studies with H atom positions optimized with the BP86 functional.^65,66^ Details of functional testing on the fully optimized structure of **2** are provided in the Supporting Information. Quantum theory of atoms in molecules (QTAIM)^67^ used the AIMALL program.^68^ NCI calculations were based on the promolecular densities and used NCIPLOT^69^ with visualization via VMD.^70^ Natural orbitals for chemical valence (NOCV) analyses^71^ were performed using the Amsterdam Modeling Suite (AMS) package.^72^ Computed geometries are displayed with ChemCraft,^73^ and all geometries are supplied as a separate XYZ file (Supporting Information).

## Supporting Information Available

NMR spectra of products from reactions of **2** and **3**, X-ray structural data and metrics for **6**, **7**, and **12**, and computational details (functional testing, electronic structure analyses, QTAIM, and NOCV) (PDF)
Structures and Cartesian coordinates (XYZ)

## Supplementary Material

ic1c02072_si_001.pdf

ic1c02072_si_002.xyz

## Accession Codes

CCDC 2083542–2083544 contain the supplementary crystallographic data for this paper. These data can be obtained free of charge via www.ccdc.cam.ac.uk/data_request/cif, or by emailing data_request@ccdc.cam.ac.uk, or by contacting The Cambridge Crystallographic Data Centre, 12 Union Road, Cambridge CB2 1EZ, UK; fax: +44 1223 336033.

## Dedication

The authors dedicate this paper to Professor Christian Bruneau in recognition of his outstanding contributions to ruthenium chemistry.
