# Supplementary material for: Bonding and Reactivity of a Pair of Neutral and Cationic Heterobimetallic RuZn2 Complexes
Source: Inorg Chem. 2021 Oct 18;60(21):16256–65. doi: 10.1021/acs.inorgchem.1c02072 (PMC8730504; doi:10.1021/acs.inorgchem.1c02072)
Supplement: Supplementary file 1 — ic1c02072_si_001.pdf [file ic1c02072_si_001.pdf]

SUPPORTING INFORMATION

Bonding and Reactivity of a Pair of Neutral and Cationic Heterobimetallic $RuZn_2$ Complexes

Fedor M. Miloserdov, Anne-Frédérique Pécharman, Lia Sotorrios, Nasir A. Rajabi, John P. Lowe, Stuart A. Macgregor, Mary F. Mahon and Michael K. Whittlesey

Department of Chemistry, University of Bath, Claverton Down, Bath BA2 7AY, U.K., Institute of Chemical Sciences, Heriot-Watt University, Edinburgh EH14 4AS, U.K. and Laboratory of Organic Chemistry, Wageningen University, Stippeneng 4, Wageningen 6708 WE, The Netherlands

Table of Contents

S-1 NMR spectra S2

S-2 Computational Studies S41

S-3 Computed Geometries……………………………………………………………S56

S-4 References..S74

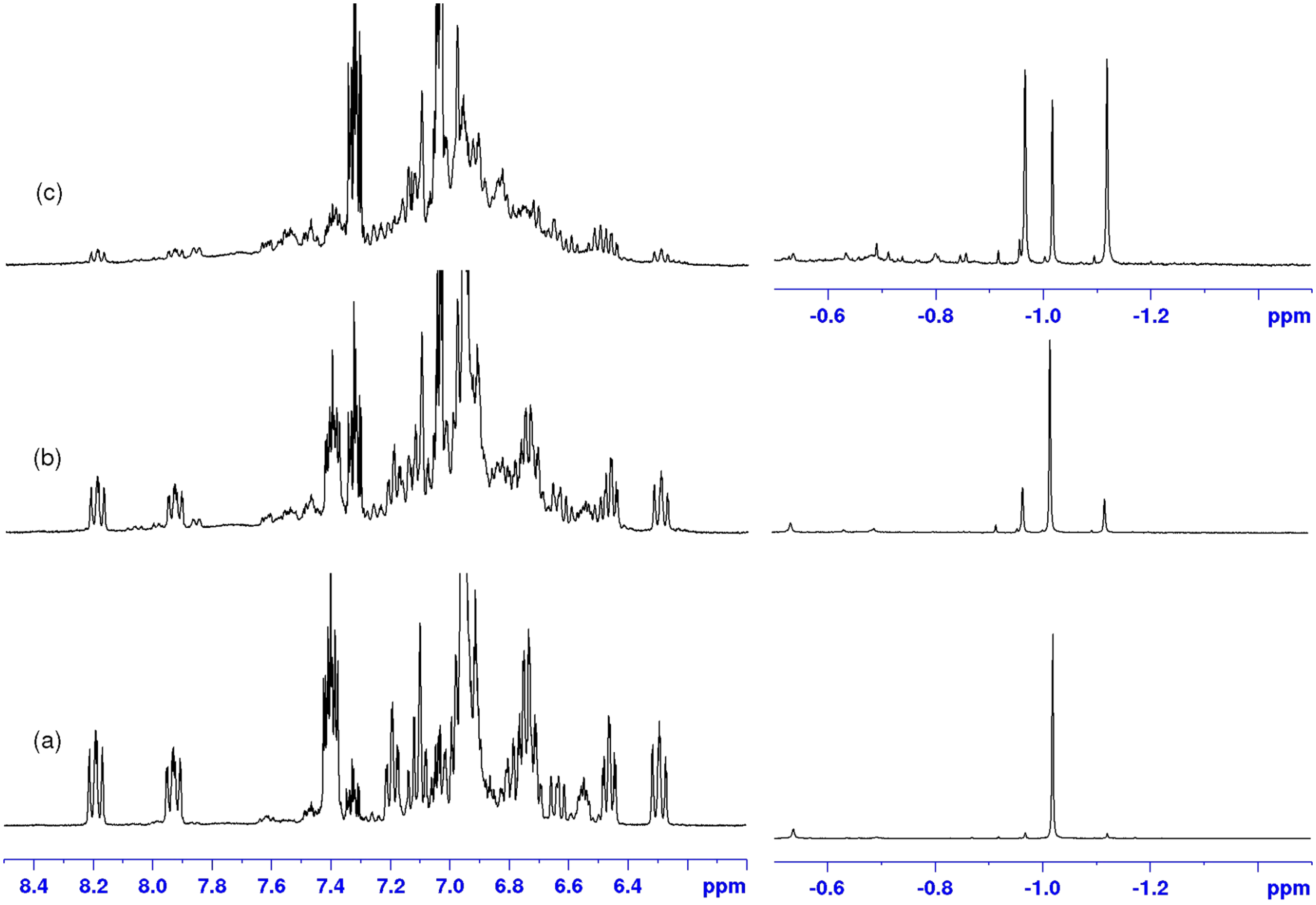

Figure S1. ^{1}H NMR spectra (400 MHz, 298 K) upon heating [Ru(PPh$_3$)(C$_6$H$_4$PPh$_2$)$_2$(ZnMe)$_2$] (**2**) in C$_6$D$_5$CD$_3$ at 80 ºC for (a) 2 h, (b) 11 h and (c) 30 h.

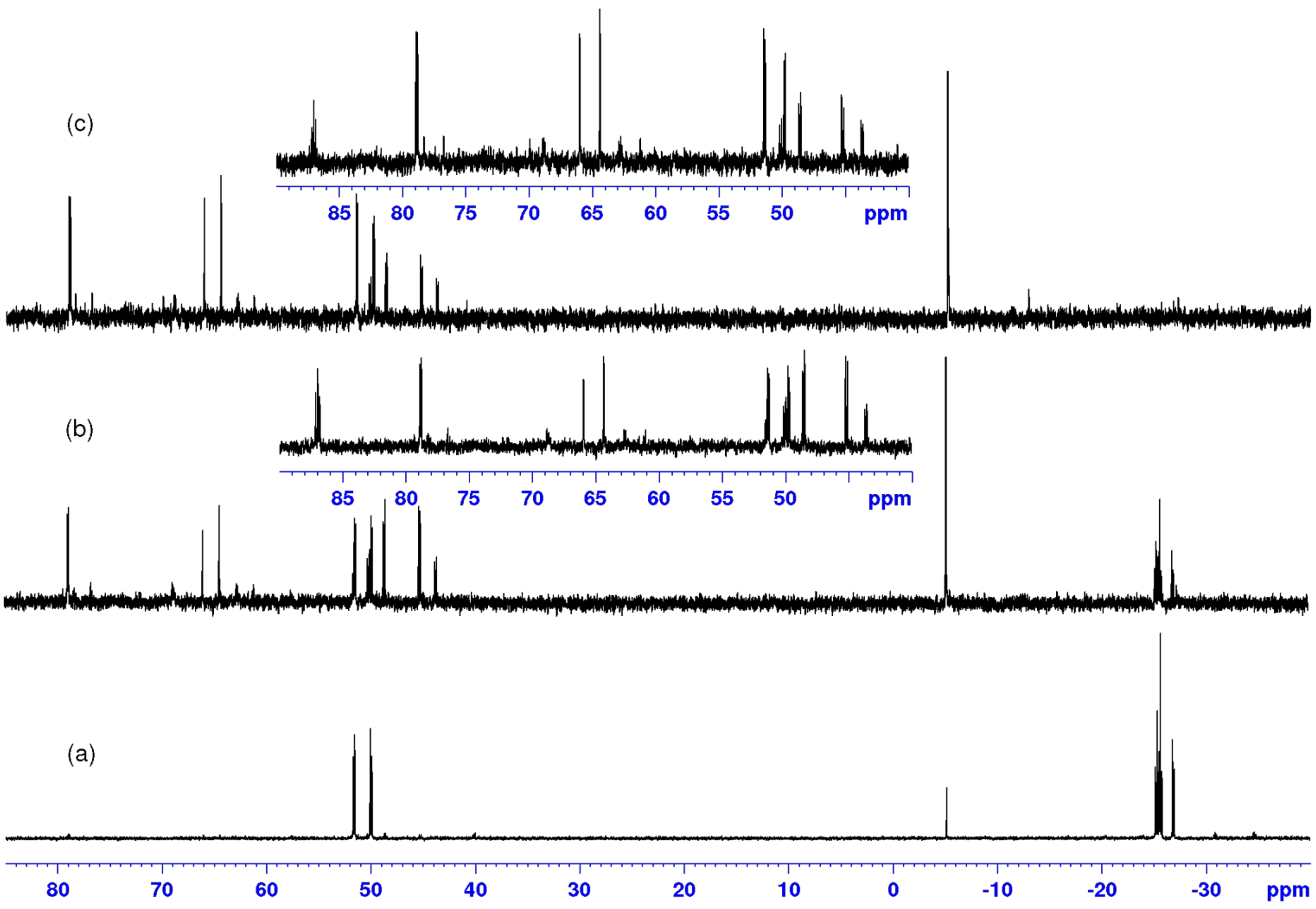

Figure S2. $^{31}P\{^1H\}$ NMR spectra (162 MHz, 298 K) upon heating [Ru(PPh$_3$)(C$_6$H$_4$PPh$_2$)$_2$(ZnMe)$_2$] (**2**) in $C_6D_5CD_3$ at 80 ºC for (a) 2 h, (b) 30 h and (c) 55 h. Insets of high frequency regions shown in (b) and (c) - see Figures S3 and S4 for assignments.

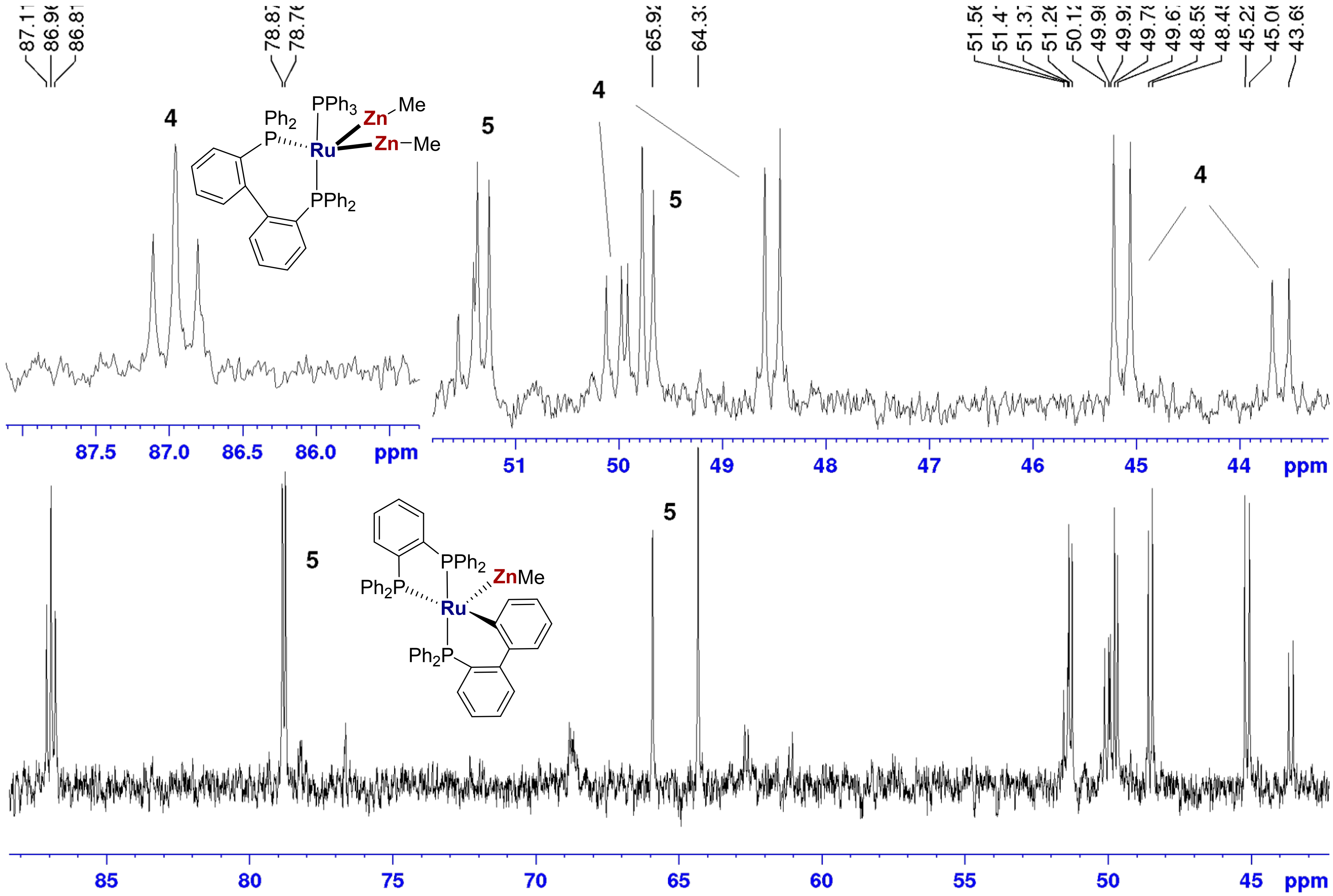

Figure S3. $^{31}P\{^{1}H\}$ NMR spectrum (162 MHz, 298 K) upon heating [Ru(PPh$_3$)(C$_6$H$_4$PPh$_2$)$_2$(ZnMe)$_2$] (**2**) in C$_6$D$_5$CD$_3$ at 80 ºC for 30 h highlighting the signals arising from the proposed intermediate [Ru(BIPHEP)(PPh$_3$)(ZnMe)$_2$] (**4**) and [Ru(dppbz)(PPh$_2$(biphenyl))′(ZnMe)] (**5**).

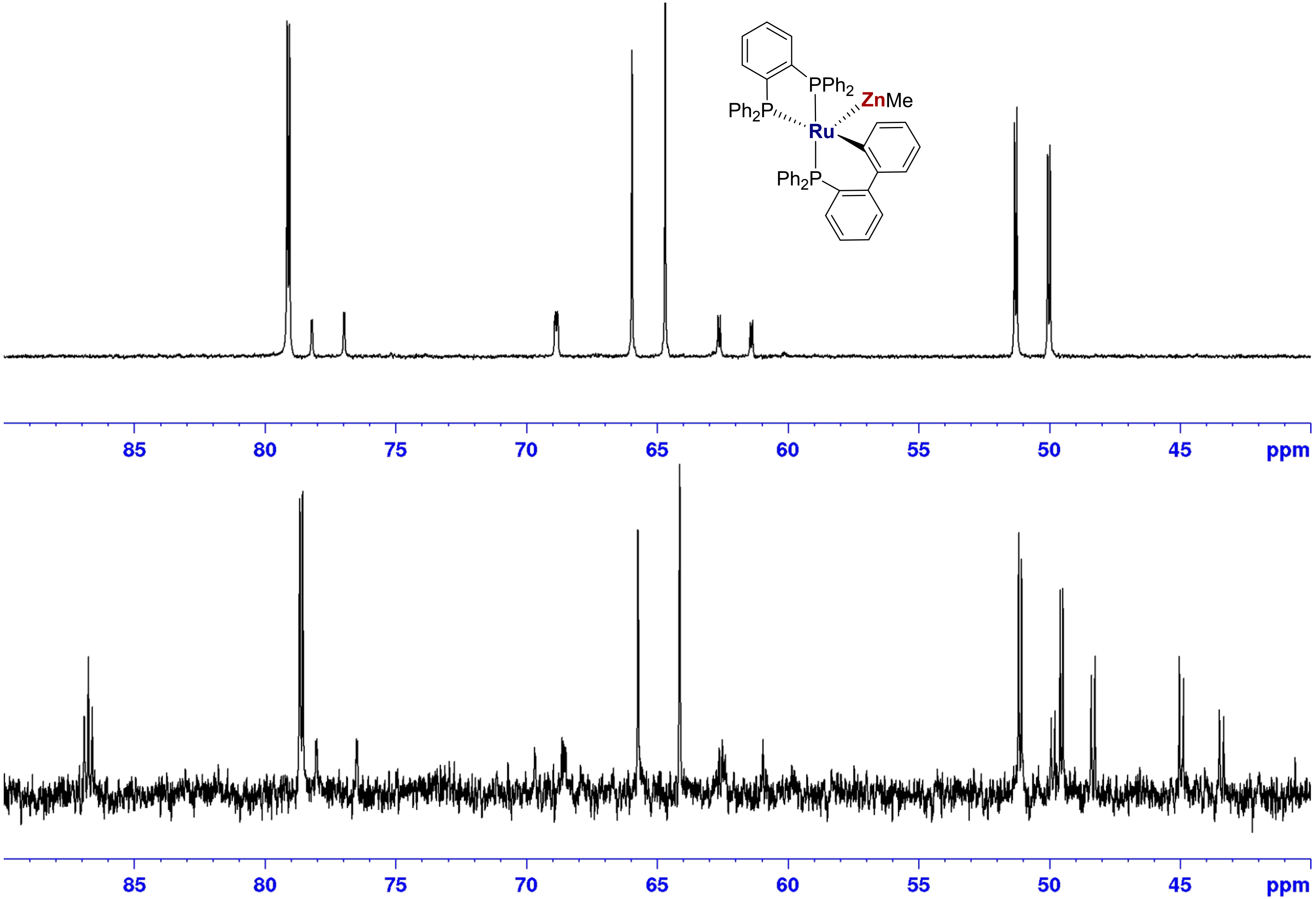

Figure S4. $^{31}P\{^1H\}$ NMR signals (162 MHz) from (bottom) heating [Ru(PPh$_3$)(C$_6$H$_4$PPh$_2$)$_2$(ZnMe)$_2$] (**2**) in C$_6$D$_5$CD$_3$ at 80 ºC for 55 h compared to (top) those of isolated [Ru(dppbz)(PPh$_2$(biphenyl))′(ZnMe)] (**5**: C$_6$D$_6$, 162 MHz, 298 K). Major and minor isomers account for all the signals in the top spectrum.[1]

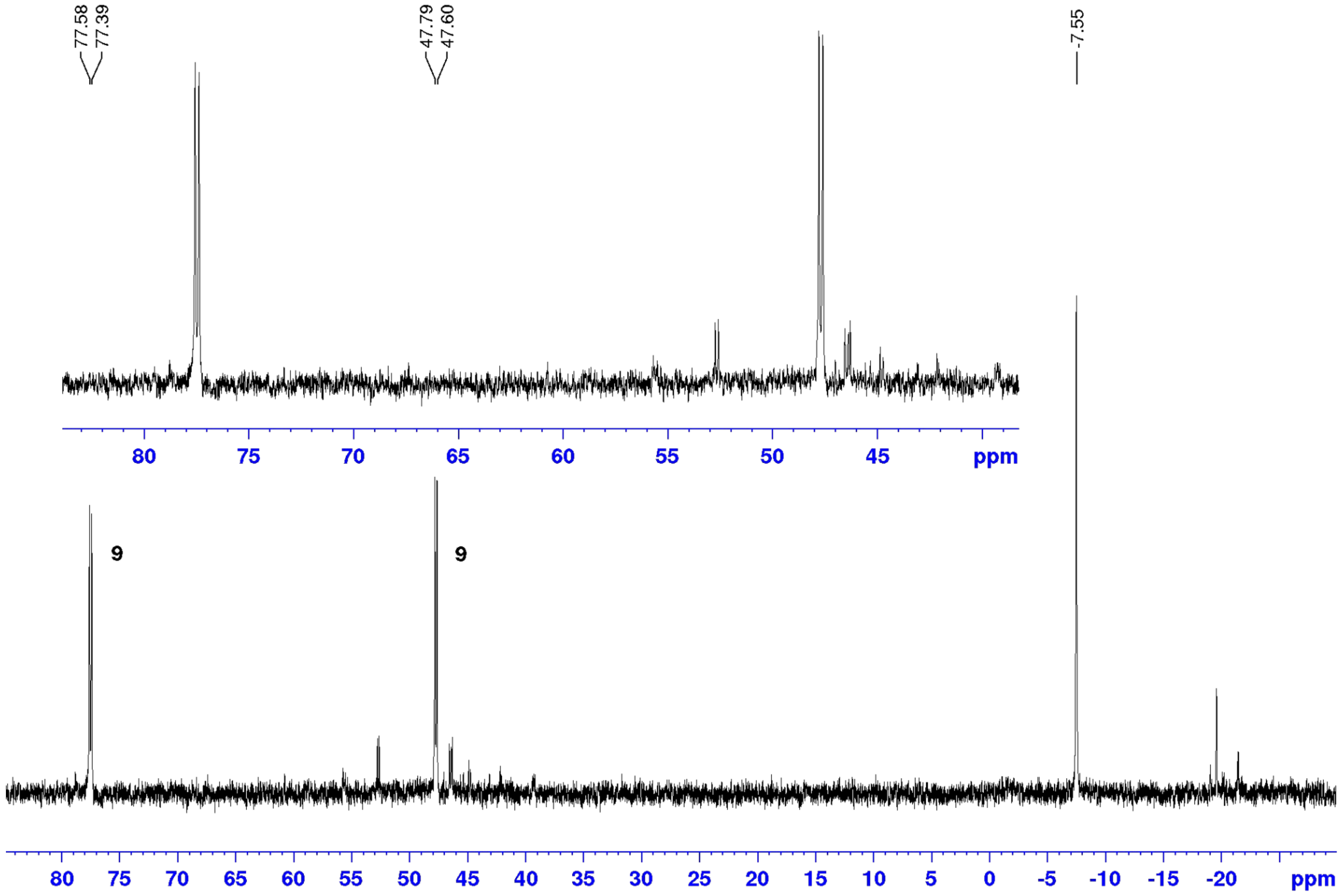

Figure S5. $^{31}P\{^{1}H\}$ NMR spectrum (202 MHz, 298 K) after heating $[Ru(PPh_3)_2(C_6H_4PPh_2)(ZnMe)_2][BAr^F{}_4]$ (**3**) for 2 days at 80 ºC in C_6D_6 showing the two doublet signals for the unknown product **9**.

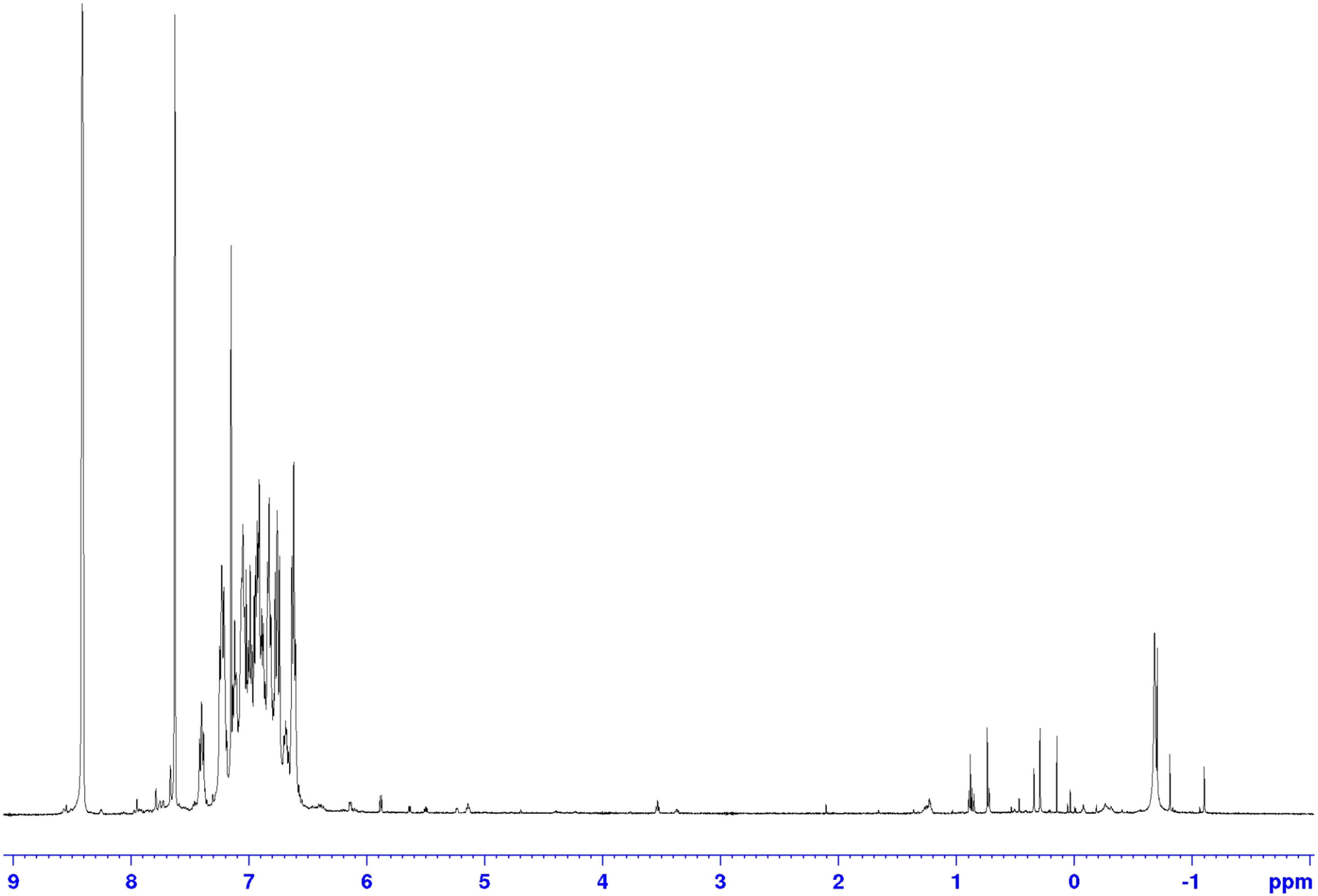

Figure S6. ^{1}H NMR spectrum (500 MHz, 298 K) after heating [Ru(PPh_3)$_2$($C_6H_4PPh_2$)(ZnMe)$_2$][BAr$^F{}_4$] (**3**) for 2 days at 80 ºC in C_6D_6. The spectrum highlights the absence of diagnostic signals to help in the assignment of **9**.

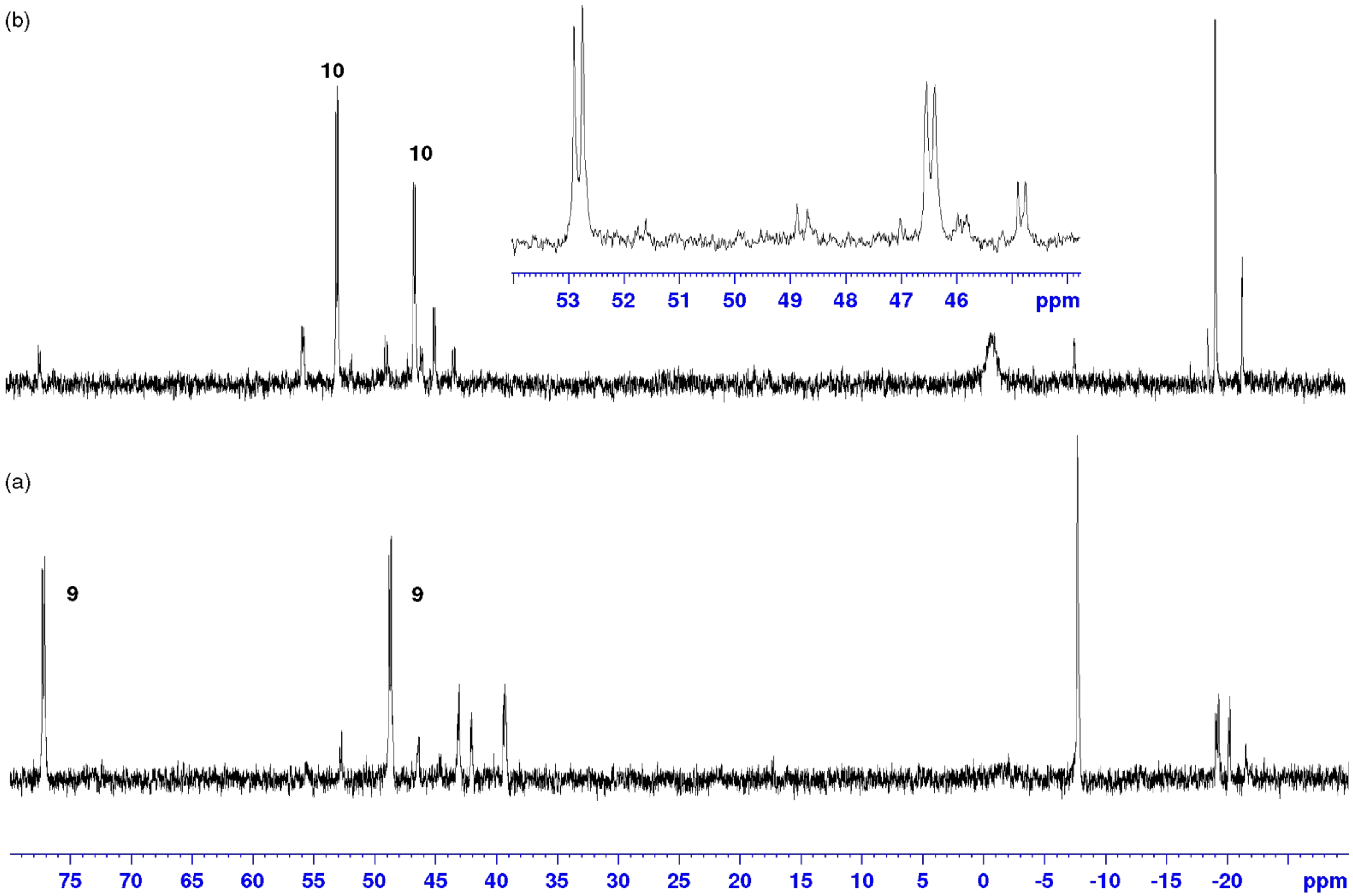

Figure S7. $^{31}P\{^1H\}$ NMR spectra (202 MHz, 298 K) showing the formation of **9** and subsequent transformation to **10** (signal of which are highlighted in the inset) upon heating $[Ru(PPh_3)_2(C_6H_4PPh_2)(ZnMe)_2][BAr^F{}_4]$ **(3)** at 110 ºC in toluene-d_8 for (a) 3 h and (b) 3 days.

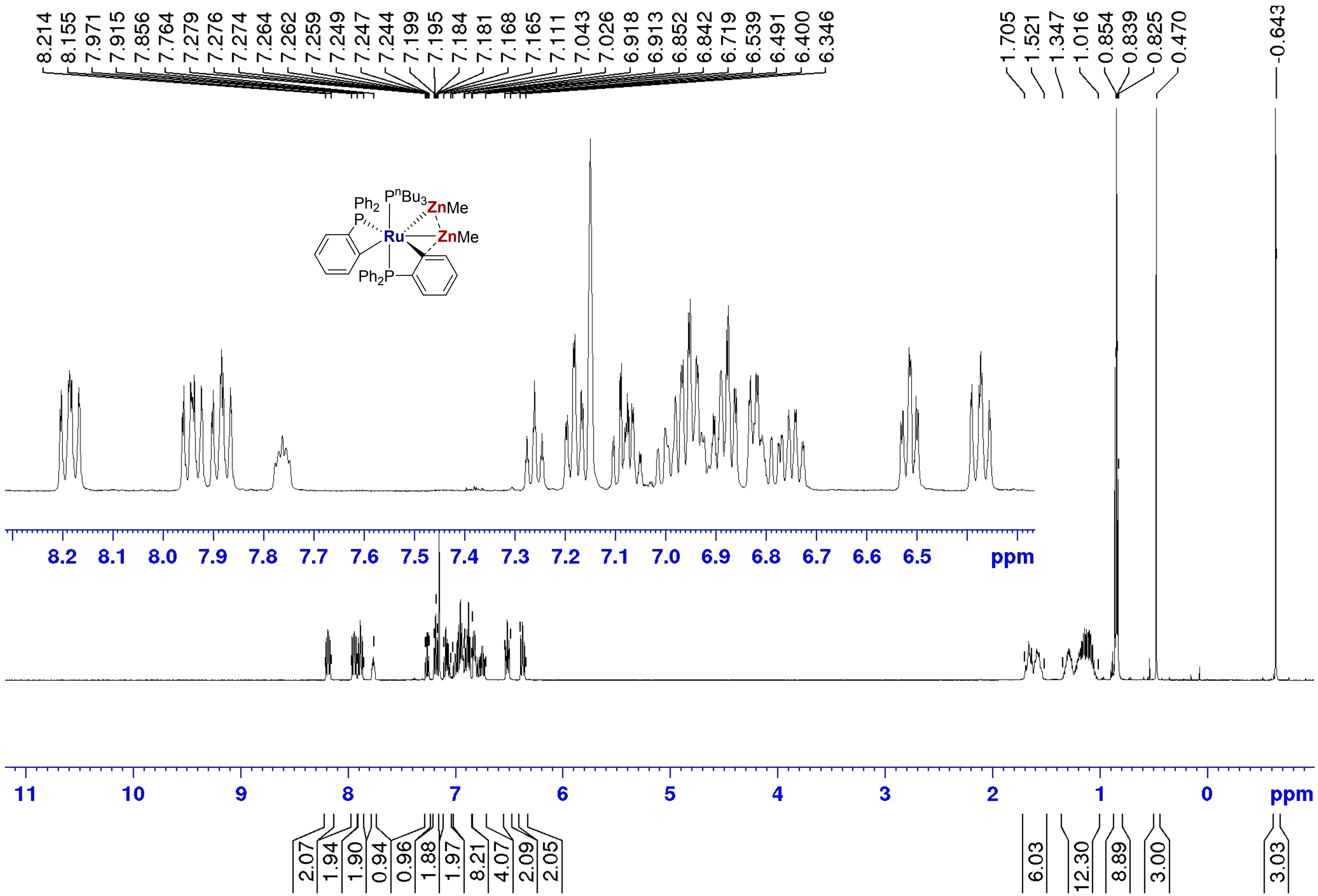

Figure S8. ^{1}H NMR spectrum (500 MHz, C_6D_6, 298 K) of $Ru(PBu_3)(C_6H_4PPh_2)_2(ZnMe)_2$ (**6**), with inset of high frequency region.

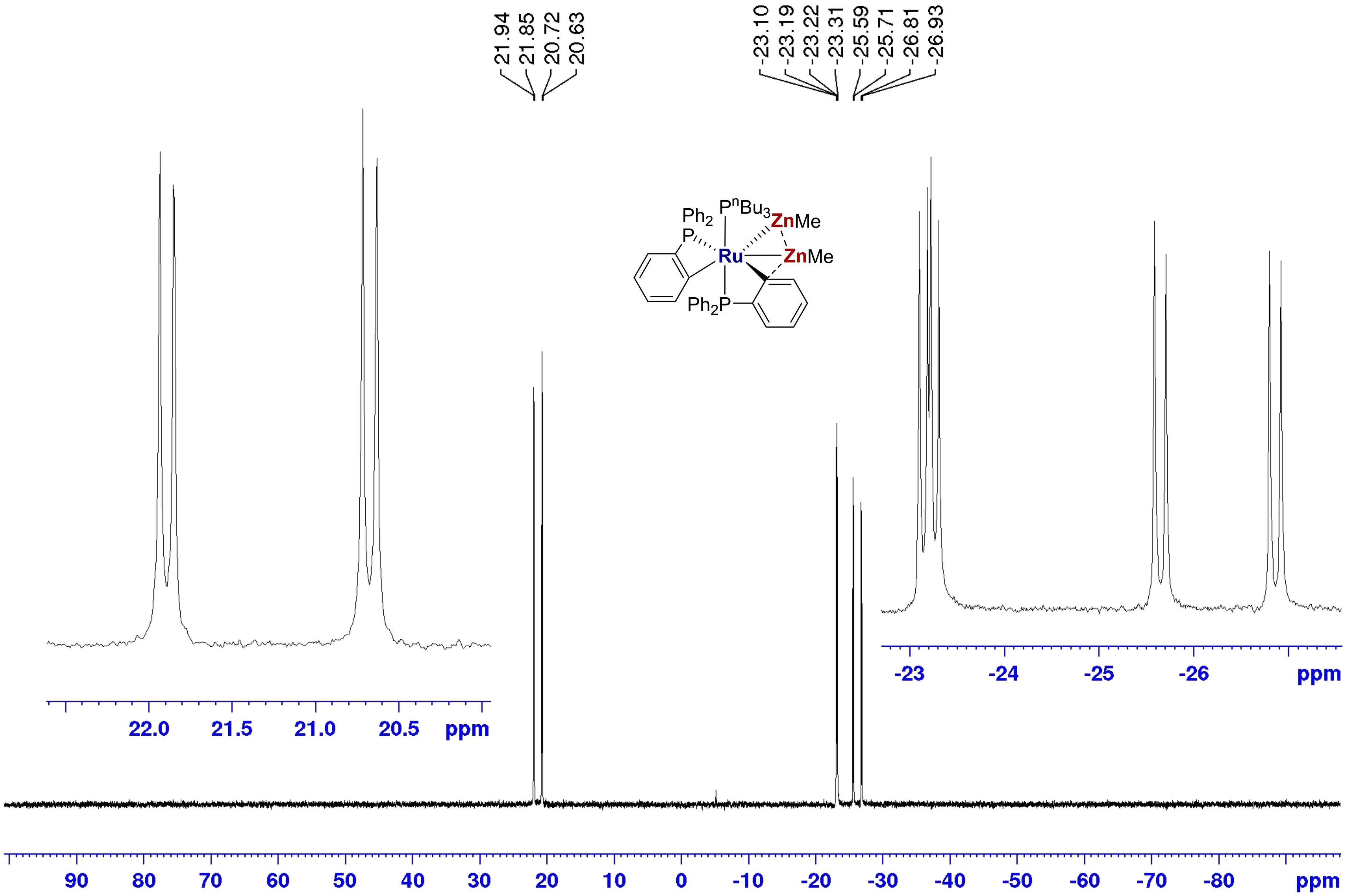

Figure S9. $^{31}P\{^{1}H\}$ NMR spectrum (202 MHz, C_6D_6, 298 K) of $[Ru(P^{n}Bu_3)(C_6H_4PPh_2)_2(ZnMe)_2]$ (**6**).

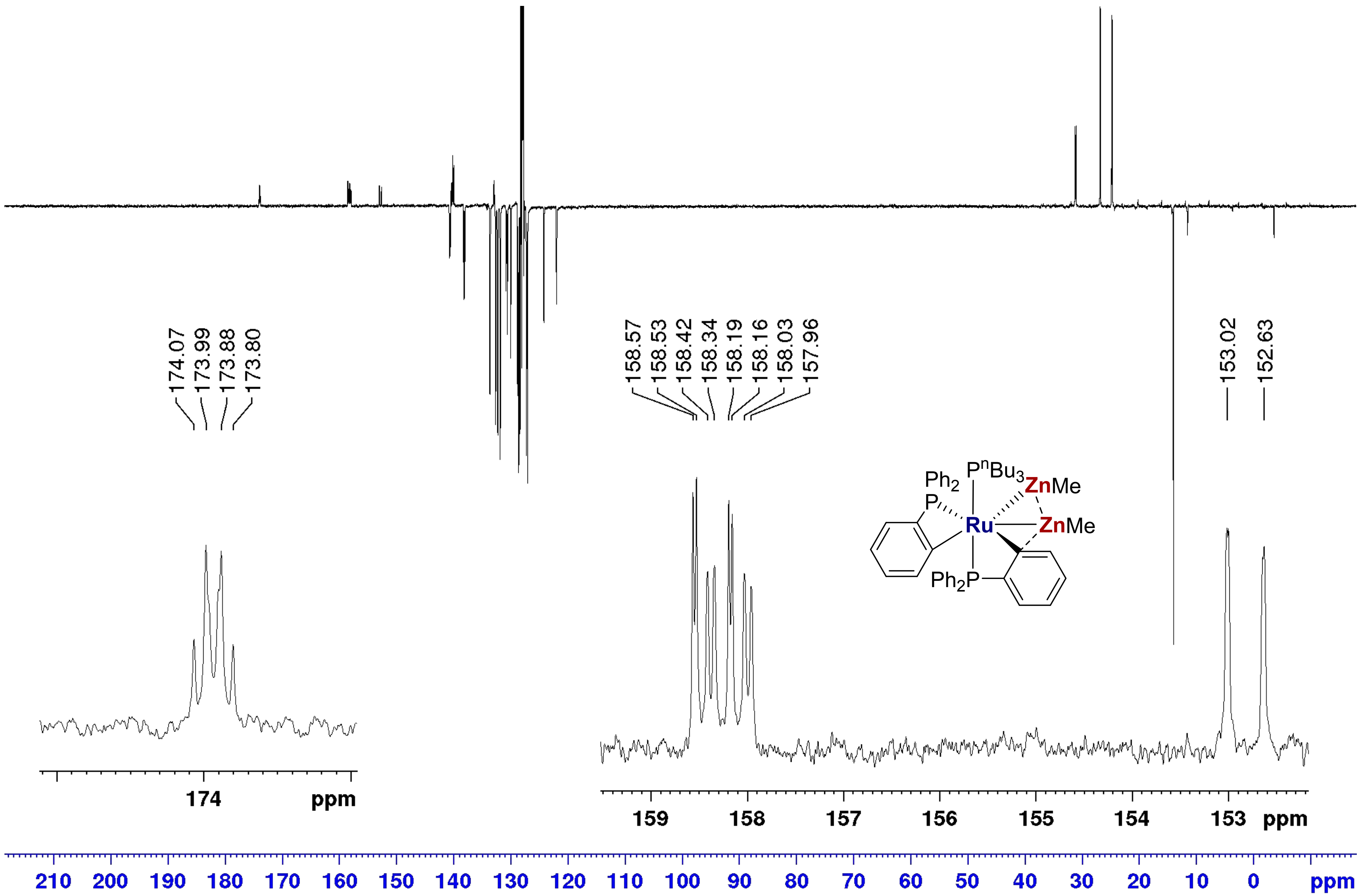

Figure S10.$^{13}C\{^1H\}$ (PENDANT) NMR spectrum (126 MHz, C_6D_6, 298 K) of [Ru(P^nBu_3)($C_6H_4PPh_2$)$_2$(ZnMe)$_2$] (**6**), with insets of high frequency resonances.

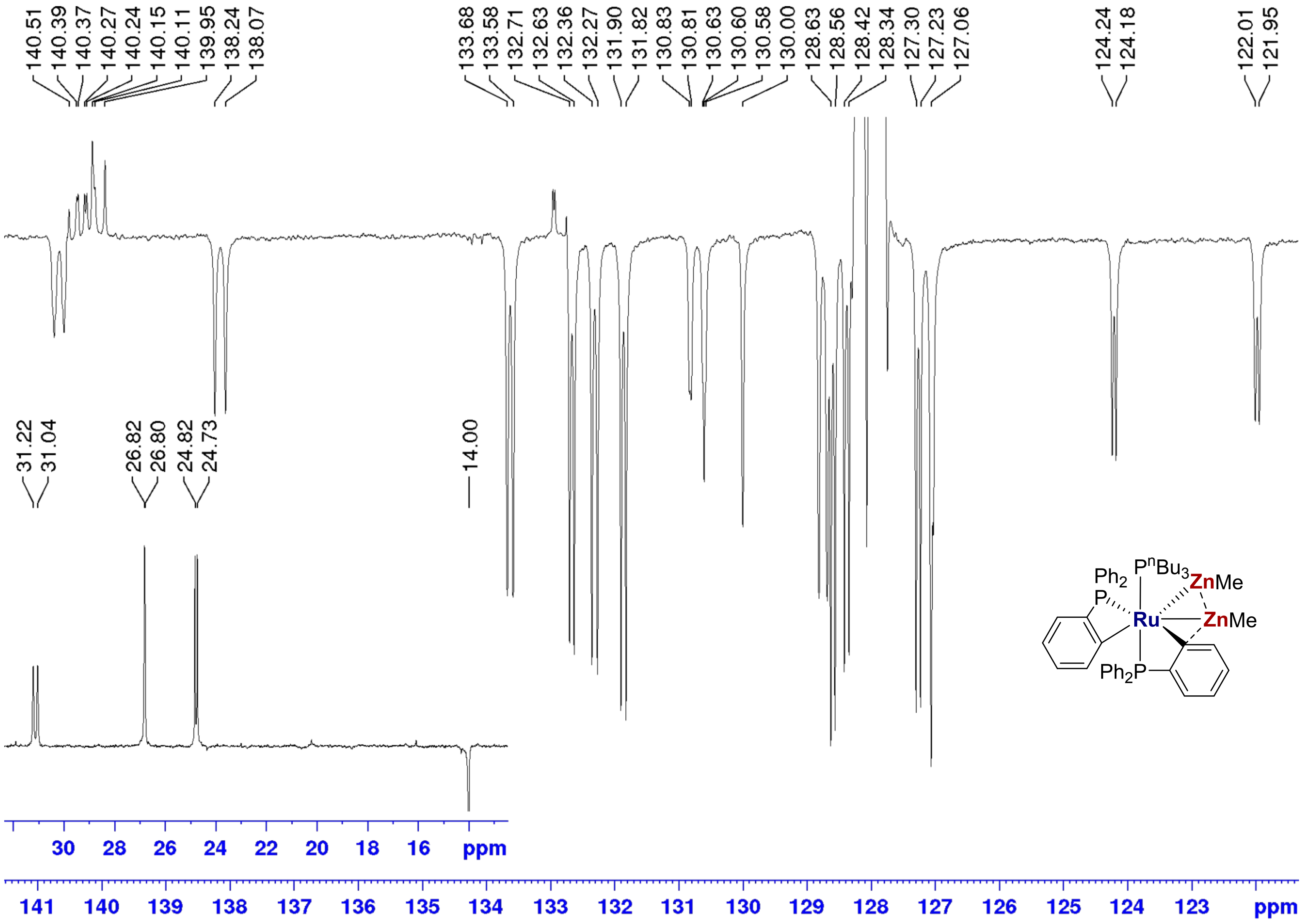

Figure S11. Aromatic and low frequency regions of the $^{13}C\{^1H\}$ (PENDANT) NMR spectrum (126 MHz, C_6D_6, 298 K) of $[Ru(P^nBu_3)(C_6H_4PPh_2)_2(ZnMe)_2]$ (**6**).

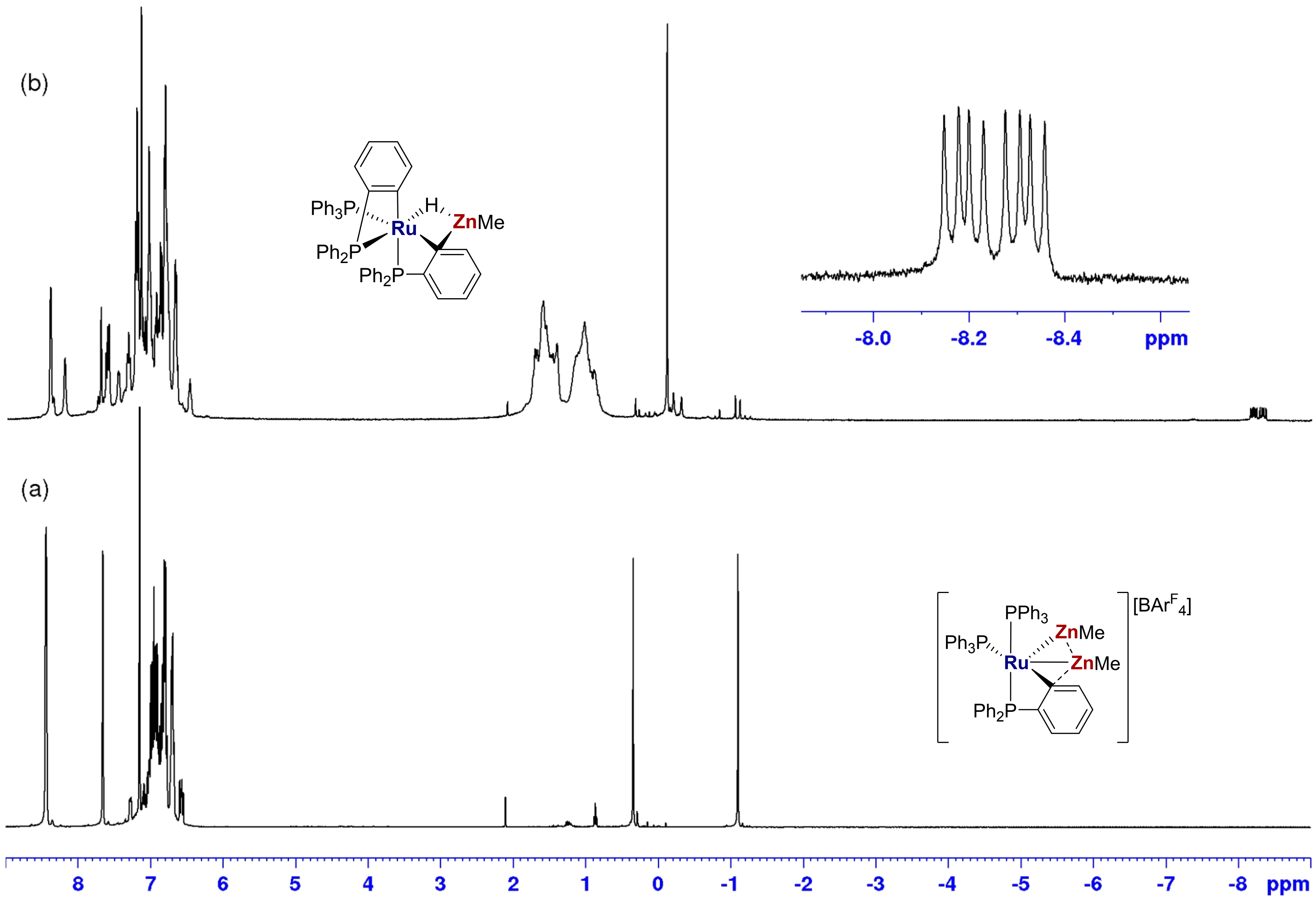

Figure S12. The ^{1}H NMR spectrum (400 MHz, C_6D_6, 298 K) of (a) isolated $[Ru(PPh_3)_2(C_6H_4PPh_2)(ZnMe)_2][BAr^F{}_4]$ (**3**) and (b) recorded 15 min after room temperature addition of PCy_3 (2 equiv) to **3** showing formation of $[Ru(PPh_3)(C_6H_4PPh_2)_2H(ZnMe)]$ (**1**). The diagnostic hydride signal of **1** is in the inset.

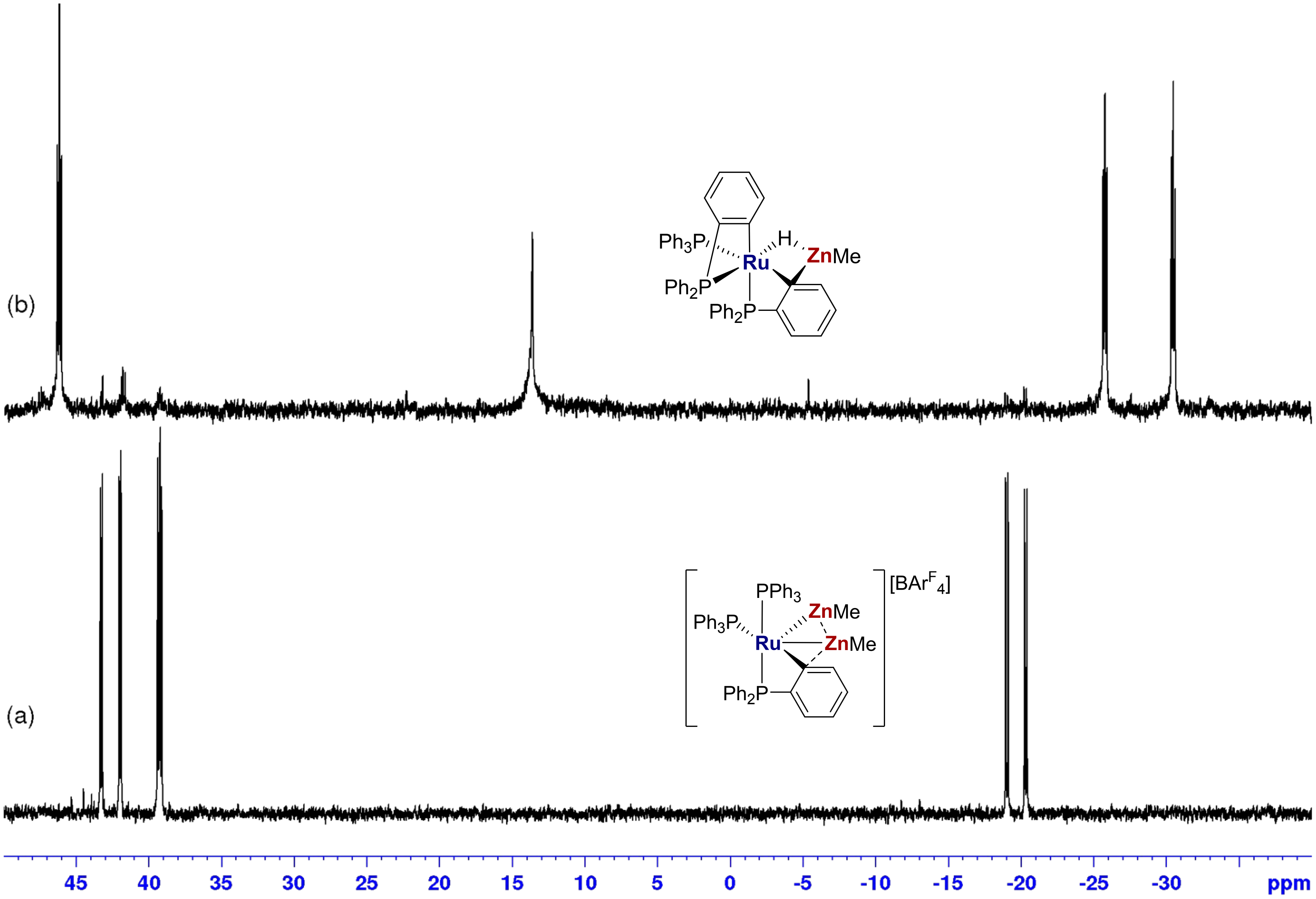

Figure S13. The $^{31}P\{^1H\}$ NMR spectrum (162 MHz, C_6D_6, 298 K) of (a) isolated $[Ru(PPh_3)_2(C_6H_4PPh_2)(ZnMe)_2][BAr^F{}_4]$ (**3**) and (b) 15 min after room temperature addition of PCy_3 (2 equiv) to **3** showing formation of $[Ru(PPh_3)(C_6H_4PPh_2)_2H(ZnMe)]$ (**1**).

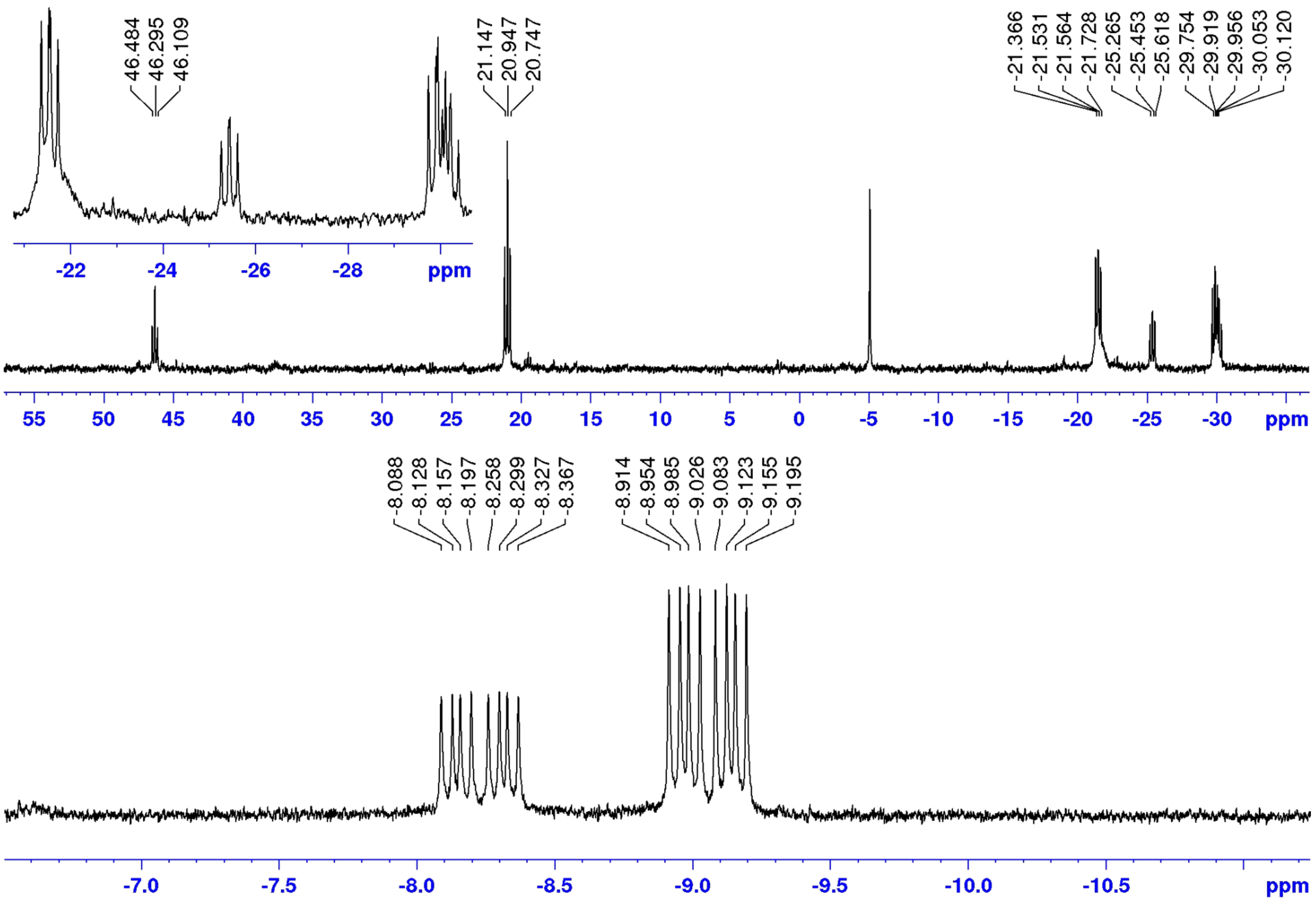

Figure S14. (Bottom) Room temperature (298 K) ^{1}H NMR (300 MHz) and (top) $^{31}P\{^{1}H\}$ NMR (121 MHz) recorded 15 min after addition of $P^{n}Bu_3$ (2 equiv) to a C_6D_6 solution of $[Ru(PPh_3)_2(C_6H_4PPh_2)(ZnMe)_2][BAr^{F}_4]$ (**3**).

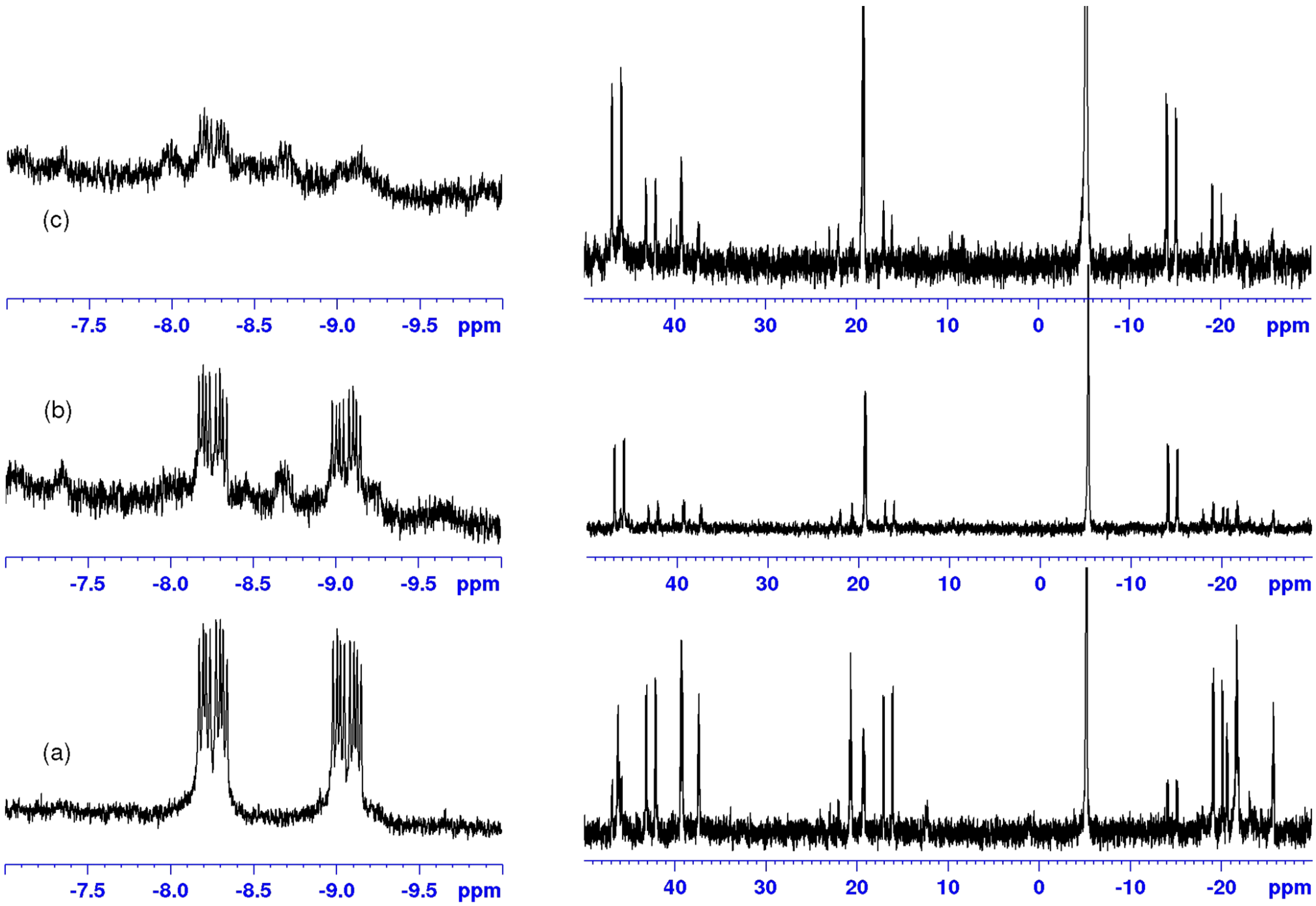

Figure S15. (Left) Hydride region of the ^{1}H NMR (400 MHz) and (right) $^{31}P\{^{1}H\}$ NMR (162 MHz) spectra (298 K) recorded (a) 15 min, (b) 6 h and (c) 25 h after addition of $P^{n}Bu_3$ (1 equiv) to a C_6D_6 solution of $[Ru(PPh_3)_2(C_6H_4PPh_2)(ZnMe)_2][BAr^{F}{}_4]$ (**3**).

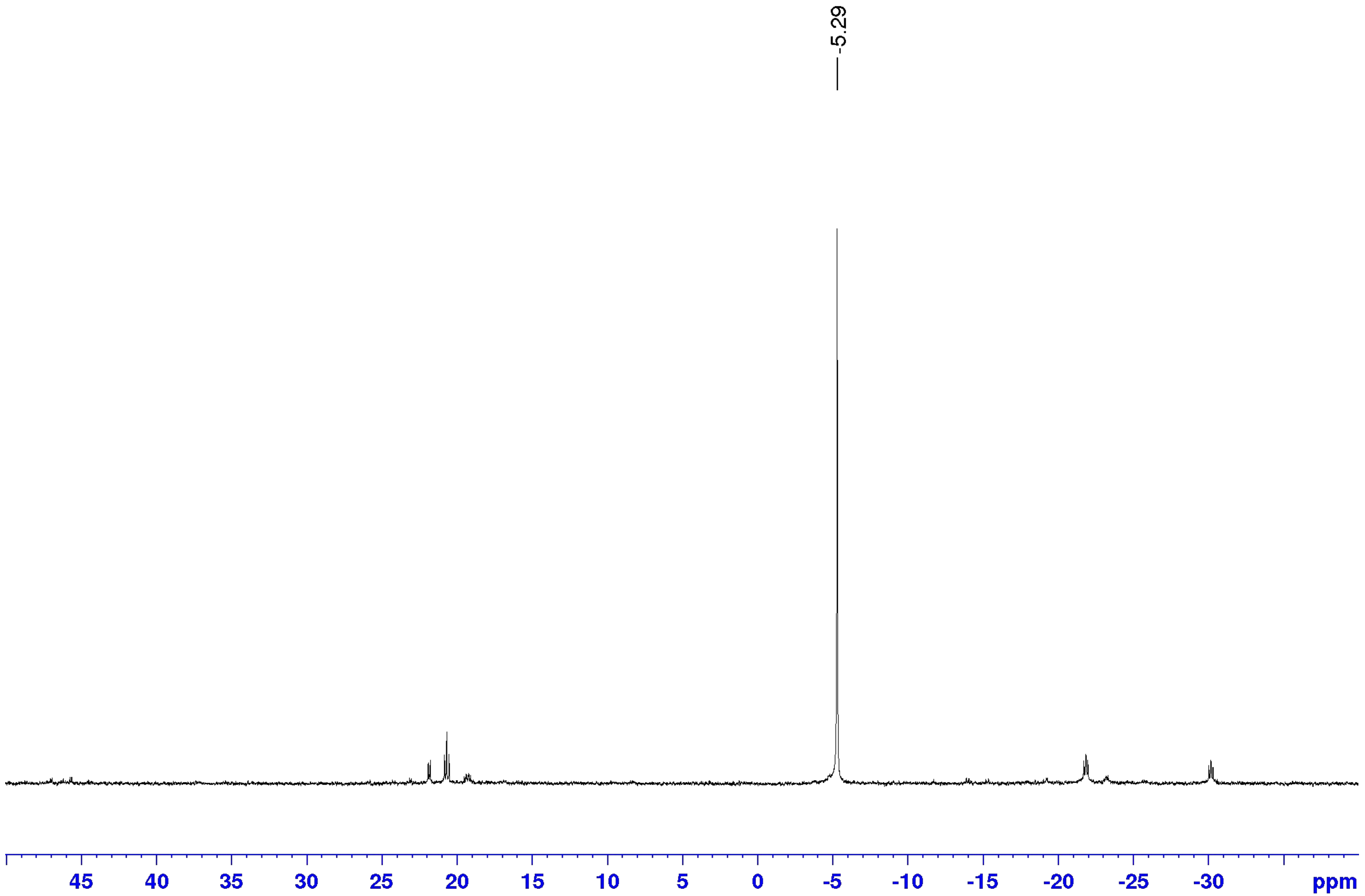

Figure S16. $^{31}P\{^1H\}$ NMR (121 MHz, 298 K, C_6D_6) spectrum recorded 20 h after treatment of $[Ru(PPh_3)_2(C_6H_4PPh_2)(ZnMe)_2][BAr^F{}_4]$ (**3**) with 2 equiv P^nBu_3.

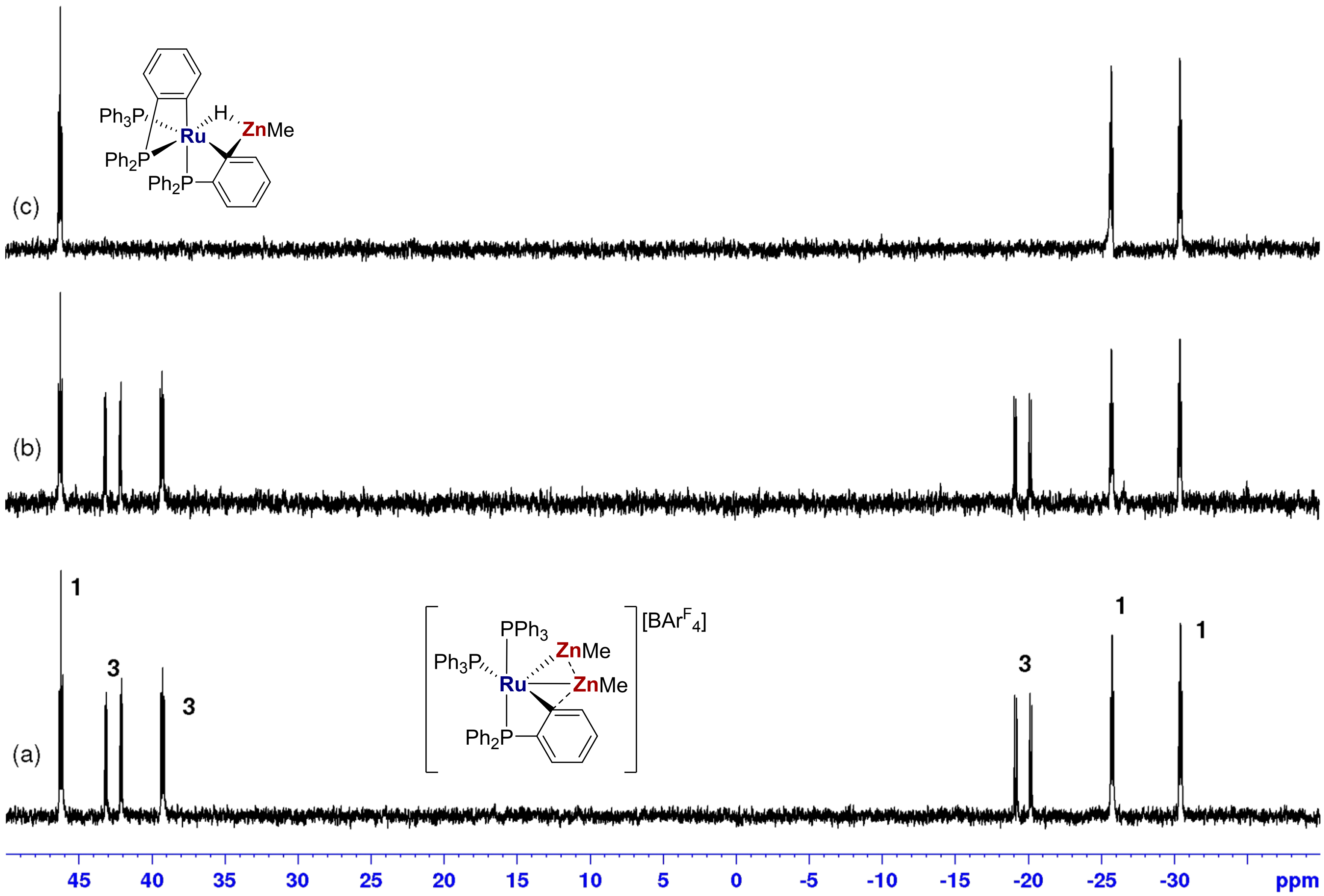

Figure S17. $^{31}P\{^1H\}$ NMR spectra (202 MHz, C_6D_6, 298 K) showing the reaction of $[Ru(PPh_3)_2(C_6H_4PPh_2)(ZnMe)_2][BAr^F_4]$ (**3**) with IMes at (a) 30 min and (b) 24 h after addition of 1 equiv IMes, and (c) 10 min after further addition of a second equivalent of the NHC.

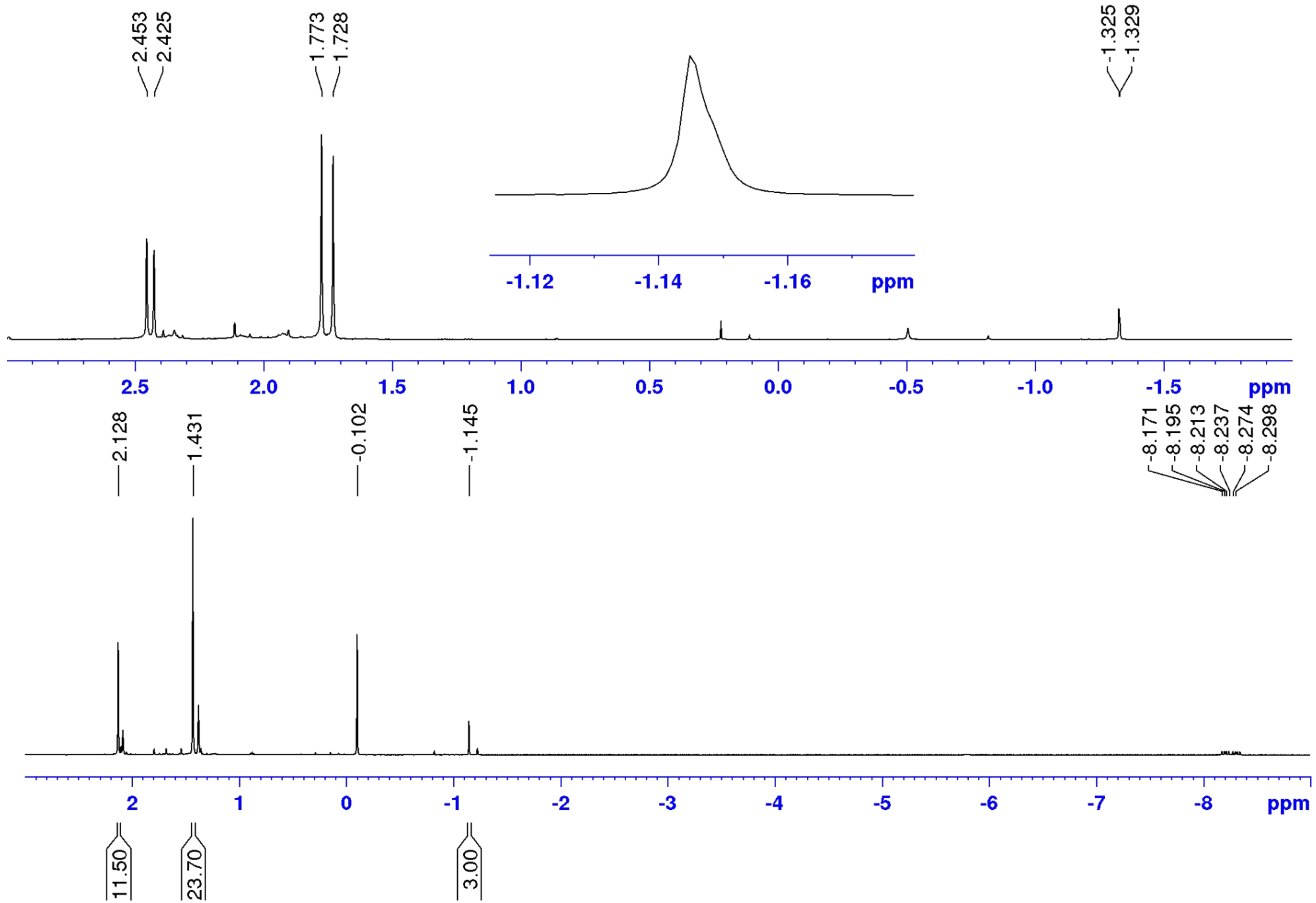

Figure S18. (Bottom) Low frequency of the ^{1}H NMR spectrum (400 MHz, C_6D_6, 298 K) of the reaction of [Ru(PPh$_3$)$_2$(C$_6$H$_4$PPh$_2$)(ZnMe)$_2$][BAr$^F{}_4$] (**3**) with 2 equiv IMes and (top) methyl region of after removal of the C_6D_6 and dissolution of the residue in CD_2Cl_2 to confirm formation of [(IMes)$_2$ZnMe]$^+$.[2]

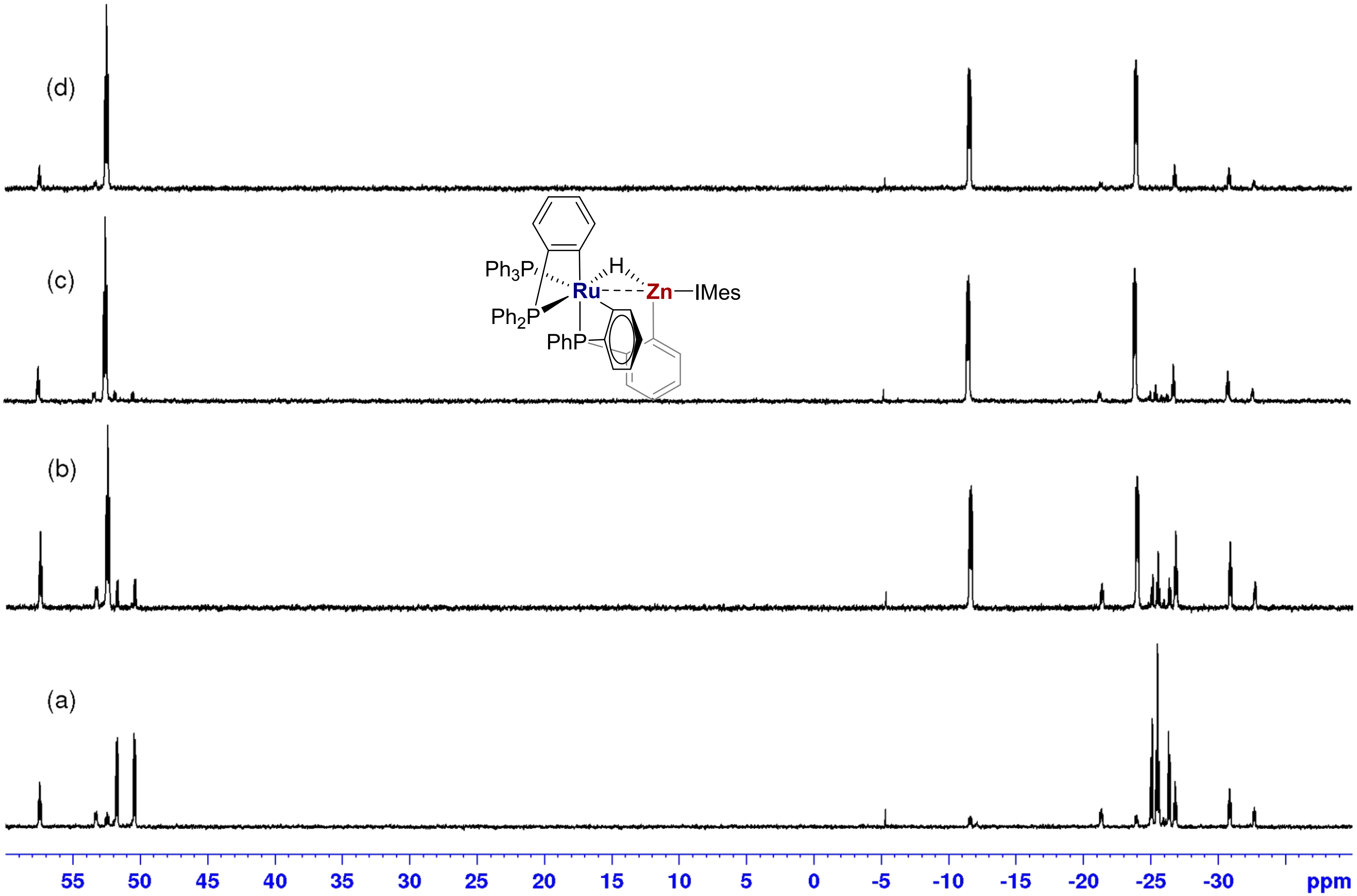

Figure S19. $^{31}P\{^{1}H\}$ NMR spectra (202 MHz, C_6D_6, 298 K) of the room temperature reaction of [Ru(PPh$_3$)(C$_6$H$_4$PPh$_2$)$_2$(ZnMe)$_2$] (**2**) with IMes (2 equiv) to give [Ru(PPh$_3$)(C$_6$H$_4$PPh$_2$)(PPh(C$_6$H$_4$)$_2$Zn(IMes))H] (**7**) after (a) 15 min, (b) 1 h 15 min, (c) 2 h 45 min and (d) 24 h. See Figures S20 and S21 for more details.

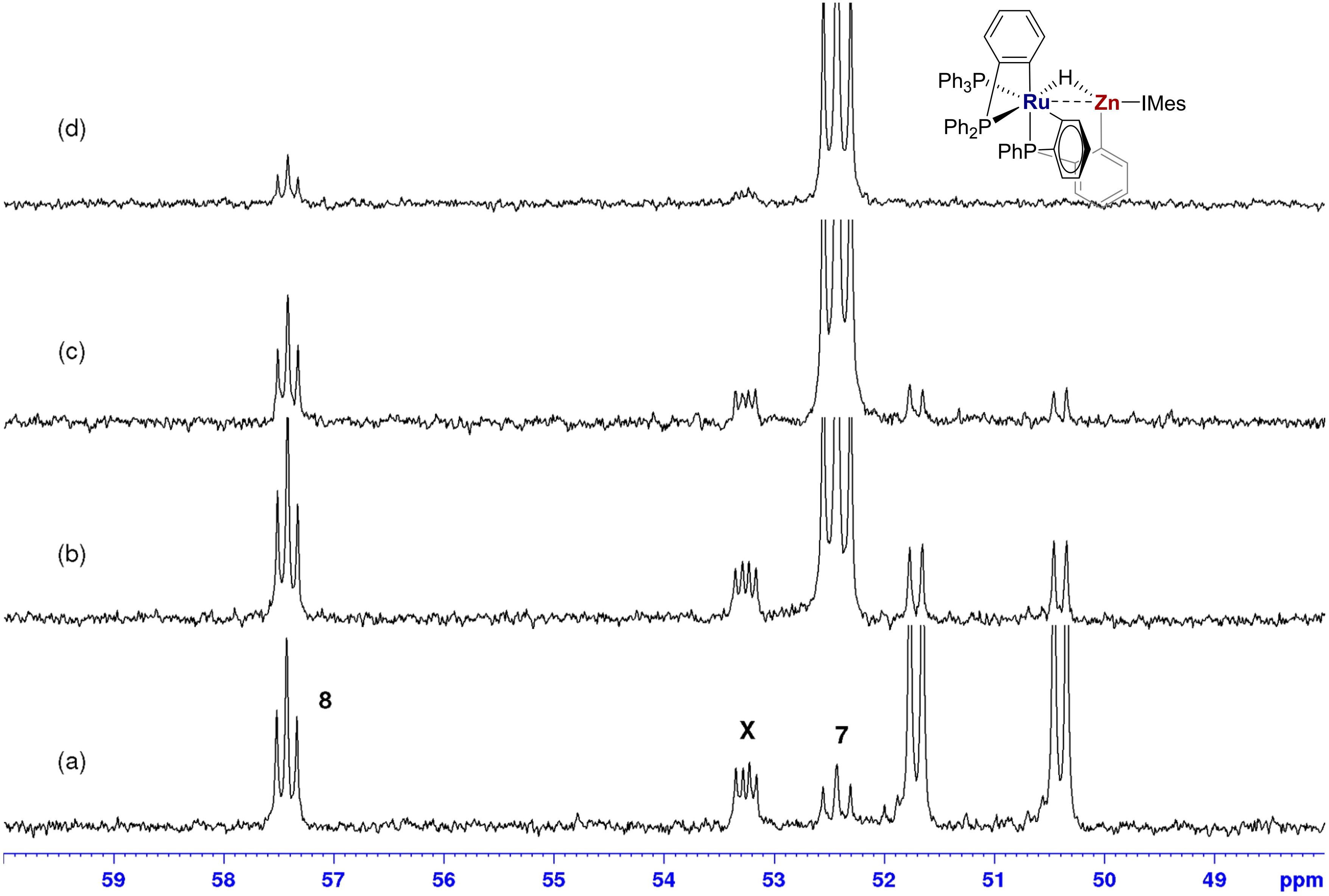

Figure S20. High frequency region of $^{31}P\{^{1}H\}$ NMR spectra from Figure S19 again shown after (a) 15 min, (b) 1 h 15 min, (c) 2 h 45 min and (d) 24 h highlighting the appearance of signals for [Ru(PPh$_3$)(C$_6$H$_4$PPh$_2$)(PPh(C$_6$H$_4$)$_2$Zn(IMes))H] (**7**) and minor isomer **8**, as well as unknown intermediate **X**.

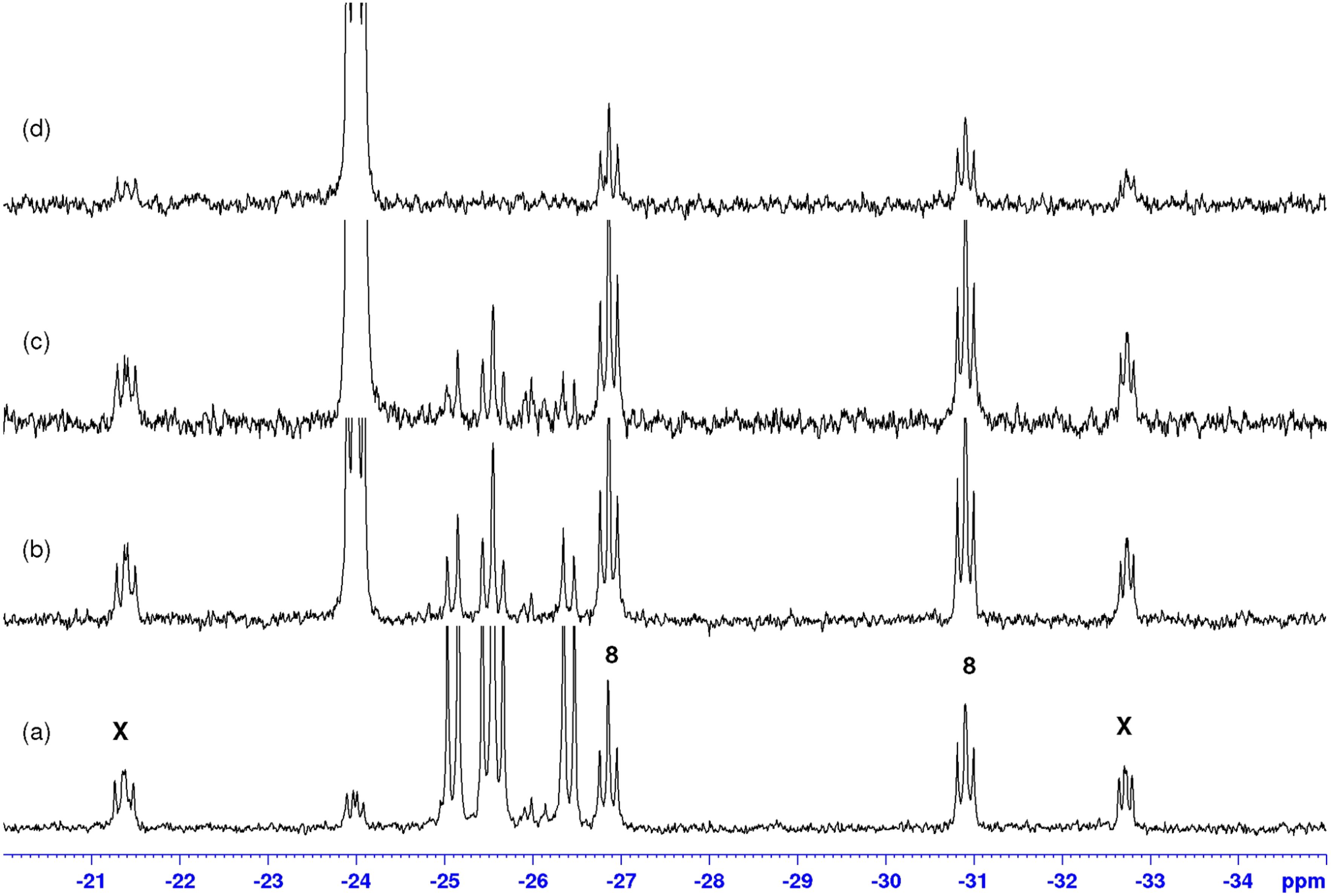

Figure S21. Low frequency region of $^{31}P\{^{1}H\}$ NMR spectra from Figure S19 again shown after (a) 15 min, (b) 1 h 15 min, (c) 2 h 45 min and (d) 24 h highlighting the appearance of signals for **8**, and the unknown intermediate **X**.

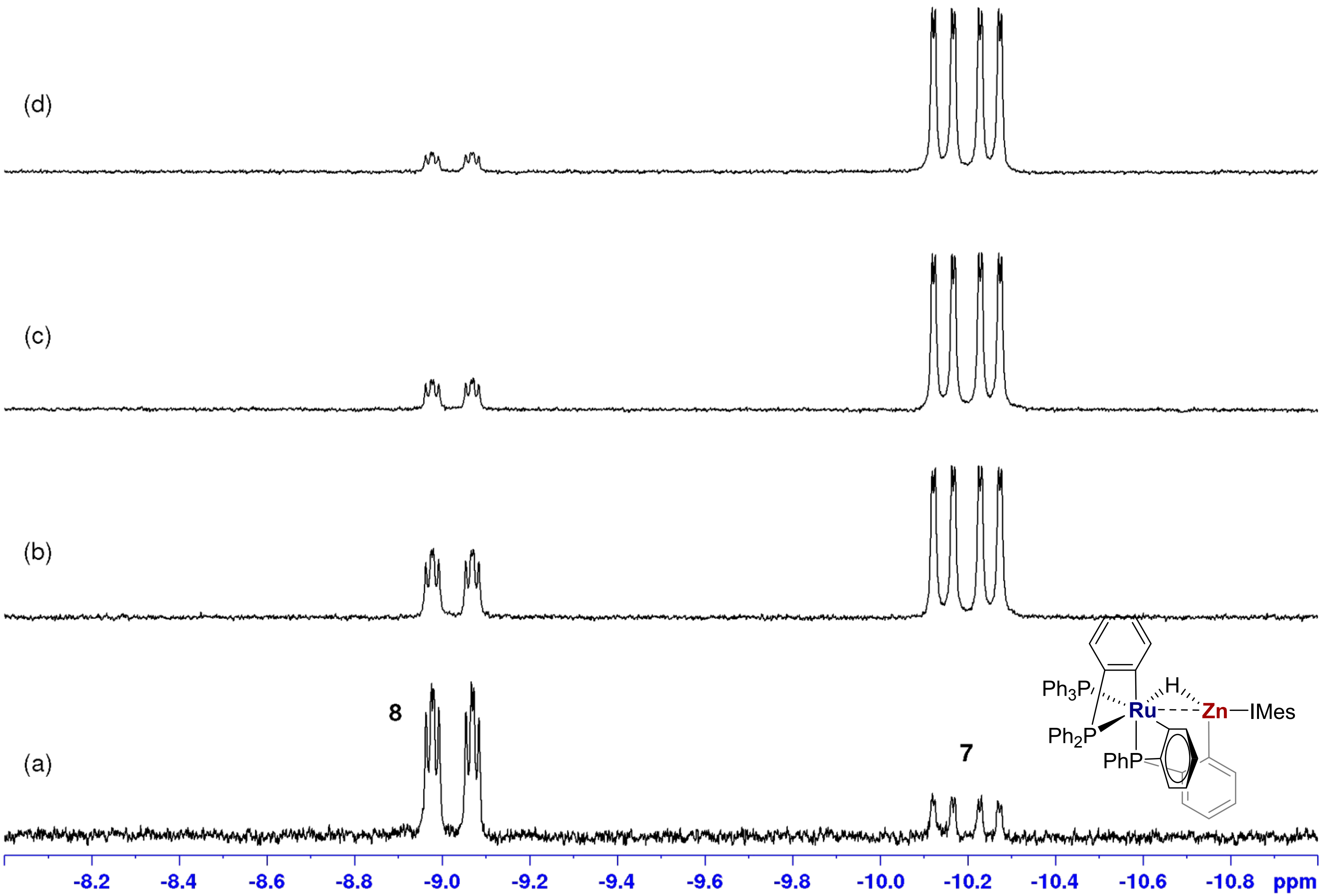

Figure S22. Hydride region of ^{1}H NMR spectra (500 MHz, C_6D_6, 298 K) of the room temperature reaction of [Ru(PPh$_3$)(C$_6$H$_4$PPh$_2$)$_2$(ZnMe)$_2$] (**2**) with 2 equiv IMes after (a) 15 min, (b) 1 h 15 min, (c) 2 h 45 min and (d) 24 h showing the variation of [Ru(PPh$_3$)(C$_6$H$_4$PPh$_2$)(PPh(C$_6$H$_4$)$_2$Zn(IMes))H] (**7**) and **8**.

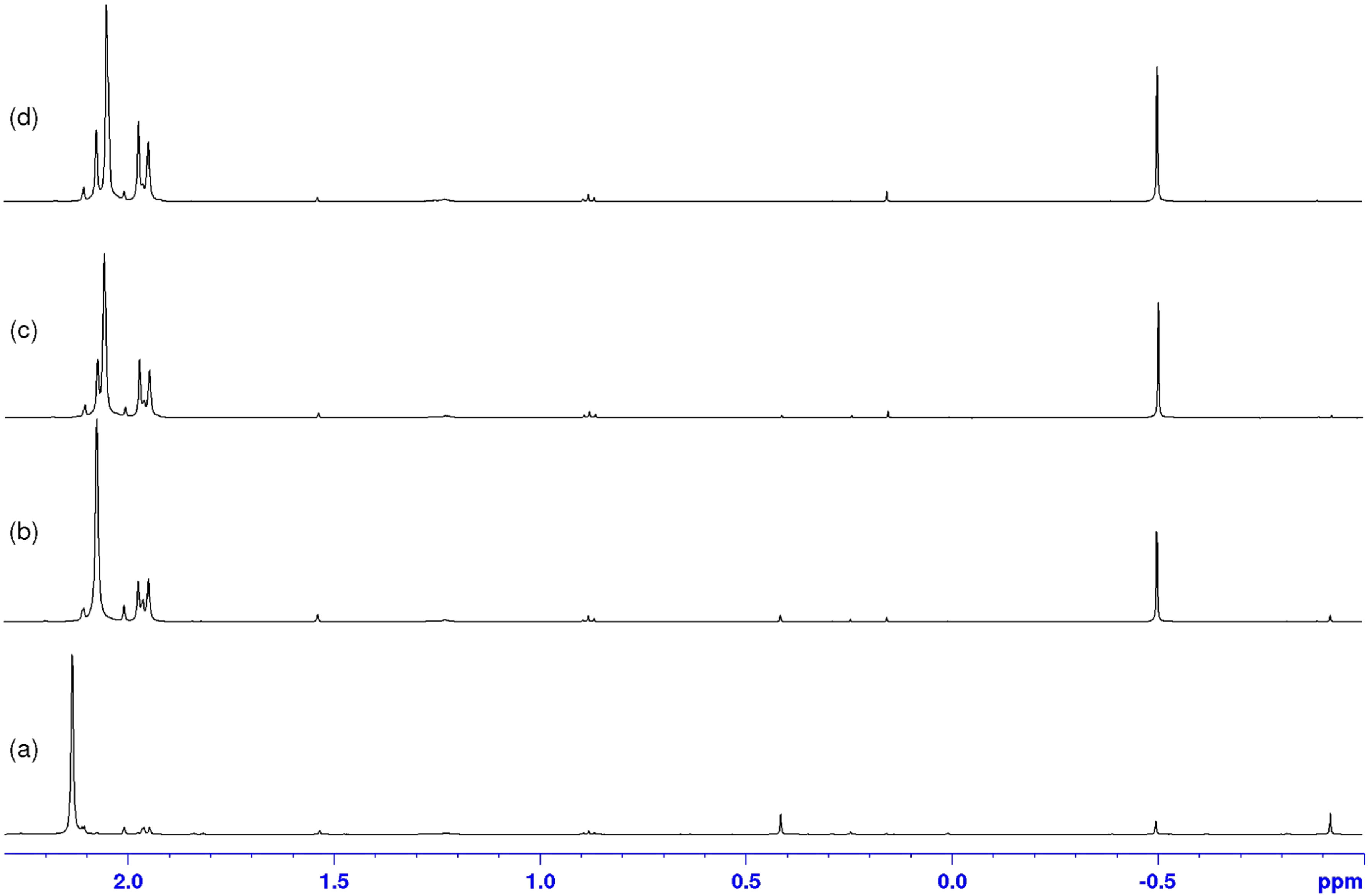

Figure S23. ^{1}H NMR spectra (500 MHz, C_6D_6, 298 K) of the room temperature reaction of [Ru(PPh$_3$)(C$_6$H$_4$PPh$_2$)$_2$(ZnMe)$_2$] (**2**) with IMes (2 equiv) after (a) 15 min, (b) 1 h 15 min, (c) 2 h 45 min and (d) 24 h showing the formation of (IMes)ZnMe$_2$ at ca. δ -0.5.[3]

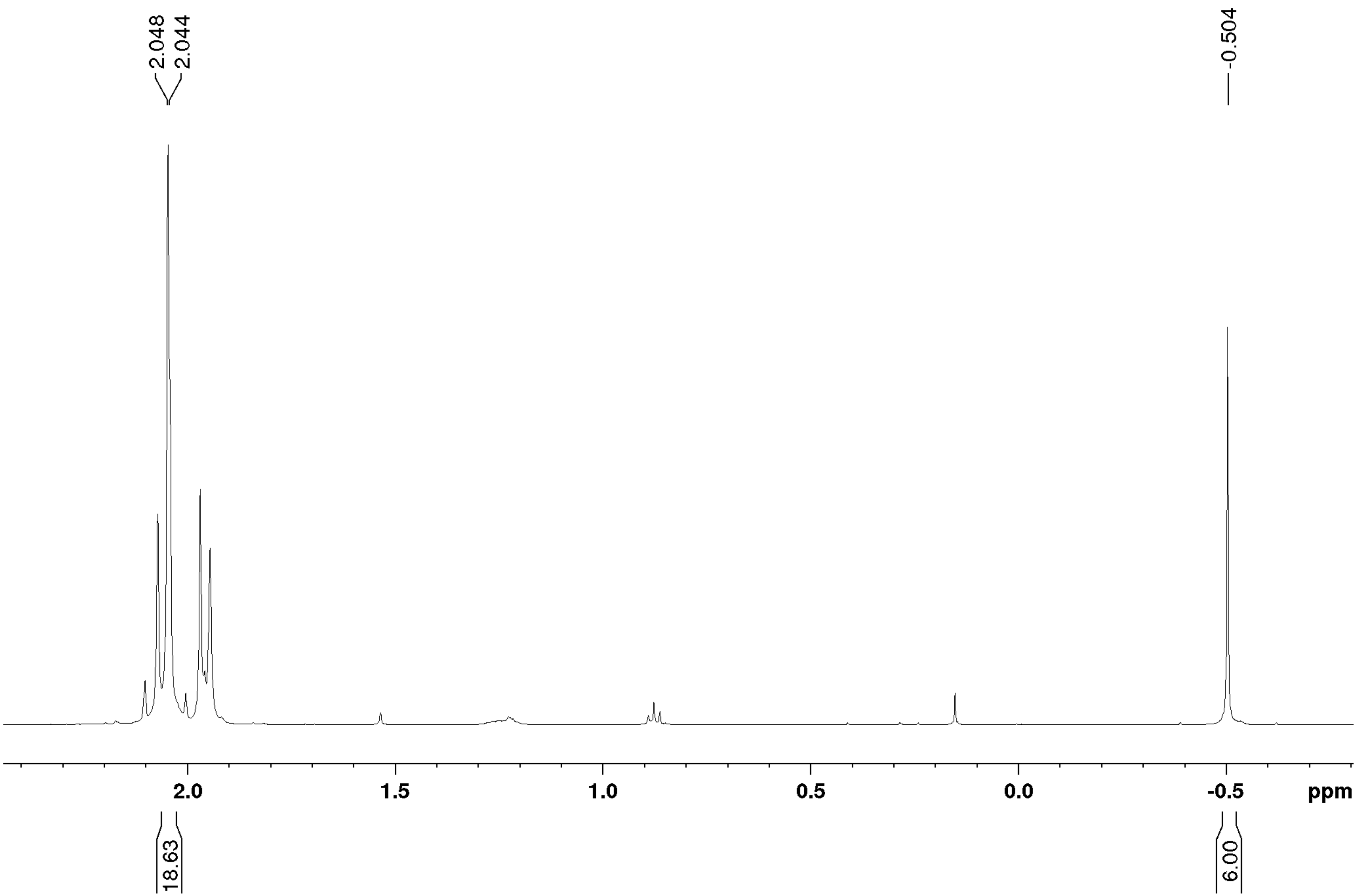

Figure S24. ^{1}H NMR spectrum (500 MHz, C_6D_6, 298 K) recorded 24 h into the room temperature reaction of $[Ru(PPh_3)(C_6H_4PPh_2)_2(ZnMe)_2]$ (**2**) with IMes (2 equiv) highlighting the methyl resonances assigned to (IMes)$ZnMe_2$.[3]

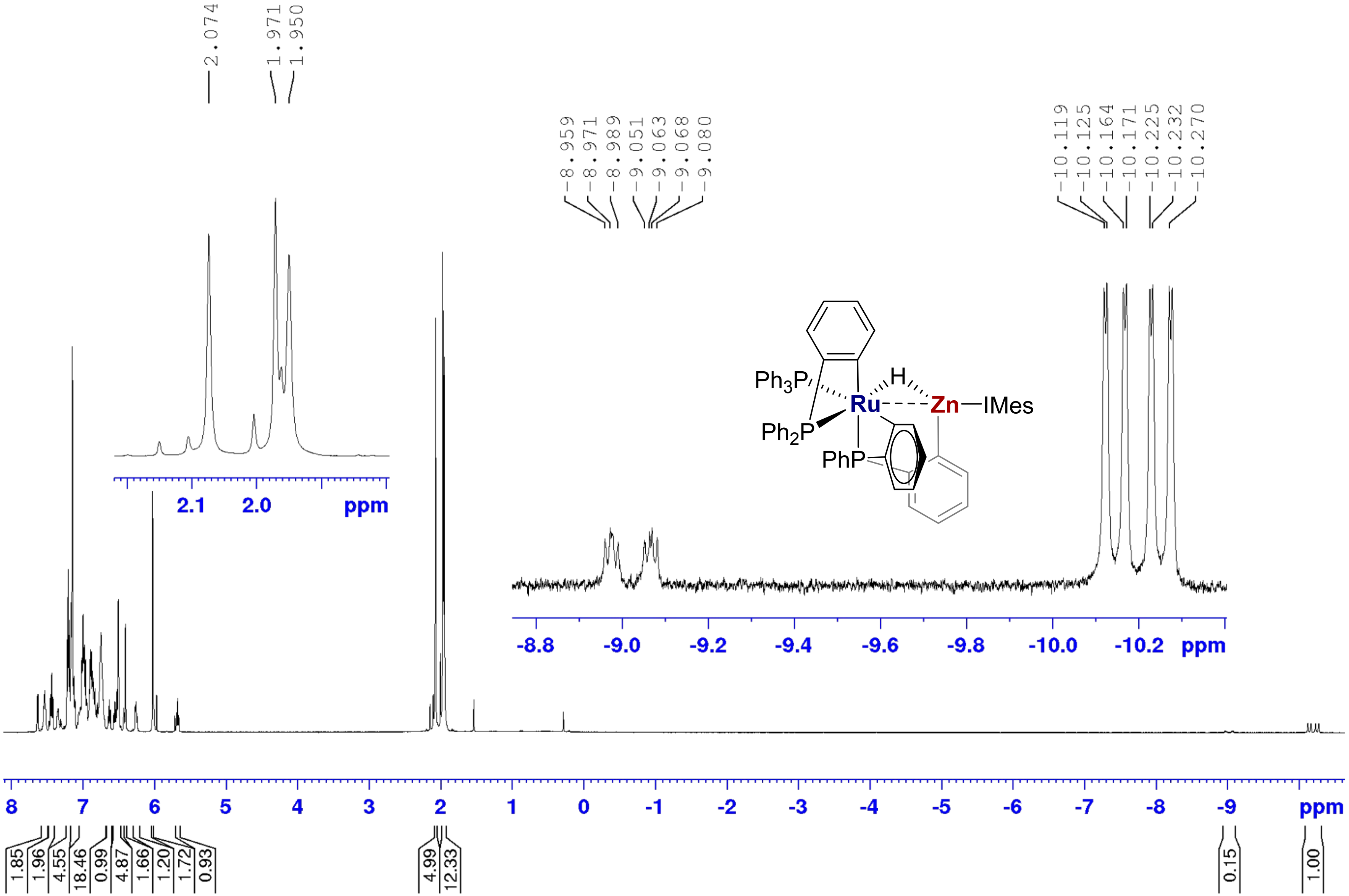

Figure S25. ^{1}H NMR spectrum (500 MHz, C_6D_6, 298 K) of [Ru(PPh_3)($C_6H_4PPh_2$)(PPh(C_6H_4)$_2$Zn(IMes))H] (**7**), with inset of Ru-*H*-Zn (highlighting the presence of **7** and minor isomer **8**) and IMes regions. NB aromatic signals fail to integrate due to partial overlap with residual C_6D_5H.

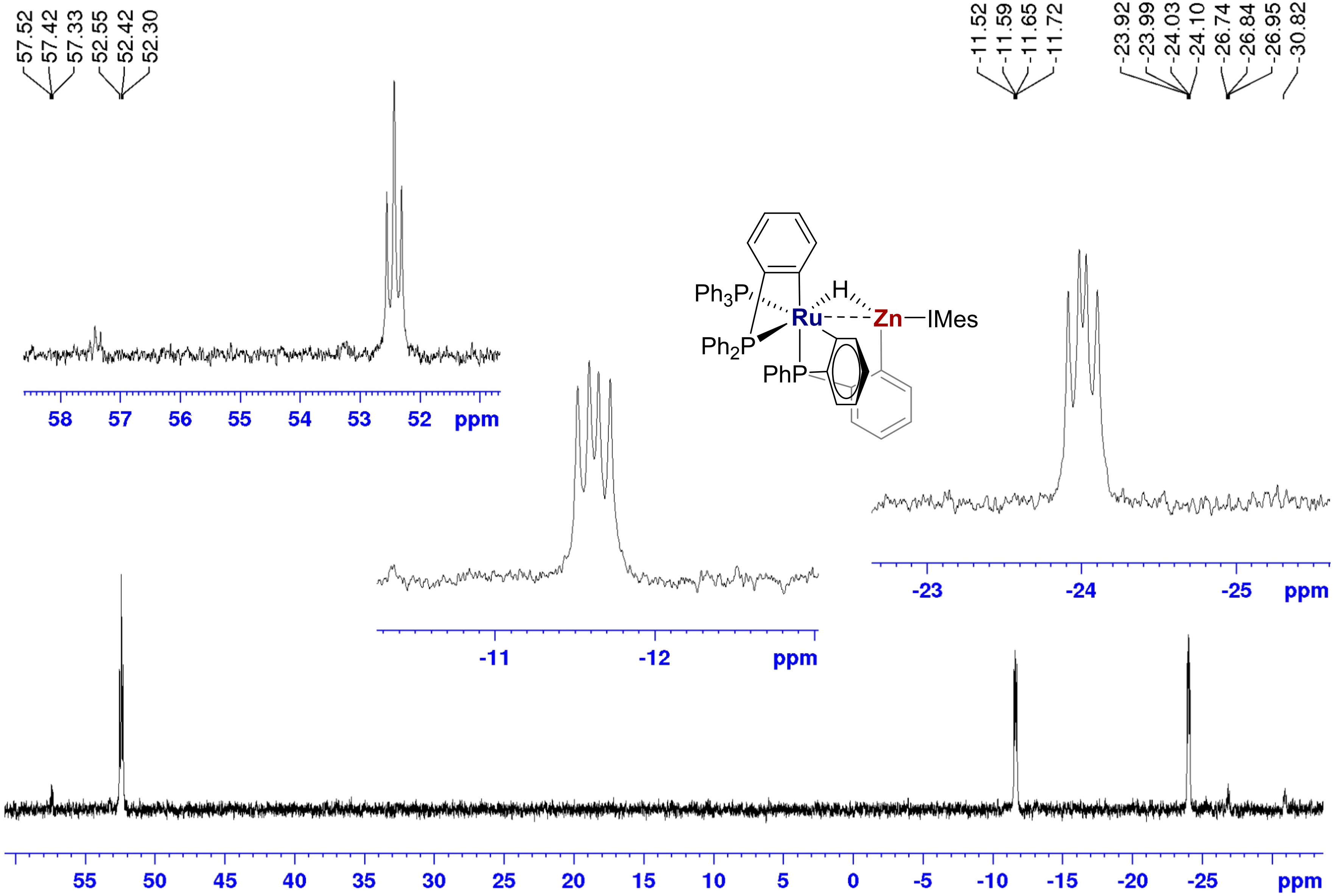

Figure S26. $^{31}P\{^1H\}$ NMR spectrum (202 MHz, C_6D_6, 298 K) of [Ru(PPh_3)($C_6H_4PPh_2$)(PPh(C_6H_4)$_2$Zn(IMes))H] (**7**), showing both **7** and minor isomer **8**.

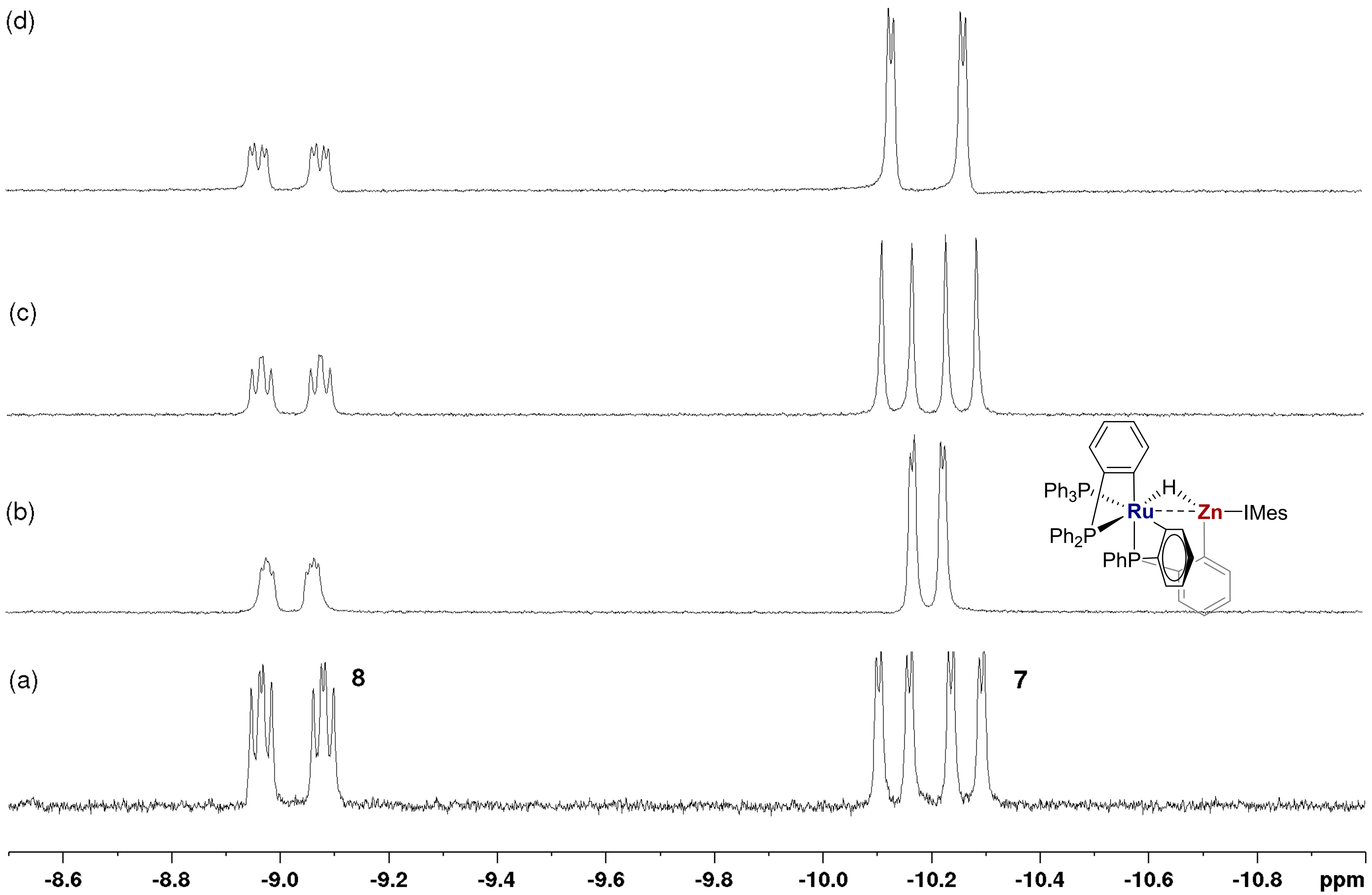

Figure S27. Selective ^{31}P decoupling at (b) δ 52, (c) δ -12 and (d) δ -24 showing the impact on the hydride signal (400 MHz, C_6D_6, 298 K) of **7** (a).

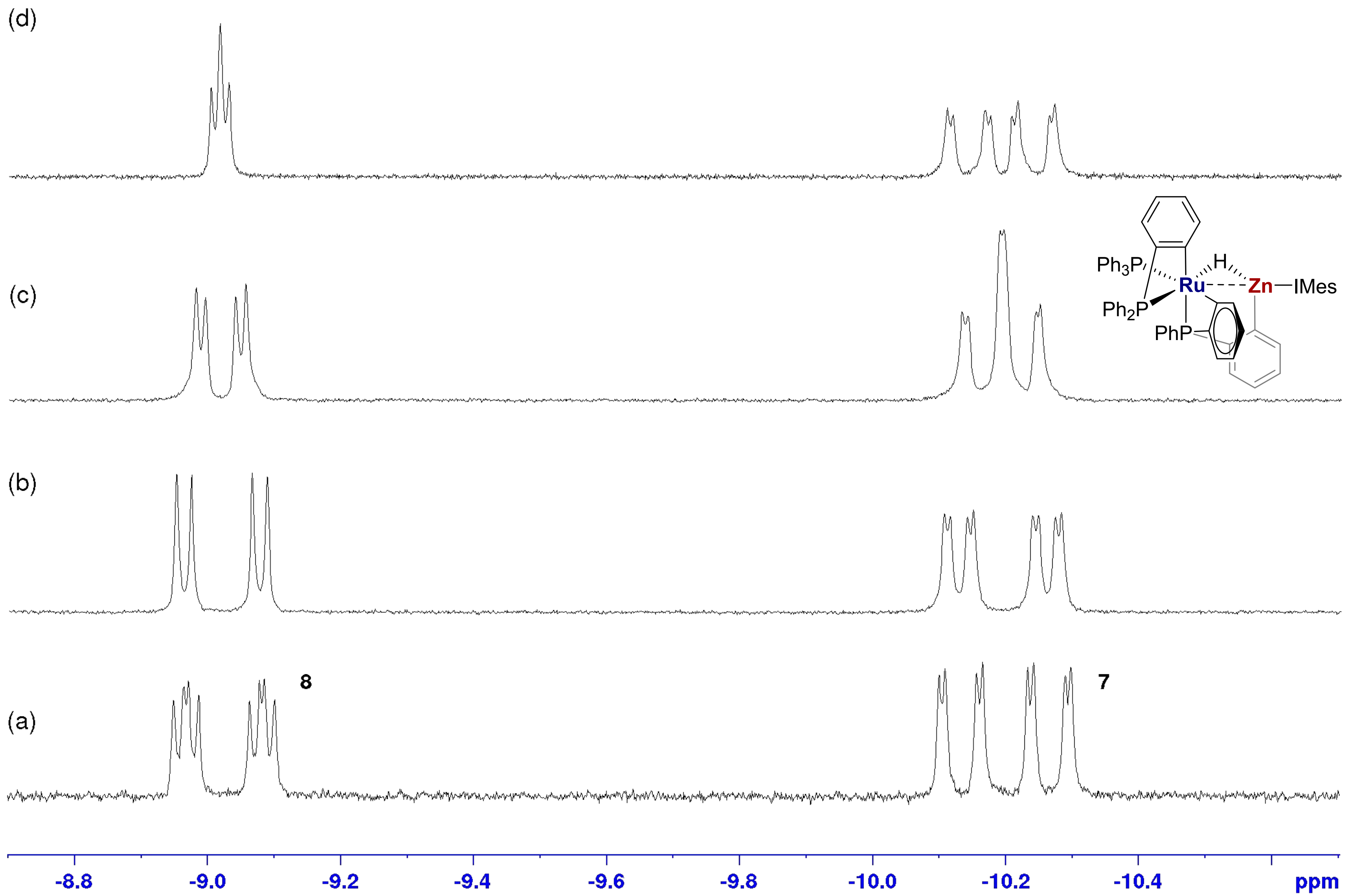

Figure S28. Selective ^{31}P decoupling at (b) δ 57, (c) δ -27 and (d) δ -31 showing the impact on the hydride signal (400 MHz, C_6D_6, 298 K) of **8** (a).

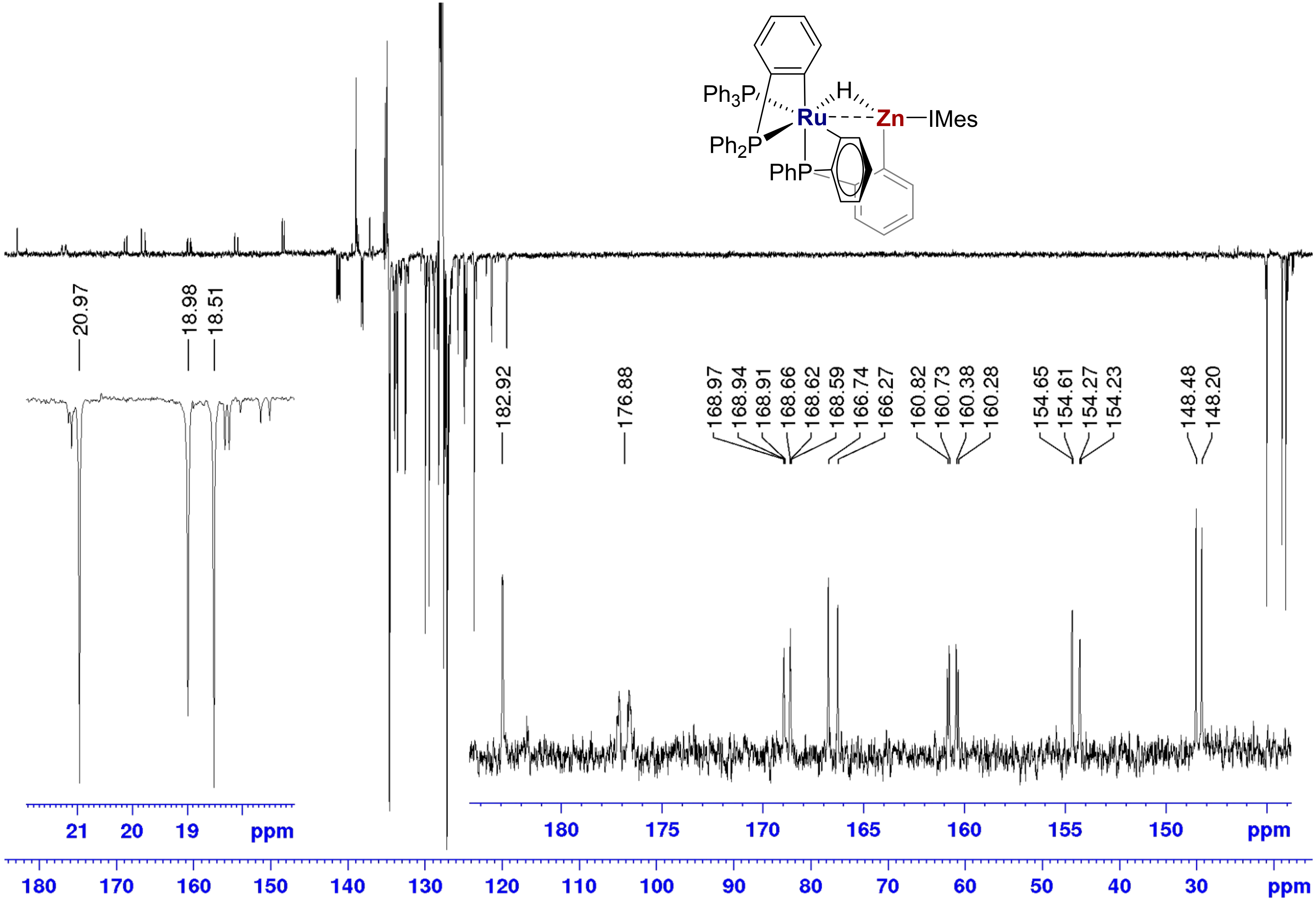

Figure S29. $^{13}C\{^1H\}$ (PENDANT) NMR spectrum (126 MHz, C_6D_6, 298 K) of [Ru(PPh_3)($C_6H_4PPh_2$)(PPh(C_6H_4)$_2$Zn(IMes))H] (**7**), with insets of high frequency resonances.

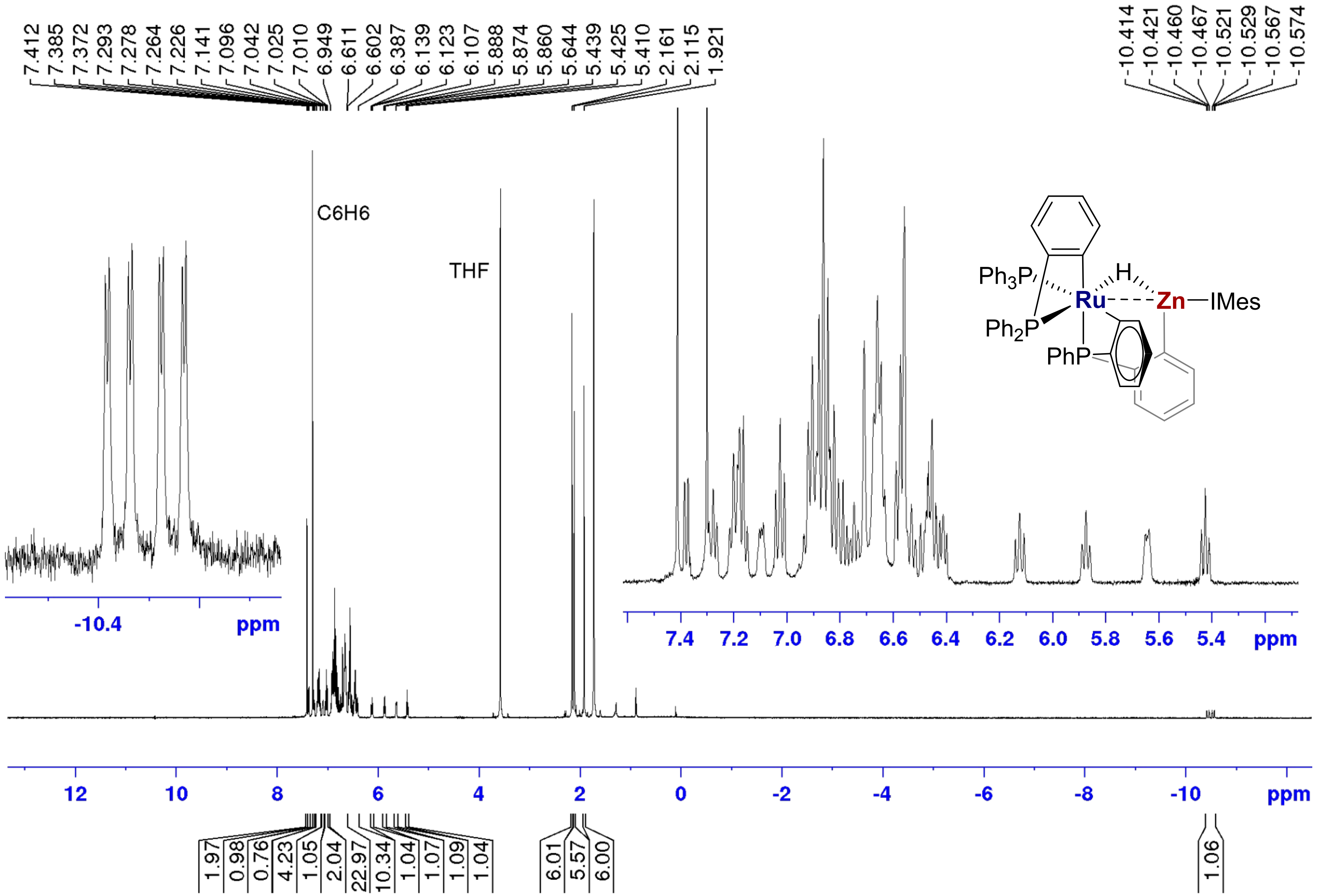

Figure S30. ^{1}H NMR spectrum (500 MHz, THF-d_8, 298 K) of [Ru(PPh$_3$)(C$_6$H$_4$PPh$_2$)(PPh(C$_6$H$_4$)$_2$Zn(IMes))H] (**7**), with inset of high frequency aryl and low frequency Ru-*H*-Zn regions.

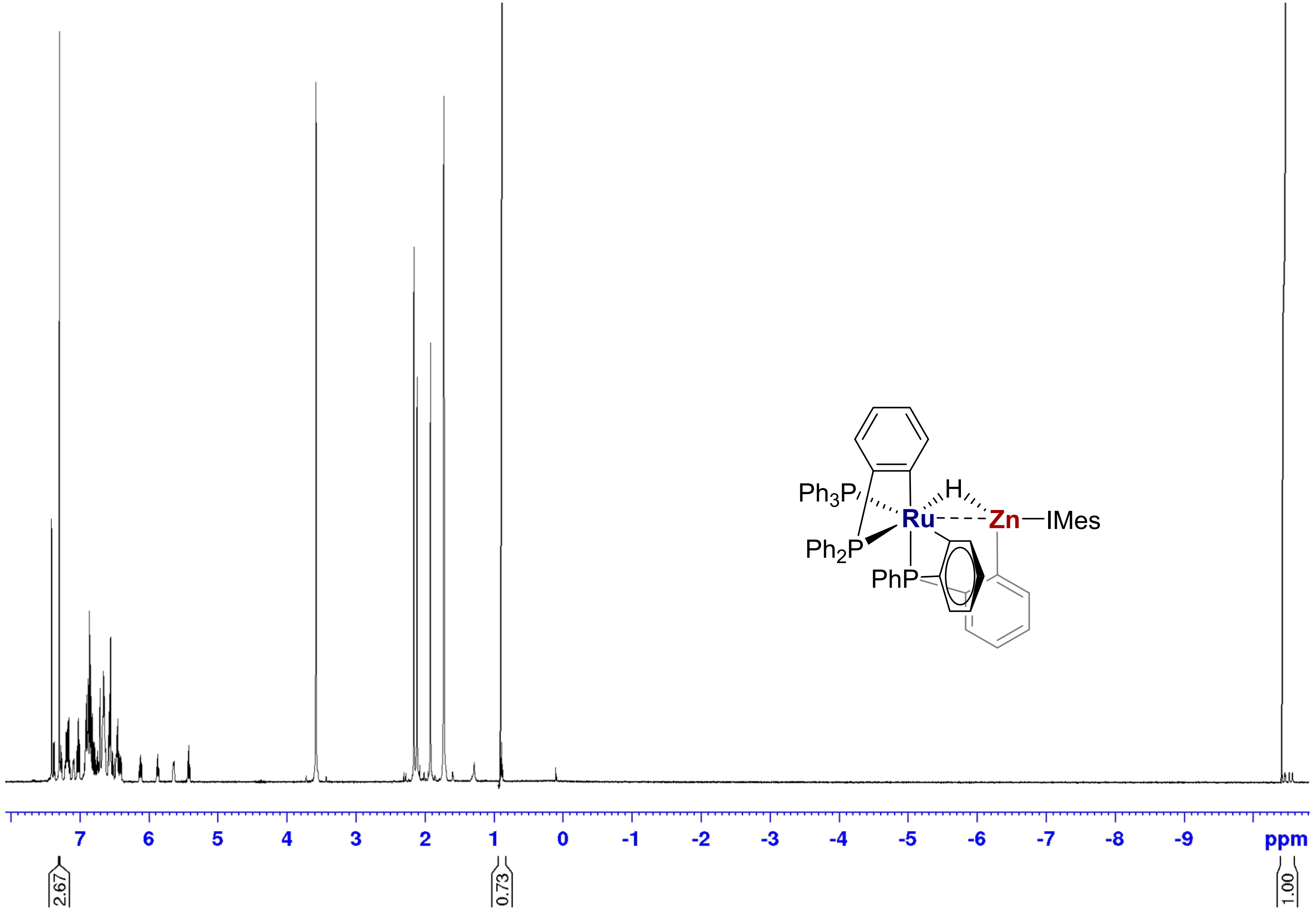

Figure S31. ^{1}H NMR spectrum (500 MHz, THF-d_8, 298 K) of crystalline [Ru(PPh$_3$)(C$_6$H$_4$PPh$_2$)(PPh(C$_6$H$_4$)$_2$Zn(IMes))H] (**7**) used in microanalysis, showing integrations of hexane and benzene.

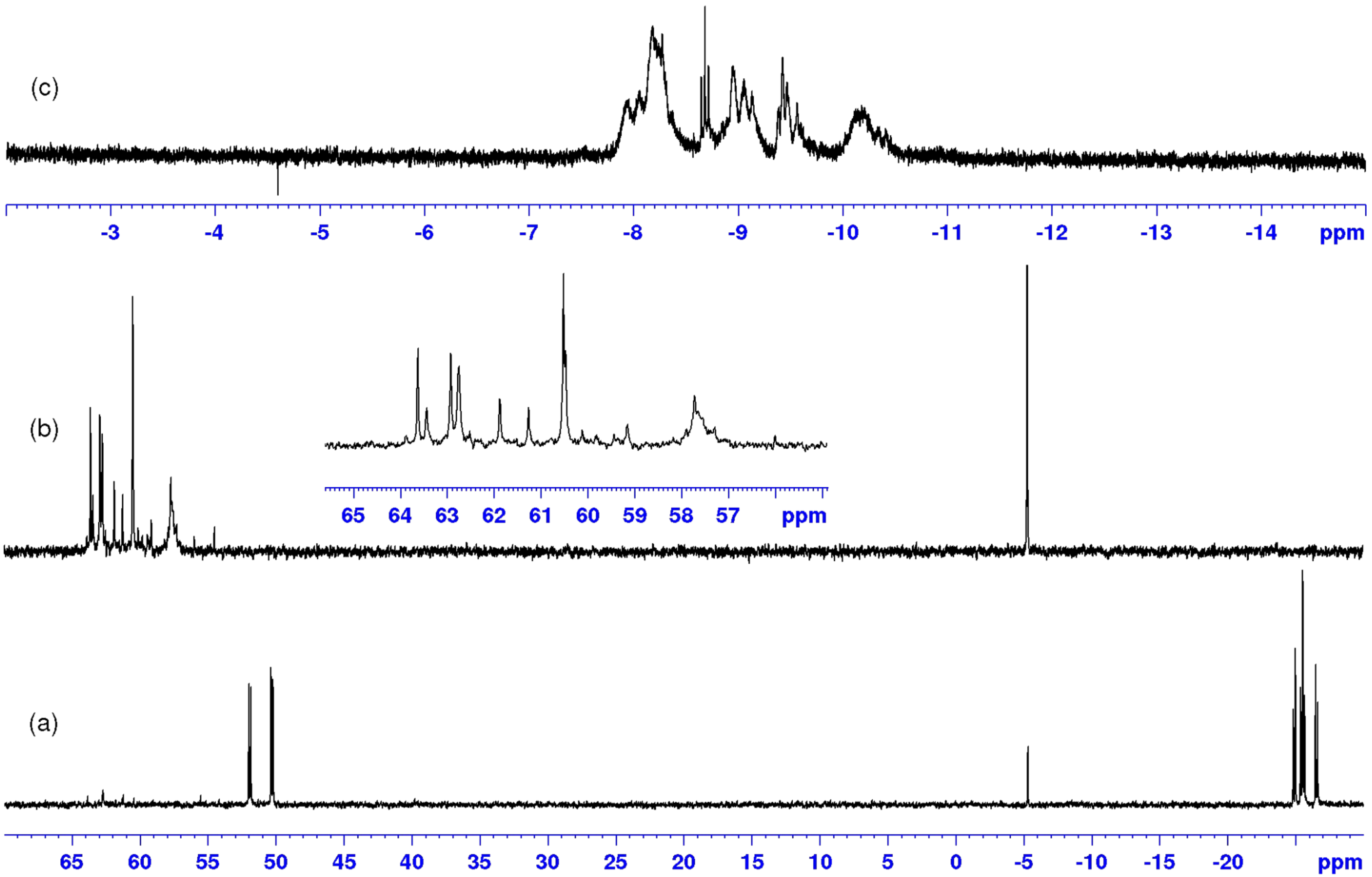

Figure S32. $^{31}P\{^{1}H\}$ NMR spectra (162 MHz, C_6D_6, 298 K) recorded after heating [Ru(PPh$_3$)(C$_6$H$_4$PPh$_2$)$_2$(ZnMe)$_2$] (**2**) at 60 ºC under 1 atm H_2 for (a) 10 min and (b) 2 h. The hydride region of the ^{1}H NMR spectrum (400 MHz, C_6D_6, 298 K) measured at t = 2 h is shown in (c).

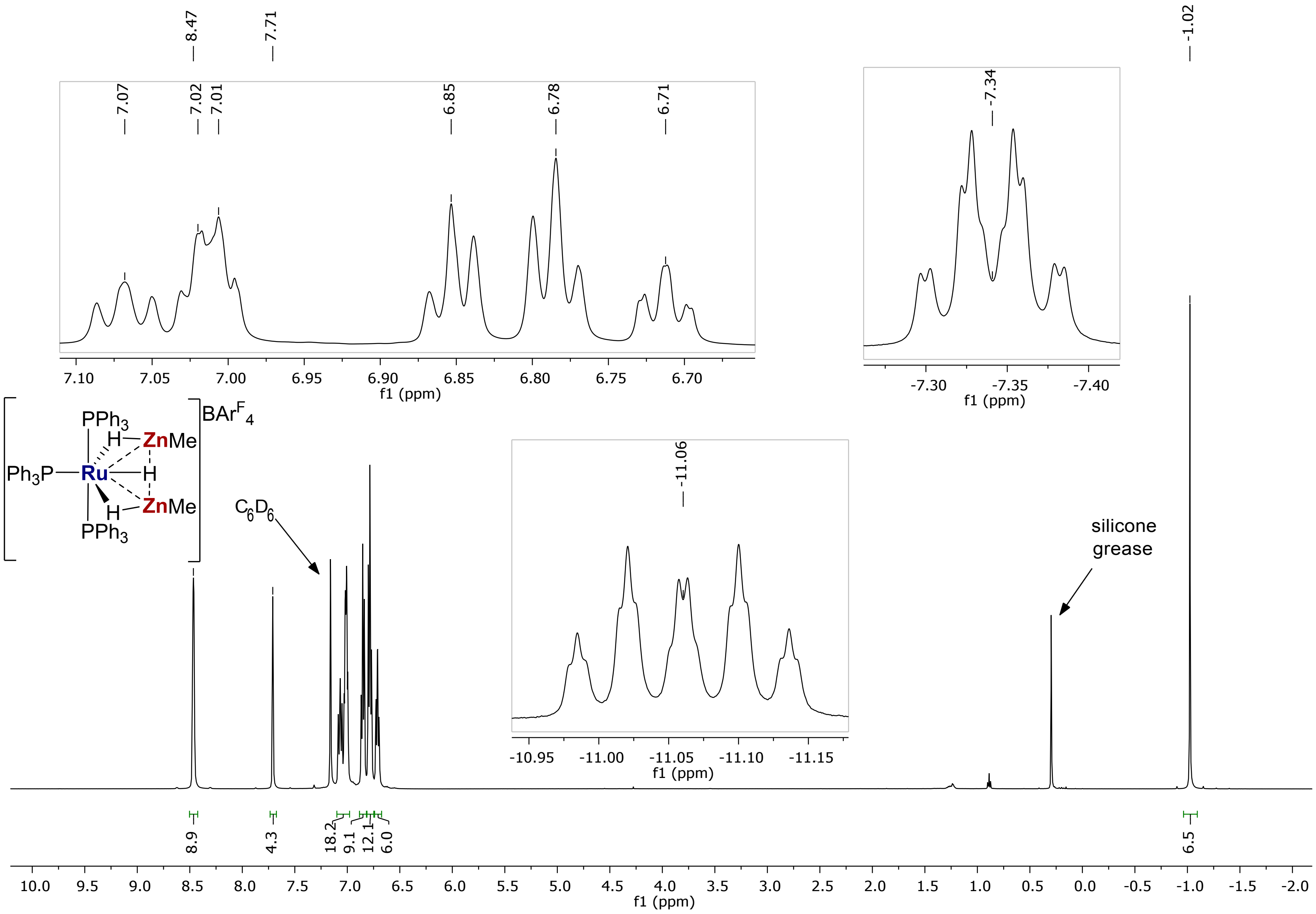

Figure S33. ^{1}H NMR spectrum (500 MHz, C_6D_6, 298 K) of [Ru(PPh$_3$)$_3$(ZnMe)$_2$H$_3$][BAr$^F_4$] (**12**).

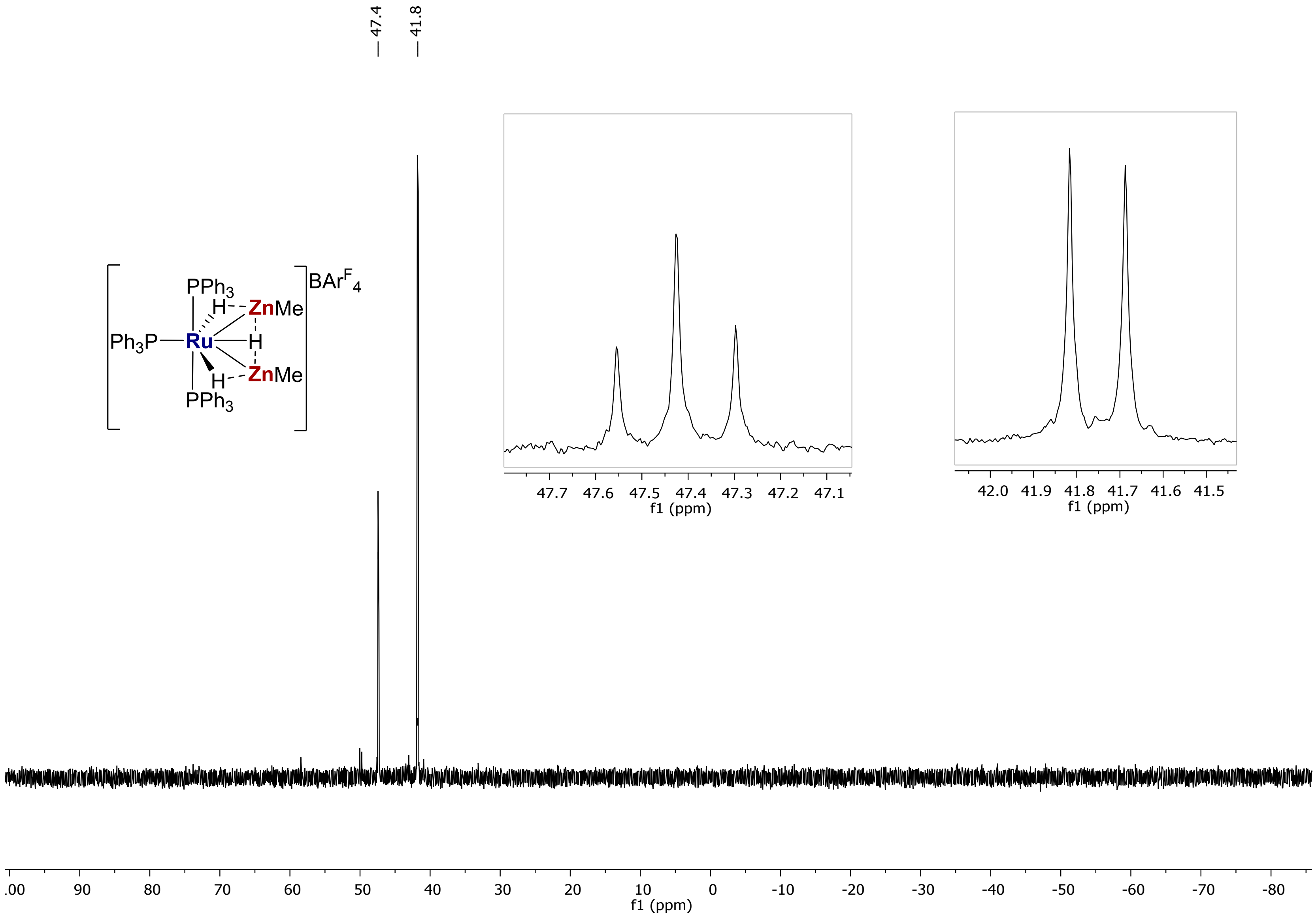

Figure S34. $^{31}P\{^{1}H\}$ NMR spectrum (202 MHz, C_6D_6, 298 K) of $[Ru(PPh_3)_3(ZnMe)_2H_3][BAr^{F}_4]$ (**12**).

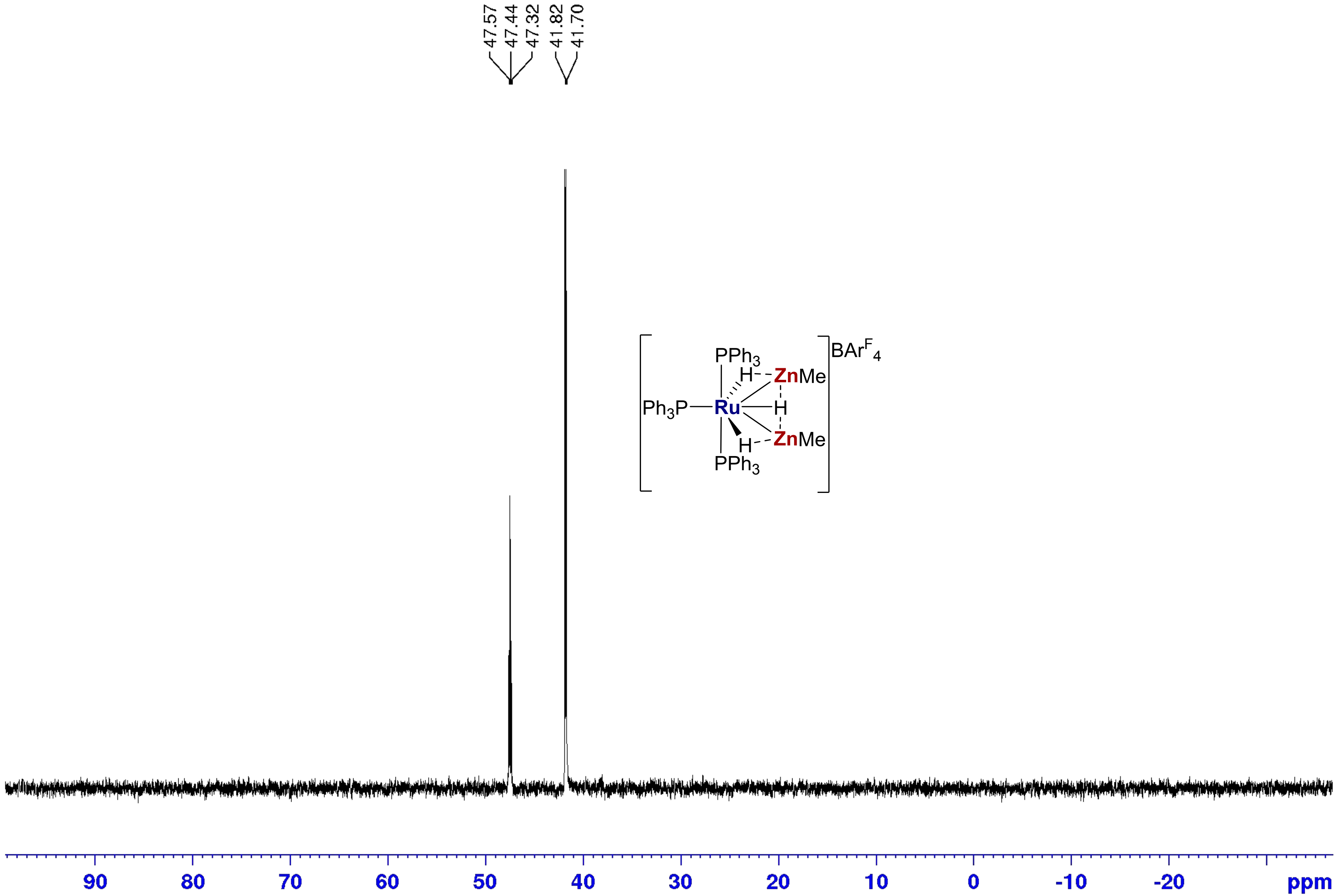

Figure S35. $^{31}P\{^1H\}$ NMR spectrum (202 MHz, C_6D_6, 298 K) showing the clean formation of $[Ru(PPh_3)_3(ZnMe)_2H_3][BAr^F{}_4]$ (**12**) from stirring a powdered sample of solid **2** under 1 atm H_2 for 2 h.

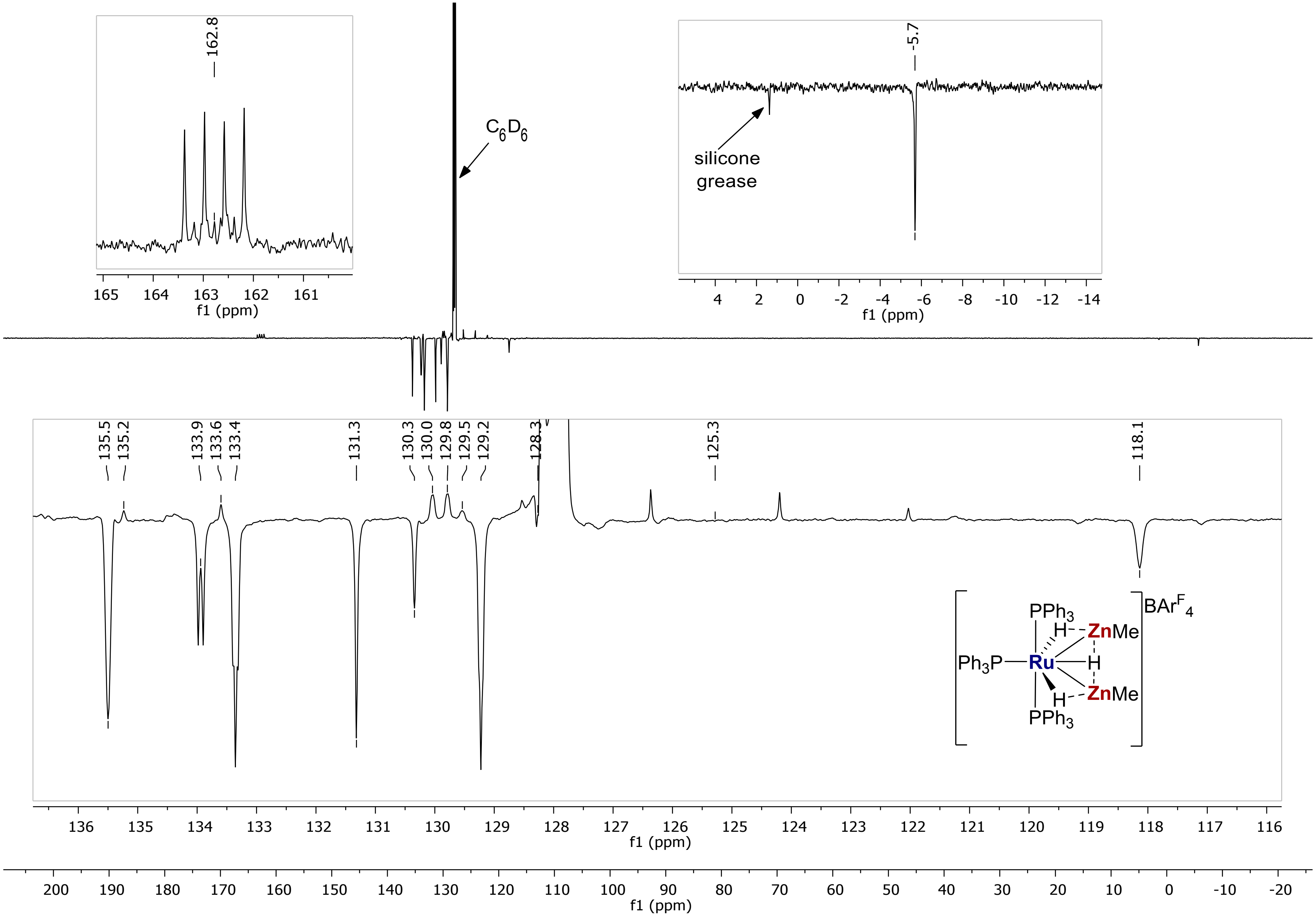

Figure S36. $^{13}C\{^{1}H\}$ (PENDANT) NMR spectrum (126 MHz, C_6D_6, 298 K) of $[Ru(PPh_3)_3(ZnMe)_2H_3][BAr^F{}_4]$ (**12**).

Table S1. Crystal data and structural refinement details for **6**, **7** and **12**.

Compound	**6**	**7**	**12**
Empirical formula	$C_{50}H_{61}P_3RuZn_2$	$C_{87}H_{79}N_2P_3RuZn$	$C_{88}H_{63}BF_{24}P_3RuZn_2$
Formula weight	986.70	1411.87	1911.91
Crystal system	monoclinic	triclinic	triclinic
Space group	$P2_1/n$	P-1	P-1
a/Å	10.3380(1)	14.2690(4)	15.0492(2)
b/Å	18.2601(1)	14.4805(4)	16.8374(3)
c/Å	24.6354(2)	19.9802(5)	18.9130(3)
α/°	90	71.357(3)	63.974(2)
β/°	97.144(1)	75.043(2)	69.958(2)
γ/°	90	64.621(3)	88.022(1)
Volume/Å^3	4614.39(6)	3498.70(19)	4007.32(13)
Z	4	2	2
ρ_{calc}g cm^{-3}	1.420	1.340	1.585
μ/mm^{-1}	5.050	3.181	3.677
F(000)	2040.0	1468.0	1922.0
Crystal size/mm^3	0.125 × 0.110 × 0.089	0.173 × 0.102 × 0.094	0.37 × 0.199 × 0.035
2θ range for data collection/°	6.042 to 146.108	6.926 to 146.396	5.584 to 146.01
Index ranges	$-12 \le h \le 9$, $-22 \le k \le 22$, $-30 \le l \le 30$	$-16 \le h \le 17$, $-13 \le k \le 17$, $-23 \le l \le 24$	$-18 \le h \le 17$, $-20 \le k \le 20$, $-23 \le l \le 18$
Reflections collected, R_{int}	62536	45723	57572
Independent reflections	9194, 0.0771	13926, 0.0370	15938, 0.0390
Data/restraints/parameters	9194/30/600	13926/0/857	15938/156/1167
Goodness-of-fit on F^2	1.041	1.031	1.036
Final R1, wR2 [I>=2σ (I)]	0.0347, 0.0897	0.0283, 0.0684	0.0326, 0.0856
Final R1, wR2 [all data]	0.0385, 0.0933	0.0316, 0.0707	0.0346, 0.0879
Largest diff. peak/hole / e Å^{-3}	0.74/−0.78	0.45/−0.84	0.66/−0.96

Table S2. Selected bond lengths (Å) and angles (°) in **6**, **7** and **12**.

	**6**	**7**	**12**
Ru-Zn	2.4835(4), 2.5109(4)	2.6541(3)	2.5188(3), 2.5387(3)
Ru-PR_3	2.3411(5)	2.3190(4)	2.3784(5), 2.4090(5), 2.3770(5)
Ru-PPh_2	2.3451(5), 2.3743(6)	2.3763(4), 2.3798(4)	-
Ru-C_{aryl}	2.155(2), 2.110(2)	2.0970(17), 2.143(16)	-
Zn···Zn	2.5837(5)	-	-
Zn-Ru-Zn	62.301(11)	-	106.229(10)
P-Ru-P	162.64(2), 98.82(2), 96.68(2),	103.677(15), 101.106(15), 102.660(15)	164.804(17), 99.051(16), 95.598(16)

S-2 Computational Studies

Computational Details. DFT calculations were run with Gaussian 16 (Revision C.01).[4] Ru, Zn and P centers were described with the Stuttgart RECPs and associated basis sets[5] and 6-31G** basis sets were used for all other atoms.[6,7] A set of d-orbital polarization functions was also added to P (ζ^d=0.387).[8] Electronic structure analyses were performed on structures using the heavy atom positions derived from the crystallographic studies with H atom positions optimized with the BP86 functional.[9,10] Details of functional testing on the fully optimised structure of **2** are provided in the Supporting Information. Quantum theory of atoms in molecules (QTAIM)[11] used the AIMALL program.[12] NCI calculations were based on the promolecular densities and used NCIPLOT[13] with visualization via VMD.[14] Natural orbitals for chemical valence (NOCV) analyses[15] were run using the Amsterdam Modeling Suite (AMS) package.[16] Computed geometries are displayed with ChemCraft[17] all geometries are supplied as a separate XYZ file.

S-2.1 Functional Testing on the Geometry of 2.

The structure of **2** was fully optimized using a range of functionals, including GGA functionals (PBE,[18] BP86,[9,10] BLYP,[19] B97D,[20] TPSS[21]), hybrid GGA functionals (PBE0,[22] B3LYP,[23] ωB97XD[24]) and a Minnesota functional (M06[25]). The resulting minima were characterized via analytical frequency calculations (all positive eigenvalues). Key selected distances are compared with experiment in Table S3. In general, good agreement is seen for the Ru−Zn, Ru−C and Zn☹Zn distances. However, the Zn1☹C1 distance is over-estimated by between 0.06 and 0.20 Å while the over-estimation of the Ru-P distances is typical for late TM−P bonds. RMS displacement values on the heavy atom positions suggest a similar performance across all functionals with the B97D, B97D3 and w-B97xD functionals performing less well than the others by this measure.

Given these discrepancies the electronic structure studies were performed using the experimental heavy atom positions from the X-ray structures with only the H atoms positions being optimized.

Table S3. Results of the functional screening conducted on **2**. Key interatomic distances in Å.

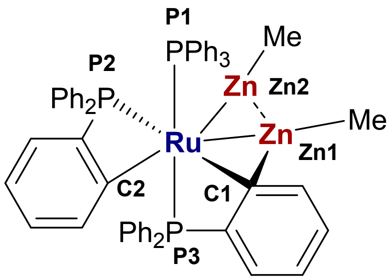

2

Distance/Å	X-Ray	BP86	BLYP	B3LYP	PBE	PBE0	B97D	B97D3	M06	TPSS	ωB97XD
**Ru–Zn1**	2.5103(4)	2.53	2.58	2.56	2.53	2.51	2.56	2.54	2.53	2.52	2.51
**Ru–Zn2**	2.5012(4)	2.52	2.57	2.54	2.52	2.51	2.54	2.52	2.52	2.51	2.50
**Zn1☹Zn2**	2.5898(5)	2.61	2.61	2.61	2.62	2.63	2.64	2.61	2.61	2.60	2.61
**Zn1☹C1**	2.353(2)	2.46	2.53	2.48	2.45	2.41	2.48	2.55	2.45	2.42	2.45
**Ru–C1**	2.173(2)	2.15	2.17	2.16	2.15	2.14	2.15	2.14	2.15	2.15	2.16
**Ru–C2**	2.127(3)	2.14	2.16	2.14	2.13	2.12	2.12	2.13	2.13	2.14	2.12
**Ru–P1**	2.3496(7)	2.42	2.47	2.44	2.41	2.39	2.39	2.38	2.40	2.41	2.38
**Ru–P2**	2.3805(7)	2.45	2.53	2.49	2.44	2.42	2.42	2.42	2.44	2.45	2.40
**Ru–P3**	2.3457(7)	2.40	2.46	2.44	2.39	2.38	2.35	2.35	2.40	2.39	2.39
**RMS Displacement/Å[a]**	-	0.40	0.40	0.39	0.39	0.38	0.51	0.48	0.36	0.38	0.45

[a] heavy atom positions only.

S2-2 Electronic Structure Analyses

Complex 7. Electronic structure analyses of **7** focussing on key interactions around the Ru–Zn vector are shown in Figure S37.

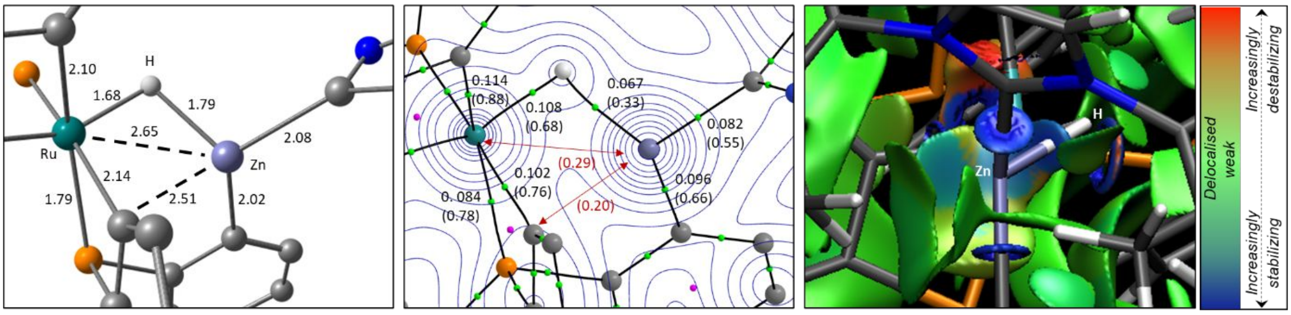

Figure S37. Electronic Structure Analyses of **7**. Left: computed structure with selected distances in Å. Center: QTAIM molecular graphs with bond critical bonds (BCPs) in green and ring critical points (RCPs) in pink are included. Electron density, ρ(r), contour plots are shown along with selected BCP (au) and delocalisation indices in parenthesis; delocalization indices between atoms not linked by a bond path are indicated in red. Right: NCI plots are displayed centered on the Zn–Ru vector. Isosurfaces are generated for σ = 0.3 au and −0.07 < ρ < 0.07 au; a key showing the color scheme employed is also provided.

Complexes 13⁺, 14⁺ and 15⁺. We have previously published aspects of the electronic structure of $[Ru(IPr)_2(CO)(ZnEt)]^+$, $[Ru(IPr)_2(CO)(ZnEt)H_2]^+$ and $[Ru(IPr)_2(CO)(ZnEt)(\eta^2\text{-}H_2)H_2]^+$,[26] and here we provide complete analyses of the Me analogues of these species for direct comparison with ZnMe-containing **2** and **3**. (see Figure S38 and Figures S43-S45).

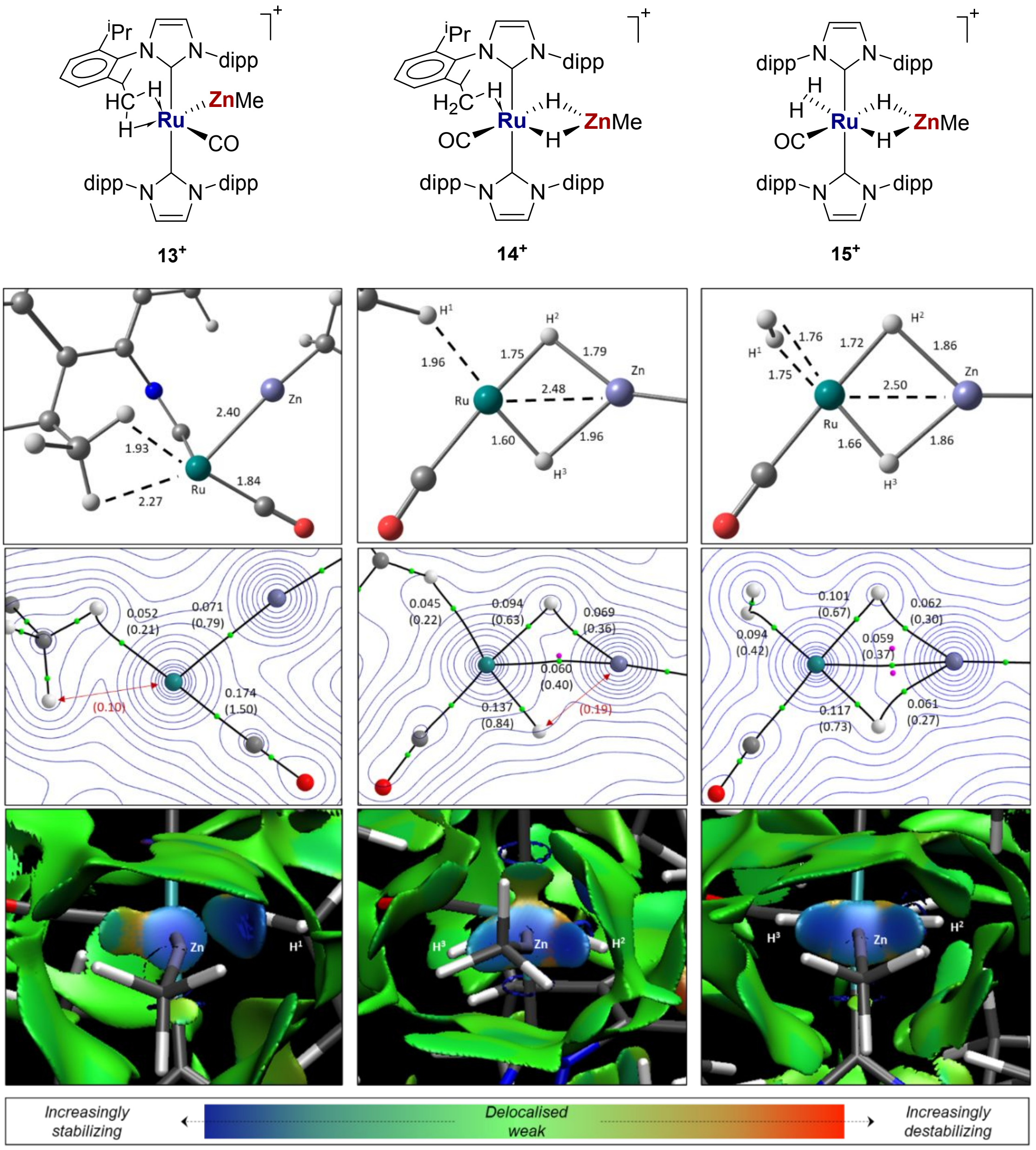

Figure S38. Electronic Structure Analyses of **13⁺, 14⁺** and **15⁺**. Top: computed structure with selected distances in Å. Center: QTAIM molecular graphs with bond critical bonds (BCPs) in green and ring critical points (RCPs) in pink are included. Electron density, ρ(r), contour plots are shown along with selected BCP (au) and delocalisation indices in parenthesis; delocalization indices between atoms not linked by a bond path are indicated in red. Bottom: NCI plots are displayed centered on the Zn–Ru vector. Isosurfaces are generated for $\sigma = 0.3$ au and $-0.07 < \rho < 0.07$ au; a key showing the color scheme employed is also provided.

Computed QTAIM Metrics

Relevant bond critical point data associated with the computed complexes are provided in atomic units. Selected parameters are defined as: ρ(r) electron density, $\nabla^2\rho$(r) Laplacian of electron density, ε ellipticity, H(r) local energy density, δ(A,B) delocalization index.

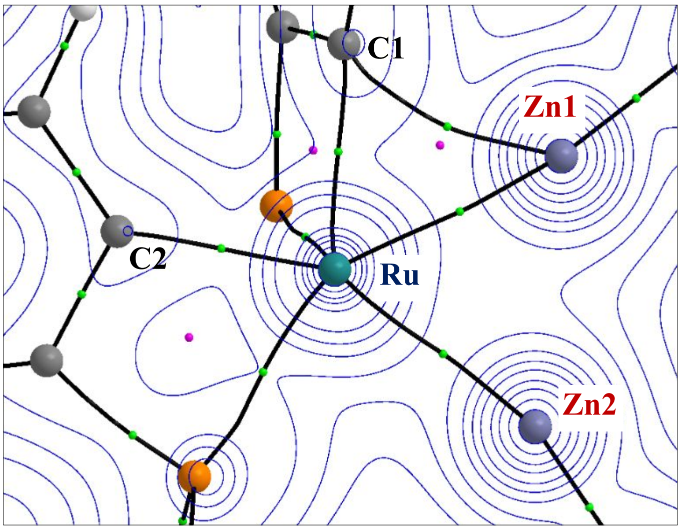

Bond	ρ(r)	$\nabla^2\rho$(r)	ε	H (r)	δ (A,B)
**Ru–Zn1**	0.059	0.066	0.157	-0.019	0.515
**Ru–Zn2**	0.061	0.062	0.048	-0.020	0.675
**Zn1–C1**	0.049	0.086	2.703	-0.010	0.256
**Ru–C1**	0.098	0.170	0.083	-0.032	0.759
**Ru–C2**	0.109	0.185	0.126	-0.040	0.804

Figure S39. Molecular graph and associated bond critical point data for complex **2**.

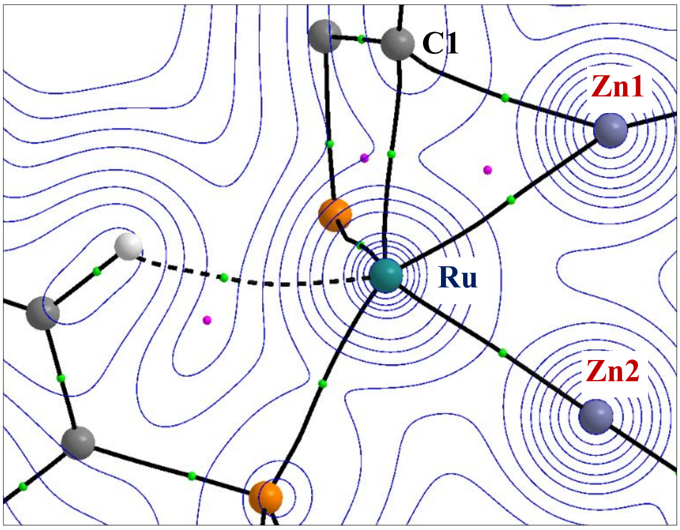

Bond	ρ(r)	$\nabla^2\rho$(r)	Ellipticity	H (r)	δ (A,B)
**Ru–Zn1**	0.054	0.066	0.563	-0.017	0.461
**Ru–Zn2**	0.071	0.101	0.039	-0.023	0.820
**Ru–C1**	0.091	0.170	0.090	-0.029	0.715
**Zn1–C1**	0.070	0.157	0.293	-0.016	0.401

Figure S40. Molecular graph and associated bond critical point data for complex **3**.

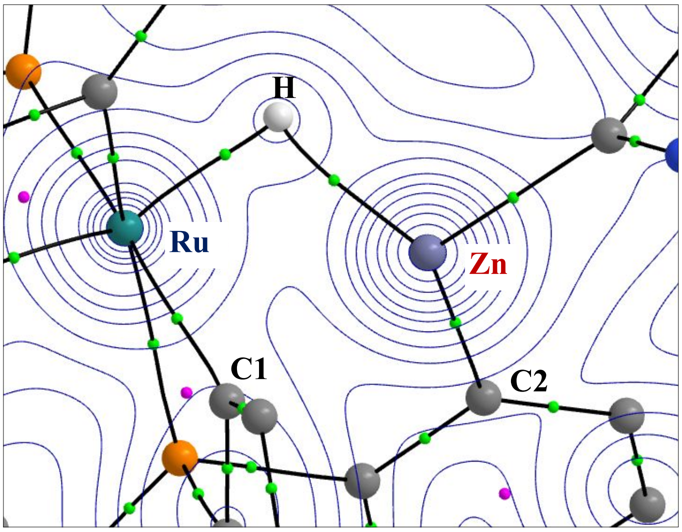

Bond	ρ(r)	$\nabla^2\rho$(r)	Ellipticity	H (r)	δ (A,B)
**Ru–H**	0.108	0.183	0.084	-0.042	0.680
**Zn–H**	0.067	0.128	0.221	-0.018	0.327
**Ru–C1**	0.102	0.195	0.113	-0.035	0.759
**Zn–C2**	0.096	0.248	0.021	-0.027	0.657

Figure S41. Molecular graph and associated bond critical point data for complex **7**.

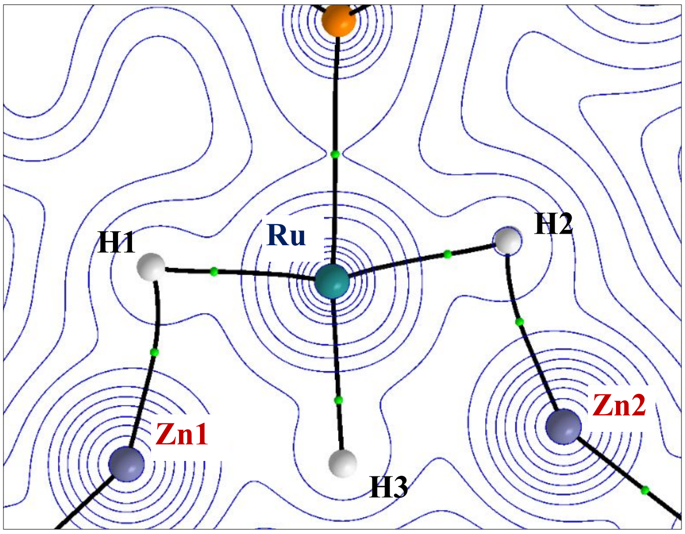

Bond	ρ(r)	$\nabla^2\rho$(r)	Ellipticity	H (r)	δ (A,B)
**Ru–H1**	0.106	0.184	0.022	-0.041	0.692
**Ru–H2**	0.106	0.191	0.033	-0.041	0.678
**Ru–H3**	0.107	0.199	0.145	-0.042	0.698
**H1–Zn1**	0.064	0.108	0.531	-0.017	0.313
**H2–Zn2**	0.068	0.117	0.439	-0.018	0.320
**H–Zn**[a]	0.113	0.189	0.000	-0.040	0.890

Figure S42. Molecular graph and associated bond critical point data for complex **12**.[a] H–Zn bond in ZnMeH.

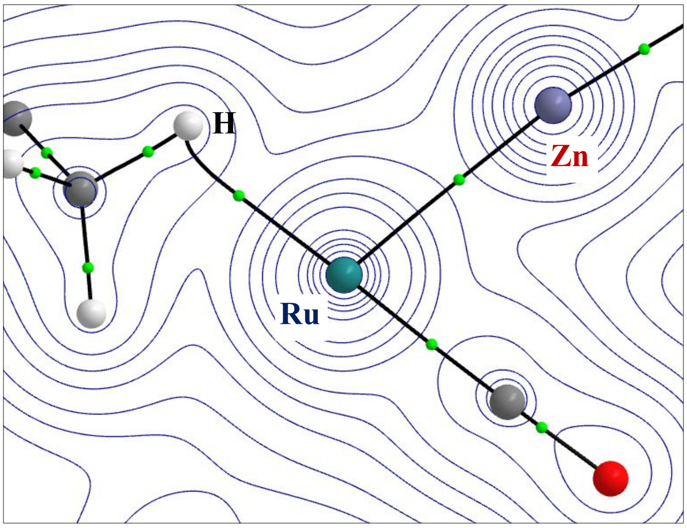

Bond	ρ(r)	$\nabla^2\rho$(r)	Ellipticity	H (r)	δ (A,B)
**Ru–H**	0.052	+0.238	0.823	-0.004	0.214
**Ru–Zn**	0.071	+0.110	0.005	+0.023	0.792

Figure S43. Molecular graph and associated bond critical point data for complex **13**$^+$.

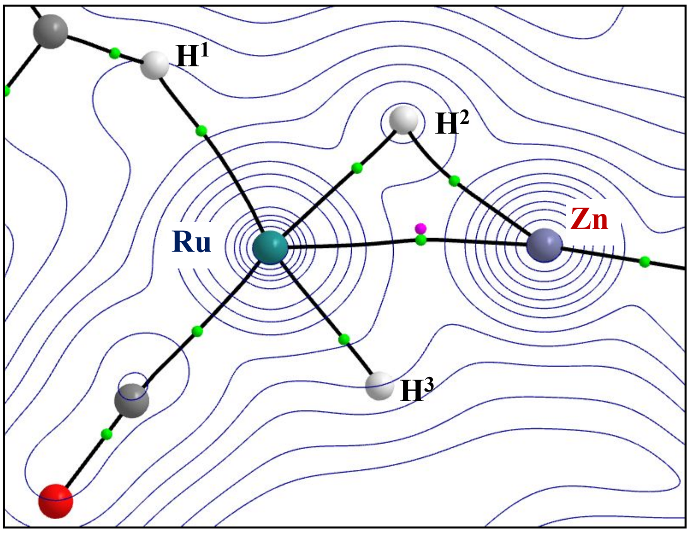

Bond	ρ(r)	$\nabla^2\rho$(r)	Ellipticity	H (r)	δ (A,B)
**Ru–H^1**	0.045	0.156	0.207	-0.007	0.220
**Ru–H^2**	0.094	0.192	0.153	-0.033	0.631
**Ru–H^3**	0.137	0.138	0.018	-0.067	0.848
**H^2–Zn**	0.069	0.122	0.397	-0.019	0.355
**Ru–Zn**	0.060	0.098	3.710	-0.018	0.398

Figure S44. Molecular graph and associated bond critical point data for complex **14$^+$**.

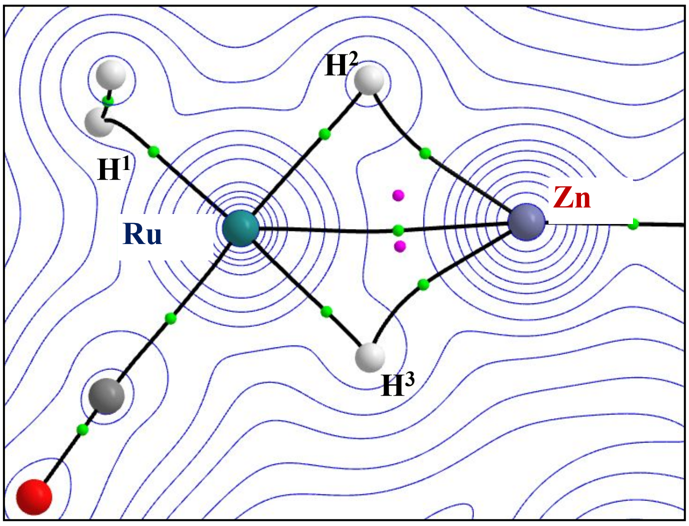

Bond	ρ(r)	$\nabla^2\rho$(r)	Ellipticity	H (r)	δ (A,B)
**Ru–H^1**	0.094	0.361	5.704	-0.026	0.420
**Ru–H^2**	0.101	0.184	0.110	-0.038	0.669
**Ru–H^3**	0.117	0.179	0.066	-0.050	0.733
**H^2–Zn**	0.062	0.105	0.668	-0.016	0.302
**H^3–Zn**	0.061	0.108	0.996	-0.015	0.268
**Ru–Zn**	0.059	0.092	4.812	-0.017	0.369

Figure S45. Molecular graph and associated bond critical point data for complex **15**$^+$.

ETS-NOCV analyses.

The ETS-NOCV method was employed to assess the interaction between the different $\{ZnMe\}^+$ fragments and the remaining Ru-based fragments within complexes **2**, **3** and **12**. The results of energy decomposition analysis for each fragmentation pattern are displayed in Table S4. The major deformation density channel of each structure is shown in Figures S46-48, along with its interaction energy, ΔE_{Orb} and charge transfer, Δq. All the figures used the same isovalue (0.0025) for the contour plots so that they could be compared to one another. Electron flow is shown from red to blue (most H atoms and some phenyl rings were omitted for clarity).

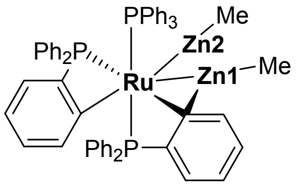

2

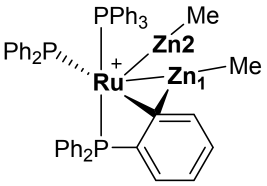

3

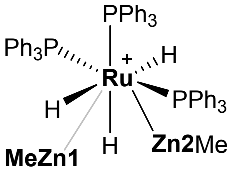

12

	**2**		**3**		**12**	
	**$\{Zn1Me\}^+$**	**$\{Zn2Me\}^+$**	**$\{Zn1Me\}^+$**	**$\{Zn2Me\}^+$**	**$\{Zn1Me\}^+$**	**$\{Zn2Me\}^+$**
**ΔE_{Pauli}**	146.3	150.9	142.8	127.00	125.0	122.3
**ΔV_{elec}**	-197.9	-200.6	-127.6	-116.9	-121.2	-122.6
**ΔE_{oi}**	-131.2	-141.0	-120.2	-122.8	-111.6	-109.7
**ΔE_{tot}**	-182.8	-190.8	-104.9	-112.7	-107.9	-110.1

Table S4. Energy decomposition analysis of the two different fragmentation patterns of **2**, **3** and **13**. Energies in kcal/mol correspond to the Pauli repulsion, ΔE_{Pauli}, electrostatic interaction, ΔV_{elec}, orbital interaction, ΔE_{oi}, and total bonding energy, ΔE_{tot}.

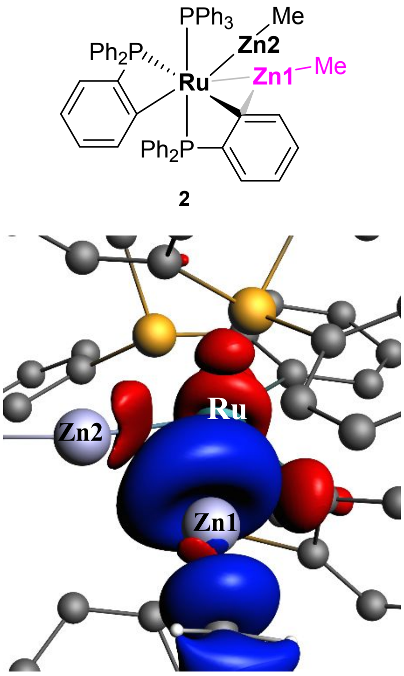

ΔE_{Orb} = -68.6 kcal/mol
Δq = -0.89

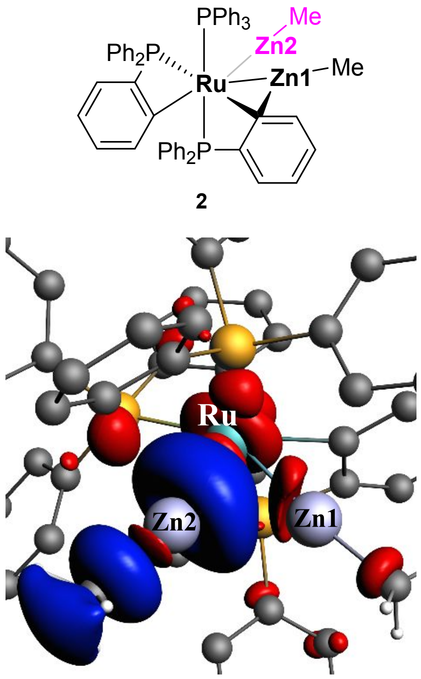

ΔE_{Orb} = -86.3 kcal/mol
Δq = -1.05

Figure S46. Main ETS-NOCV deformation density channel plot of **2** and associative metrics.

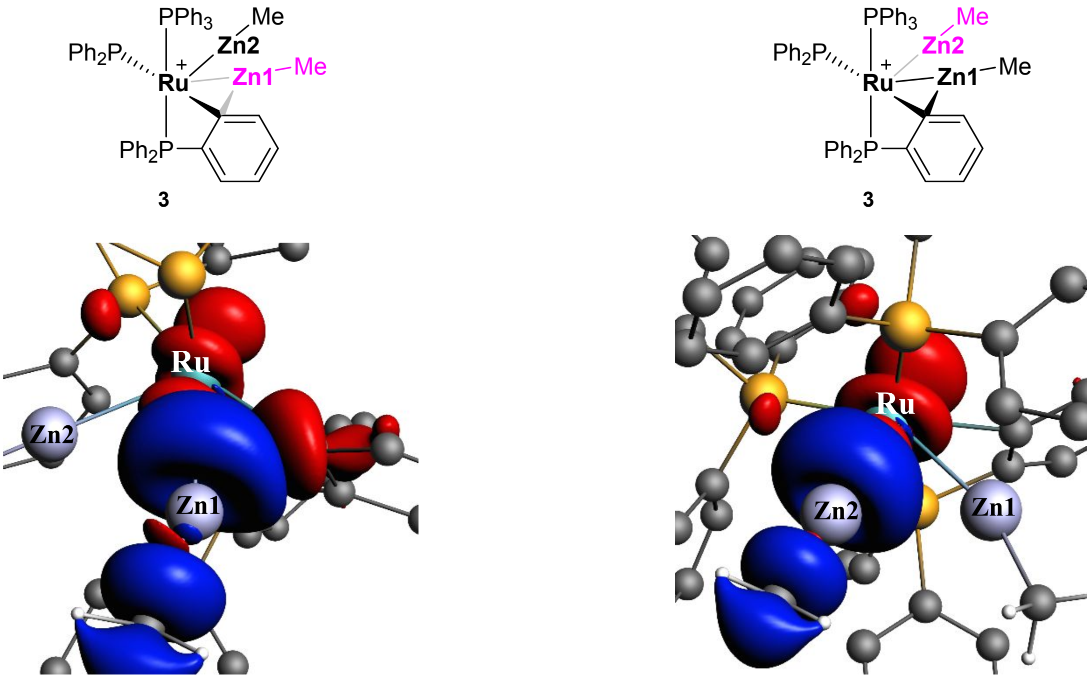

ΔE_{Orb} = -54.3 kcal/mol
Δq = -0.73

ΔE_{Orb} = -69.5 kcal/mol
Δq = -0.84

Figure S47. Main ETS-NOCV deformation density channel plot of **3** and associative metrics.

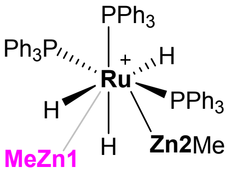

12

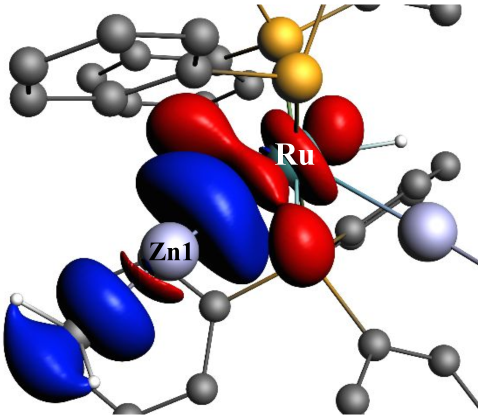

ΔE_{Orb} = -52.2 kcal/mol
Δq = -0.80

Figure S48. Main ETS-NOCV deformation density channel plot of **12** and associative metrics. Results with the $\{Zn2Me\}^+$ fragment were almost identical and are not shown.

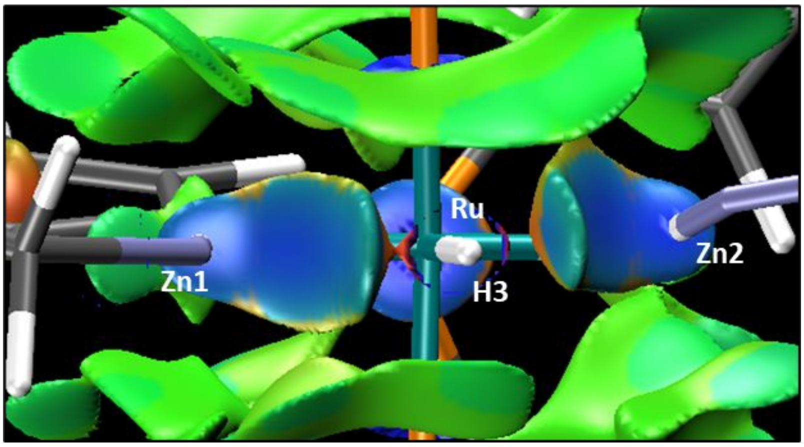

Figure S49. NCI plot of species **12** viewed down the Zn1-H1 vector. Isosurfaces are generated for $\sigma = 0.3$ au and $-0.07 < \rho < 0.07$ au; a key showing the color scheme employed is also provided.

S-3 Computed Structures (A) and Energies (atomic units).

2 (H atoms optimized)
BP86 energy = -2732.69433041

Ru	0.07022	-0.38304	0.17535
Zn	0.06566	-0.42625	-2.32542
Zn	0.20524	-2.61979	-0.95586
P	2.36721	-0.81782	0.41097
P	0.28279	1.97995	-0.01925
P	-2.27374	-0.46803	0.14666
C	0.22224	0.10405	-4.23403
H	-0.08357	1.15313	-4.40234
H	-0.43396	-0.53441	-4.85370
H	1.25282	-0.01433	-4.61585
C	0.51193	-4.47119	-1.52522
H	1.59226	-4.63254	-1.68519
H	-0.00868	-4.70057	-2.47194
H	0.16612	-5.18081	-0.75429
C	0.47526	-2.17301	1.33786
C	-0.22081	-3.13939	2.06752
H	-1.30112	-3.25971	1.94936
C	0.44334	-3.97125	2.97675
H	-0.13207	-4.72134	3.53282
C	1.81007	-3.85457	3.19632
H	2.30769	-4.50265	3.92667
C	2.54159	-2.91406	2.48645
H	3.61783	-2.79548	2.66207
C	1.87572	-2.12108	1.55545
C	3.35108	-1.65664	-0.90236
C	4.06224	-2.81063	-0.62083
H	4.07815	-3.22081	0.39270
C	4.73403	-3.47914	-1.63922
H	5.28076	-4.39969	-1.40478
C	4.69300	-3.00904	-2.93109
H	5.20843	-3.55316	-3.73058
C	4.01141	-1.85516	-3.20839
H	3.97399	-1.46226	-4.23061
C	3.34068	-1.18039	-2.19536
H	2.78213	-0.27030	-2.44070
C	3.57893	0.30213	1.19340
C	3.63457	0.42849	2.58604
H	3.01325	-0.20990	3.21776
C	4.46056	1.38315	3.15954
H	4.49176	1.48027	4.25055
C	5.23344	2.20382	2.36677
H	5.88153	2.95876	2.82679
C	5.19847	2.07189	0.99365
H	5.81785	2.71721	0.36141
C	4.37841	1.12199	0.39858
H	4.35557	1.03205	-0.69202
C	0.30753	0.61815	2.03660
C	0.39848	0.21999	3.37640
H	0.27102	-0.83619	3.64746
C	0.64252	1.15008	4.38146
H	0.70272	0.81592	5.42548
C	0.82109	2.50177	4.08460
H	1.01097	3.22383	4.88650
C	0.73558	2.92834	2.76688
H	0.83580	3.99494	2.53127
C	0.49228	1.98361	1.77108
C	-1.02348	3.22647	-0.39933
C	-1.87942	3.67678	0.58902
H	-1.76481	3.31934	1.61711
C	-2.89103	4.57104	0.28554
H	-3.55501	4.91678	1.08520
C	-3.06414	5.00883	-0.99267
H	-3.87115	5.71244	-1.22967
C	-2.21642	4.57479	-2.00363
H	-2.34328	4.93573	-3.02997
C	-1.19328	3.69897	-1.69678
H	-0.50355	3.38193	-2.48873
C	1.69419	2.83295	-0.81639
C	2.08136	2.48505	-2.10221
H	1.58210	1.65446	-2.61593
C	3.09592	3.18383	-2.76716
H	3.37827	2.88795	-3.78343
C	3.71993	4.23441	-2.13933
H	4.51551	4.78844	-2.65108
C	3.34284	4.59029	-0.86705
H	3.83770	5.42603	-0.35926
C	2.32784	3.89814	-0.20257
H	2.05499	4.19879	0.81204
C	-3.22560	0.61741	1.30349
C	-2.84477	0.66826	2.64409
H	-2.00107	0.06678	2.98922
C	-3.51005	1.48740	3.54090
H	-3.18789	1.51347	4.58789
C	-4.57626	2.27495	3.11630
H	-5.10270	2.92374	3.82492
C	-4.96166	2.22631	1.78490
H	-5.79974	2.84089	1.43588
C	-4.29824	1.41544	0.88695
H	-4.61533	1.40706	-0.15874
C	-3.19437	-0.13636	-1.43000
C	-2.69794	0.73869	-2.36871
H	-1.75432	1.25744	-2.18247
C	-3.39363	0.99910	-3.55119
H	-2.95688	1.69384	-4.27731
C	-4.57913	0.38910	-3.80474
H	-5.11709	0.57915	-4.74041
C	-5.12485	-0.46314	-2.85166
H	-6.09392	-0.94193	-3.03318
C	-4.44430	-0.72719	-1.67611
H	-4.89048	-1.39904	-0.93775
C	-3.00927	-2.12414	0.51670
C	-2.81081	-3.13574	-0.42230
H	-2.25975	-2.92742	-1.34748
C	-3.34222	-4.40480	-0.22006
H	-3.17812	-5.18776	-0.96780
C	-4.09642	-4.65561	0.90911
H	-4.53062	-5.65006	1.06479
C	-4.29604	-3.66558	1.85115
H	-4.89124	-3.87017	2.74817
C	-3.75013	-2.39642	1.66332
H	-3.92707	-1.61348	2.40605

3 (H atoms optimized)
BP86 energy = -2733.13583983

Ru	-0.03277	-0.26153	0.25189
Zn	-0.00939	-2.73906	-0.35258
Zn	-0.02886	-0.72515	-2.11370
P	2.28690	-0.72496	0.57264
P	0.36892	1.95765	-0.44189

```
P  -2.35872 -0.36803  0.25476
C  -0.01464 -1.11693 -4.02647
H   1.01935 -1.06905 -4.40290
H  -0.63819 -0.39406 -4.57849
H  -0.41049 -2.13054 -4.20392
C   0.12494 -4.38111 -1.38236
H  -0.51497 -4.33834 -2.27831
H  -0.18879 -5.24023 -0.76576
H   1.16917 -4.53081 -1.70102
C   0.30370 -1.94616  1.61644
C  -0.41653 -2.76751  2.51084
H  -1.46704 -3.01062  2.32343
C   0.19493 -3.26128  3.65036
H  -0.38264 -3.89324  4.33504
C   1.52148 -2.97225  3.94783
H   1.97517 -3.36899  4.86231
C   2.26356 -2.19059  3.08514
H   3.30902 -1.94610  3.30435
C   1.66391 -1.74363  1.92043
C   3.05108 -1.85680 -0.63040
C   3.11391 -1.49417 -1.97291
H   2.74046 -0.51531 -2.29497
C   3.64754 -2.35939 -2.91322
H   3.68474 -2.05969 -3.96564
C   4.13542 -3.57713 -2.51750
H   4.55629 -4.26477 -3.25948
C   4.09531 -3.94909 -1.19075
H   4.48592 -4.92322 -0.87843
C   3.55532 -3.09084 -0.24188
H   3.51782 -3.39808  0.80868
C   3.69026  0.21287  1.25896
C   4.91902  0.27561  0.62057
H   5.08755 -0.25645 -0.32047
C   5.94427  1.02626  1.18257
H   6.91340  1.06529  0.67226
C   5.76494  1.68546  2.35905
H   6.58719  2.26687  2.79129
C   4.55451  1.62536  3.00364
H   4.39573  2.14718  3.95254
C   3.52529  0.88597  2.44703
H   2.56598  0.84269  2.97149
C   1.72261  2.11596 -1.66739
C   1.43338  1.85863 -3.00712
H   0.39848  1.71548 -3.33676
C   2.45369  1.76959 -3.95212
H   2.20142  1.56591 -4.99803
C   3.76404  1.93531 -3.56554
H   4.57050  1.86237 -4.30286
C   4.05664  2.21893 -2.24555
H   5.09619  2.36775 -1.93469
C   3.04929  2.30498 -1.29340
H   3.31033  2.50880 -0.25261
C  -0.86120  3.22188 -0.95657
C  -1.94886  3.39758 -0.09746
H  -2.03546  2.78399  0.80466
C  -2.90718  4.34731 -0.35684
H  -3.74356  4.47389  0.33929
C  -2.81608  5.13284 -1.48295
H  -3.58319  5.88579 -1.69350
C  -1.75182  4.97696 -2.33846
H  -1.66445  5.61108 -3.22772
C  -0.76761  4.03907 -2.07946
H   0.08602  3.95934 -2.75865
C   0.90852  2.75375  1.10530
C   0.41520  2.20779  2.28349
H  -0.19770  1.29330  2.24062
C   0.65987  2.79740  3.49702
H   0.24629  2.35296  4.40915
C   1.40523  3.95020  3.56559
H   1.59987  4.42397  4.53383
C   1.90483  4.50986  2.41147
H   2.49129  5.43390  2.45691
C   1.63291  3.93695  1.17582
H   1.99493  4.42304  0.26374
C  -3.07218  0.47968  1.72090
C  -2.52917  0.19909  2.97467
H  -1.69508 -0.50728  3.06083
C  -3.05185  0.77784  4.11919
H  -2.62605  0.52219  5.09586
C  -4.09240  1.67472  4.02847
H  -4.50017  2.13947  4.93246
C  -4.61945  1.98646  2.79306
H  -5.44663  2.70041  2.71368
C  -4.12298  1.39009  1.64091
H  -4.56577  1.63921  0.67239
C  -3.07680 -2.05728  0.40515
C  -3.70249 -2.53451  1.55523
H  -3.81355 -1.88577  2.42915
C  -4.20746 -3.82025  1.60114
H  -4.69958 -4.17183  2.51470
C  -4.11222 -4.64938  0.50485
H  -4.52012 -5.66485  0.54277
C  -3.50951 -4.18660 -0.65076
H  -3.44300 -4.82910 -1.53483
C  -2.99511 -2.90232 -0.69915
H  -2.56219 -2.53896 -1.63954
C  -3.38482  0.21446 -1.15758
C  -4.74101 -0.09367 -1.21207
H  -5.20573 -0.67456 -0.40796
C  -5.51308  0.32381 -2.27405
H  -6.57890  0.07057 -2.29386
C  -4.95545  1.03565 -3.31478
H  -5.57554  1.35451 -4.15915
C  -3.61627  1.34439 -3.27944
H  -3.15775  1.91206 -4.09619
C  -2.83600  0.93211 -2.21657
H  -1.77409  1.18976 -2.19732
```

7 (H atoms optimized)

```
BP86 energy =     -3349.79593122

Ru -0.59905  0.16976 -0.24351
Zn  1.97236  0.02533  0.39694
P  -2.35600  1.67952 -0.77371
P  -0.51723  0.21442  2.13443
P  -1.61531 -1.86871 -0.67885
N   4.49465 -1.04868 -0.94992
N   4.58567  1.08100 -1.00196
C  -0.41557  0.98313 -2.16763
C   0.51280  0.93710 -3.21505
H   1.39346  0.28684 -3.15009
C   0.31251  1.68723 -4.37772
H   1.04589  1.62104 -5.19293
C  -0.80058  2.50167 -4.53323
H  -0.94488  3.06192 -5.46455
C  -1.72077  2.60507 -3.50677
```

H	-2.61256	3.23549	-3.61270
C	-1.49676	1.86685	-2.34531
C	-2.49685	3.30647	0.03899
C	-2.85991	3.33890	1.38351
H	-2.99626	2.39988	1.93180
C	-3.05262	4.54672	2.04077
H	-3.34841	4.55242	3.09581
C	-2.85671	5.73767	1.36139
H	-3.00169	6.69419	1.87687
C	-2.46631	5.72115	0.02759
H	-2.30133	6.66297	-0.50821
C	-2.29079	4.51336	-0.63158
H	-1.98040	4.50391	-1.68101
C	-4.13166	1.40621	-1.22012
C	-5.18650	1.92639	-0.47439
H	-4.98935	2.53909	0.41008
C	-6.50582	1.70700	-0.86574
H	-7.31954	2.14097	-0.27284
C	-6.78723	0.95477	-1.98909
H	-7.82532	0.78352	-2.29565
C	-5.74592	0.42268	-2.72624
H	-5.95326	-0.17779	-3.61904
C	-4.42605	0.64886	-2.34995
H	-3.61468	0.23374	-2.95593
C	0.34091	1.93327	0.53262
C	0.83390	3.11916	-0.01378
H	1.02871	3.19373	-1.09021
C	1.00875	4.24497	0.80199
H	1.32856	5.19056	0.35244
C	0.77134	4.18543	2.16306
H	0.92700	5.07474	2.78561
C	0.33496	3.00096	2.74760
H	0.18858	2.94694	3.83274
C	0.10082	1.91410	1.92656
C	2.08019	-0.59218	2.31790
C	3.18720	-1.13583	2.98117
H	4.16411	-1.13945	2.48365
C	3.10020	-1.69290	4.25947
H	3.98910	-2.13327	4.72924
C	1.89409	-1.67610	4.93336
H	1.81058	-2.10291	5.93999
C	0.77342	-1.09588	4.33056
H	-0.17595	-1.06288	4.87570
C	0.87580	-0.57970	3.04183
C	-1.86401	0.14114	3.36313
C	-2.18081	1.17445	4.23241
H	-1.66526	2.13481	4.15222
C	-3.14305	0.99619	5.22114
H	-3.37065	1.81617	5.91198
C	-3.80294	-0.21256	5.33474
H	-4.55826	-0.35295	6.11670
C	-3.52555	-1.23899	4.45063
H	-4.05563	-2.19478	4.52956
C	-2.55174	-1.07100	3.47051
H	-2.31000	-1.89853	2.79405
C	-1.22029	-2.70153	-2.27933
C	-1.29458	-1.93663	-3.44494
H	-1.55246	-0.87656	-3.37437
C	-1.02432	-2.49718	-4.68639
H	-1.09059	-1.87705	-5.58722
C	-0.65904	-3.83494	-4.78060
H	-0.44195	-4.28025	-5.75824
C	-0.57811	-4.60611	-3.62957
H	-0.30158	-5.66518	-3.69917
C	-0.85322	-4.04964	-2.38750
H	-0.79769	-4.67251	-1.48940
C	-3.45363	-2.03897	-0.67651
C	-4.16740	-1.38446	0.32313
H	-3.63428	-0.73697	1.02708
C	-5.54423	-1.53723	0.42729
H	-6.08760	-1.01165	1.21980
C	-6.22937	-2.33861	-0.47617
H	-7.31589	-2.45528	-0.39569
C	-5.53075	-2.97742	-1.48301
H	-6.06179	-3.60704	-2.20692
C	-4.15280	-2.83022	-1.58882
H	-3.61587	-3.35478	-2.38493
C	-1.20136	-3.21972	0.51925
C	-2.10479	-4.22138	0.88069
H	-3.11432	-4.23197	0.45836
C	-1.73023	-5.21987	1.77427
H	-2.45461	-5.99777	2.04364
C	-0.46044	-5.23149	2.32947
H	-0.18137	-6.01041	3.04864
C	0.43415	-4.24176	1.98596
H	1.43576	-4.21274	2.42806
C	0.07070	-3.25379	1.08260
H	0.78635	-2.46632	0.83296
C	3.77141	0.05952	-0.64294
C	5.73373	-0.72381	-1.48200
H	6.44331	-1.48543	-1.79249
C	5.78965	0.61404	-1.51217
H	6.57290	1.29485	-1.83514
C	4.09503	-2.40986	-0.67849
C	3.10558	-2.99713	-1.47657
C	2.72921	-4.29736	-1.16677
H	1.95059	-4.77055	-1.77600
C	3.32985	-5.01924	-0.13896
C	4.34234	-4.41225	0.59443
H	4.84338	-4.97273	1.39294
C	4.75067	-3.10275	0.33414
C	2.48736	-2.28112	-2.64953
H	1.66626	-1.62034	-2.32158
H	2.05488	-3.00263	-3.35962
H	3.22319	-1.65795	-3.18486
C	2.89467	-6.43236	0.15988
H	1.90891	-6.43759	0.65992
H	3.60944	-6.94803	0.82165
H	2.79255	-7.02772	-0.76374
C	5.91323	-2.52452	1.11701
H	6.87805	-2.77419	0.63660
H	5.94006	-2.94745	2.13375
H	5.86943	-1.42826	1.20381
C	4.32511	2.48686	-0.82147
C	4.06653	3.26641	-1.94409
C	3.88182	4.63367	-1.75704
H	3.67353	5.26497	-2.62896
C	3.95165	5.20134	-0.49350
C	4.21854	4.39135	0.60291
H	4.26637	4.83284	1.60452
C	4.40271	3.01375	0.46487
C	3.97871	2.67340	-3.32567
H	4.86974	2.08064	-3.59456
H	3.86227	3.46712	-4.08000
H	3.10412	2.00438	-3.41050
C	3.71236	6.68251	-0.32017

```
H   4.00350  7.02858  0.68476
H   2.64473  6.93408 -0.45916
H   4.27723  7.27459 -1.06107
C   4.61842  2.15207  1.68034
H   3.70313  1.59447  1.95051
H   4.89748  2.77020  2.54836
H   5.41479  1.40318  1.52615
H   0.75340 -0.74081 -0.66195
```

12 (H atoms optimized)

BP86 energy =   -2735.57166107

```
Ru  0.00816 -0.35771  0.01332
Zn -0.35970 -1.63891  2.15053
Zn  0.11345 -2.08797 -1.84169
P  -2.33935 -0.72216 -0.10110
P   0.01834  2.05118  0.01154
P   2.37213 -0.59972 -0.03822
C  -0.64883 -2.78166  3.68261
H  -1.70760 -2.74039  3.98146
H  -0.02992 -2.44366  4.53078
H  -0.38215 -3.82939  3.46364
C   0.13898 -3.43280 -3.23503
H  -0.59222 -4.22366 -3.00983
H   1.14324 -3.87798 -3.30800
H  -0.11884 -2.98501 -4.20979
C  -2.75143 -2.27978 -0.99659
C  -2.31630 -3.51481 -0.48974
H  -1.72967 -3.55698  0.43528
C  -2.64016 -4.69754 -1.13176
H  -2.31183 -5.65126 -0.70498
C  -3.34858 -4.67335 -2.32496
H  -3.57883 -5.60841 -2.84595
C  -3.76904 -3.46608 -2.84149
H  -4.33794 -3.43587 -3.77687
C  -3.50384 -2.27715 -2.17020
H  -3.88128 -1.33844 -2.58282
C  -3.13489 -0.97571  1.53468
C  -3.84232 -2.12406  1.88873
H  -4.01619 -2.92467  1.16629
C  -4.33923 -2.25798  3.18709
H  -4.87928 -3.17288  3.45466
C  -4.17652 -1.25745  4.11737
H  -4.58018 -1.37616  5.12857
C  -3.50444 -0.10206  3.76574
H  -3.37378  0.71118  4.48691
C  -2.97102  0.02940  2.49103
H  -2.42174  0.93764  2.23624
C  -3.44665  0.47079 -0.93688
C  -4.68395  0.83210 -0.40966
H  -5.01923  0.41175  0.54308
C  -5.49930  1.73632 -1.08272
H  -6.46859  2.01125 -0.65323
C  -5.09099  2.27898 -2.28775
H  -5.73297  2.99214 -2.81576
C  -3.86731  1.91480 -2.82999
H  -3.53526  2.33930 -3.78300
C  -3.04367  1.01548 -2.15532
H  -2.07437  0.74264 -2.58081
C  -1.29442  2.80159  1.07052
C  -2.53197  3.15415  0.53613
H  -2.70932  3.09502 -0.54004
C  -3.55967  3.58769  1.36377
H  -4.52344  3.85816  0.92115
C  -3.37114  3.68208  2.73542
H  -4.18673  4.02530  3.38069
C  -2.13866  3.36179  3.27472
H  -1.96065  3.46097  4.35109
C  -1.10729  2.93135  2.44936
H  -0.14077  2.69731  2.90114
C  -0.20271  2.99112 -1.55115
C  -0.50954  4.35909 -1.52020
H  -0.64792  4.86740 -0.56083
C  -0.64882  5.07652 -2.69271
H  -0.89029  6.14385 -2.64866
C  -0.48844  4.45010 -3.92092
H  -0.60284  5.02360 -4.84704
C  -0.18917  3.09898 -3.97210
H  -0.06856  2.59421 -4.93671
C  -0.04875  2.37734 -2.78996
H   0.16177  1.30447 -2.83152
C   1.51228  2.83294  0.77299
C   1.99086  2.30441  1.97636
H   1.54707  1.39191  2.37860
C   3.02770  2.91558  2.65915
H   3.38122  2.48330  3.60178
C   3.60913  4.06791  2.15872
H   4.42109  4.56048  2.70387
C   3.16854  4.57883  0.94766
H   3.63541  5.47482  0.52500
C   2.12600  3.96162  0.25337
H   1.80527  4.38255 -0.70244
C   2.86433 -2.10296 -0.99971
C   2.53743 -3.35399 -0.44879
H   1.98362 -3.40909  0.49508
C   2.94723 -4.52527 -1.06471
H   2.69590 -5.49103 -0.61360
C   3.68048 -4.47254 -2.23806
H   4.00862 -5.39886 -2.72135
C   4.00048 -3.25000 -2.79649
H   4.58308 -3.20172 -3.72246
C   3.59698 -2.06453 -2.18006
H   3.88862 -1.10769 -2.62167
C   3.31685 -1.00562  1.48865
C   2.75623 -0.93151  2.75851
H   1.73177 -0.56667  2.88442
C   3.47215 -1.33458  3.88299
H   3.00549 -1.28468  4.87217
C   4.76684 -1.79016  3.74031
H   5.33475 -2.10862  4.62117
C   5.35086 -1.85449  2.48438
H   6.37774 -2.21759  2.37212
C   4.63031 -1.47153  1.36005
H   5.09268 -1.55125  0.37111
C   3.35675  0.72513 -0.83196
C   4.42901  1.36151 -0.20535
H   4.73648  1.07192  0.80217
C   5.09938  2.39031 -0.85996
H   5.93462  2.88791 -0.35583
C   4.72734  2.78078 -2.13176
H   5.26705  3.58672 -2.64018
C   3.66202  2.15539 -2.75766
H   3.35475  2.46094 -3.76339
C   2.97662  1.13740 -2.11062
H   2.13587  0.64897 -2.61062
H  -0.01767  0.05964  1.65303
```

```
H   0.02891 -0.26364 -1.67713
H   0.07247 -2.03672  0.17201
```

13+ (H atoms optimized)

BP86 energy =   -2795.50443825

```
Ru  0.14883  0.16132 -0.11452
H  -0.16952 -0.98903 -1.63060
H   1.19120  0.24748 -2.12997
Zn -1.53491 -0.78913  1.30667
O   0.60977  1.49868  2.50610
N   1.70870 -2.15195  1.32527
N   3.12107 -0.91640  0.26719
N  -1.92423  2.51153 -0.48530
N  -2.52348  0.82644 -1.67703
C   1.78053 -1.05399  0.49395
C   3.86365 -1.86477  0.96476
H   4.94877 -1.88038  0.93177
C   2.98197 -2.64713  1.61263
H   3.12790 -3.51089  2.25484
C   3.74244  0.03933 -0.62225
C   4.14002  1.27618 -0.10955
C   4.77266  2.15515 -0.99221
H   5.10244  3.13348 -0.62874
C   4.99038  1.80473 -2.30452
H   5.47915  2.51569 -2.98031
C   4.63371  0.55920 -2.77471
H   4.85202  0.29313 -3.81287
C   4.02416 -0.37938 -1.93631
C   3.71886 -1.77651 -2.44937
H   2.84241 -2.16272 -1.89551
C   3.40763 -1.82700 -3.94978
H   4.31848 -1.68166 -4.55613
H   2.67981 -1.06285 -4.26402
H   3.00785 -2.81935 -4.22008
C   4.89721 -2.71743 -2.16466
H   5.81300 -2.35071 -2.66031
H   4.68883 -3.72934 -2.55376
H   5.11347 -2.81612 -1.09059
C   4.02470  1.62625  1.35414
H   3.34537  0.90241  1.83651
C   3.46945  3.01672  1.60773
H   4.14559  3.80664  1.23463
H   3.34693  3.18586  2.69075
H   2.48580  3.16374  1.13585
C   5.42561  1.45274  2.00312
H   6.15343  2.15234  1.55719
H   5.81713  0.43085  1.86591
H   5.37883  1.65749  3.08661
C   0.51780 -2.82102  1.80623
C  -0.84635 -3.48744  3.63637
H  -1.09576 -3.46281  4.70240
C  -1.61519 -4.23862  2.78012
H  -2.46803 -4.80522  3.16933
C  -1.31303 -4.28890  1.43454
H  -1.92211 -4.90370  0.76567
C  -0.21829 -3.59578  0.91949
C   0.25442 -2.74429  3.17926
C   1.09359 -1.93147  4.15180
H   1.80753 -1.31961  3.57480
C   0.23664 -0.99008  4.98710
H  -0.34870 -0.29863  4.35970
H   0.87394 -0.37845  5.64852
H  -0.46878 -1.53919  5.63453
C   1.89881 -2.86324  5.08374
H   1.22418 -3.48256  5.69980
H   2.52854 -2.27052  5.76864
H   2.55895 -3.55005  4.52736
C   0.19240 -3.76012 -0.53598
H   0.69046 -2.82379 -0.83970
C   1.19606 -4.87751 -0.67261
H   2.09649 -4.71339 -0.05673
H   1.52794 -4.98471 -1.72085
H   0.76114 -5.84643 -0.36740
C  -0.99376 -3.96742 -1.48510
H  -1.48381 -4.94447 -1.32840
H  -0.64544 -3.95461 -2.53070
H  -1.75778 -3.18133 -1.37196
C  -1.53990  1.23012 -0.78324
C  -3.05154  2.89883 -1.17380
H  -3.46958  3.89622 -1.07325
C  -3.43099  1.85319 -1.91021
H  -4.24831  1.73406 -2.61512
C  -1.21659  3.44114  0.35631
C  -1.66338  3.64482  1.67349
C  -1.01581  4.62329  2.40673
H  -1.33017  4.81909  3.43706
C   0.03496  5.37150  1.86716
H   0.52916  6.13620  2.47464
C   0.42440  5.15398  0.56369
H   1.22957  5.76317  0.13613
C  -0.19409  4.20393 -0.23156
C   0.18851  4.04088 -1.70520
H  -0.46630  3.26811 -2.14370
C  -0.06065  5.33738 -2.45924
H  -1.11032  5.66518 -2.37236
H   0.16627  5.21780 -3.53354
H   0.57293  6.15955 -2.08296
C   1.62078  3.54805 -1.82730
H   2.34002  4.26866 -1.40039
H   1.90120  3.39769 -2.88541
H   1.76137  2.59065 -1.29568
C  -2.83363  2.89490  2.25436
H  -3.02218  2.00881  1.61898
C  -4.07667  3.75835  2.24013
H  -4.94432  3.20987  2.64731
H  -4.34706  4.09844  1.22558
H  -3.93746  4.66237  2.85940
C  -2.57181  2.39890  3.66414
H  -1.64598  1.80547  3.73139
H  -3.40899  1.76900  4.00997
H  -2.48338  3.23182  4.38370
C  -2.68806 -0.43083 -2.37065
C  -3.83479 -1.20205 -2.11032
C  -4.04495 -2.32116 -2.92202
H  -4.93162 -2.93956 -2.74970
C  -3.17079 -2.66528 -3.92249
H  -3.36354 -3.54292 -4.54791
C  -2.04257 -1.88437 -4.15246
H  -1.35775 -2.16347 -4.95932
C  -1.78066 -0.76546 -3.39465
C  -4.81904 -0.90055 -1.00763
H  -4.40545 -0.10773 -0.35988
C  -6.17537 -0.39744 -1.59102
H  -6.06047  0.52232 -2.18704
H  -6.88767 -0.18589 -0.77583
```

```
H  -6.62601 -1.16440 -2.24366
C  -5.05573 -2.15018 -0.14681
H  -5.57999 -2.94118 -0.70902
H  -5.68500 -1.90166  0.72453
H  -4.10915 -2.57008  0.23036
C  -0.52335  0.04269 -3.55027
H  -0.74538  1.09742 -3.31348
C   0.12361 -0.01121 -4.94076
H   1.01119  0.64270 -4.97524
H  -0.57766  0.33560 -5.71772
H   0.45344 -1.02845 -5.21051
C   0.43958 -0.50227 -2.46698
H   1.00490 -1.37087 -2.83921
C   0.41362  1.03855  1.47785
C  -3.03919 -1.07401  2.51432
H  -3.88378 -0.42793  2.22111
H  -2.72676 -0.80417  3.53622
H  -3.36948 -2.12251  2.51037
```

14⁺ (H atoms optimized)

```
BP86 energy =     -2796.70924769

Ru  0.05345 -0.02299  0.21350
H  -0.81612  1.27560 -0.11540
H   0.35470 -0.41163 -1.46268
Zn -1.09855  0.53220 -1.91106
O  -0.22850  1.20175  2.96926
N  -2.81309 -1.21334  0.81732
N  -1.74161 -2.50145 -0.52683
N   2.97542  1.14146  0.64748
N   1.71957  2.56551 -0.35122
C  -0.15893  0.64429  1.97443
C  -2.47328  0.94679 -3.21484
H  -3.29212  0.20924 -3.18047
H  -2.05714  0.95841 -4.23454
H  -2.88493  1.94491 -2.99766
C  -1.61391 -1.32130  0.17480
C  -3.63532 -2.29422  0.54433
H  -4.62384 -2.38319  0.98481
C  -2.96995 -3.09201 -0.28707
H  -3.23671 -4.04870 -0.72603
C  -3.17759 -0.23363  1.82022
C  -3.88936  0.91289  1.43121
C  -4.27691  1.78669  2.43982
H  -4.82928  2.69529  2.18338
C  -3.98000  1.52478  3.76523
H  -4.29577  2.22989  4.54159
C  -3.29887  0.37880  4.11684
H  -3.08037  0.18498  5.17184
C  -2.89013 -0.53919  3.15764
C  -4.26507  1.15968 -0.03066
H  -3.48092  0.69939 -0.65949
C  -5.59265  0.49444 -0.37058
H  -5.58094 -0.59531 -0.20634
H  -5.85330  0.66502 -1.42975
H  -6.40908  0.91289  0.24446
C  -4.34622  2.63614 -0.39197
H  -5.17424  3.14430  0.13269
H  -4.54515  2.74711 -1.47128
H  -3.41426  3.17626 -0.16446
C  -2.18618 -1.83092  3.55453
H  -1.64321 -2.20104  2.66652
C  -3.20493 -2.88976  3.96264
H  -3.78019 -2.55898  4.84541
H  -2.70255 -3.83602  4.22983
H  -3.92943 -3.11313  3.16275
C  -1.16298 -1.65259  4.66988
H  -0.44582 -0.84568  4.45786
H  -0.59942 -2.58985  4.81937
H  -1.64411 -1.42050  5.63646
C  -0.72108 -3.20925 -1.27311
C   0.31796 -3.81681 -0.54914
C   1.23013 -4.58780 -1.26628
H   2.05127 -5.08179 -0.73891
C   1.09696 -4.75776 -2.63651
H   1.81991 -5.37804 -3.17711
C   0.06316 -4.16677 -3.31189
H  -0.03363 -4.32524 -4.39102
C  -0.88901 -3.38000 -2.65661
C   0.45265 -3.60560  0.93972
H  -0.55654 -3.42849  1.35082
C   1.05337 -4.79811  1.69656
H   0.47836 -5.72221  1.52010
H   1.04897 -4.59905  2.78154
H   2.10138 -4.98769  1.40951
C   1.31249 -2.36112  1.21285
H   2.38559 -2.60079  1.13741
H   1.12426 -1.92962  2.20462
H   1.21055 -1.59275  0.38149
C  -2.05552 -2.79719 -3.43553
H  -2.56591 -2.05480 -2.79599
C  -3.06453 -3.90083 -3.79111
H  -2.59855 -4.66323 -4.43929
H  -3.92632 -3.47952 -4.33683
H  -3.45501 -4.42302 -2.90211
C  -1.59755 -2.08787 -4.70658
H  -0.83464 -1.31929 -4.49825
H  -2.45103 -1.59205 -5.19863
H  -1.16844 -2.79304 -5.43893
C   1.70844  1.27324  0.12663
C   3.71291  2.30725  0.50577
H   4.73885  2.37539  0.85604
C   2.92962  3.19386 -0.10625
H   3.10440  4.22580 -0.39702
C   3.63154 -0.08491  1.04971
C   3.79123 -0.36143  2.41428
C   4.51471 -1.50859  2.74159
H   4.66918 -1.76206  3.79509
C   5.03980 -2.32467  1.76357
H   5.60555 -3.21755  2.05310
C   4.88102 -2.02069  0.43690
H   5.32228 -2.66823 -0.32824
C   4.17796 -0.88163  0.04006
C   3.26922  0.54961  3.50696
H   2.52924  1.23724  3.06198
C   2.57443 -0.23221  4.62919
H   1.79358 -0.90635  4.24184
H   2.09732  0.46396  5.33924
H   3.28839 -0.84374  5.20732
C   4.41446  1.38835  4.08432
H   5.19014  0.74280  4.53196
H   4.04407  2.06462  4.87404
H   4.90512  2.00955  3.31605
C   4.06016 -0.53204 -1.42601
H   3.33007  0.28880 -1.51545
C   3.52997 -1.67907 -2.26915
```

```
H   4.22219 -2.53972 -2.28289
H   3.40521 -1.35553 -3.31717
H   2.55231 -2.04062 -1.91139
C   5.38214 -0.02158 -1.97822
H   5.75598  0.85333 -1.41956
H   5.27537  0.27608 -3.03586
H   6.16552 -0.79931 -1.93318
C   0.68207  3.18637 -1.13510
C   0.68903  2.97736 -2.53091
C  -0.30575  3.60663 -3.26250
H  -0.33657  3.46527 -4.34765
C  -1.24965  4.42045 -2.66478
H  -2.01821  4.90573 -3.27522
C  -1.20113  4.63766 -1.30800
H  -1.92820  5.31175 -0.84225
C  -0.23153  4.03476 -0.50696
C   1.79122  2.18006 -3.21422
H   2.16658  1.44117 -2.48556
C   1.33769  1.40305 -4.44826
H   0.48528  0.73499 -4.23413
H   2.16547  0.77892 -4.82460
H   1.03867  2.06910 -5.27652
C   2.94877  3.10960 -3.58458
H   2.61854  3.87571 -4.30826
H   3.77169  2.54168 -4.05240
H   3.36368  3.63476 -2.70989
C  -0.15087  4.37710  0.97033
H   0.61339  3.72934  1.43414
C  -1.46617  4.14140  1.71194
H  -2.26522  4.81151  1.34834
H  -1.33683  4.34771  2.78796
H  -1.82052  3.10415  1.61303
C   0.30039  5.83371  1.12993
H   1.26785  6.02614  0.63513
H   0.41070  6.09246  2.19714
H  -0.43655  6.53033  0.69374
```

15^+ (H atoms optimized)

BP86 energy =   -2797.90221779

```
Ru -0.05055 -0.03658  0.20978
H  -0.61170  0.13249 -1.40400
H   0.96445  1.27023  0.14319
Zn  0.74984  1.35591 -1.70637
O   0.80407  0.12737  3.11728
N  -1.67304  2.60670  0.76122
N  -2.80166  0.89999  1.38811
N   1.35750 -2.28297 -1.45104
N   2.80301 -1.36309 -0.16470
C  -1.61756  1.23876  0.79352
C  -2.84254  3.08953  1.32716
H  -3.05614  4.15289  1.38390
C  -3.54881  2.01721  1.70904
H  -4.52106  1.93350  2.18611
C  -0.75081  3.47664  0.07567
C  -1.06010  3.85163 -1.23012
C  -0.16568  4.73104 -1.86445
H  -0.37498  5.05166 -2.89048
C   0.94705  5.19782 -1.22016
H   1.62365  5.88630 -1.73901
C   1.22241  4.82348  0.07863
H   2.10292  5.22506  0.58910
C   0.36632  3.95646  0.76293
C  -2.34062  3.41435 -1.93122
H  -2.89216  2.73770 -1.25688
C  -2.10464  2.68796 -3.22628
H  -1.55125  3.30554 -3.95570
H  -3.06567  2.41701 -3.69775
H  -1.53453  1.75378 -3.08644
C  -3.22526  4.63456 -2.18805
H  -3.44584  5.19290 -1.26219
H  -4.18794  4.33059 -2.63409
H  -2.74521  5.33878 -2.88955
C   0.59102  3.63095  2.21995
H  -0.00737  2.73960  2.47183
C   2.04598  3.32179  2.56206
H   2.45187  2.50420  1.94591
H   2.12907  3.01705  3.61866
H   2.69833  4.20316  2.43089
C   0.07544  4.79632  3.07014
H   0.63739  5.72196  2.85421
H   0.19130  4.57765  4.14597
H  -0.99173  5.00714  2.88468
C  -3.34962 -0.42985  1.50819
C  -3.99013 -0.96661  0.37869
C  -4.55436 -2.22869  0.51832
H  -5.05690 -2.68754 -0.33898
C  -4.51298 -2.89714  1.71792
H  -4.98200 -3.88332  1.80727
C  -3.89259 -2.33521  2.81696
H  -3.87306 -2.88037  3.76539
C  -3.28314 -1.08333  2.73074
C  -4.10929 -0.19095 -0.91504
H  -3.29426  0.55341 -0.93818
C  -3.94614 -1.05003 -2.15646
H  -3.00260 -1.61762 -2.14539
H  -3.94757 -0.41350 -3.05814
H  -4.77365 -1.77102 -2.28068
C  -5.43800  0.57486 -0.95140
H  -6.29441 -0.12224 -0.93287
H  -5.51719  1.17467 -1.87490
H  -5.54907  1.26045 -0.09520
C  -2.60788 -0.48551  3.94768
H  -1.95502  0.33644  3.60281
C  -1.72670 -1.49623  4.67367
H  -2.31959 -2.29079  5.15922
H  -1.15077 -0.99624  5.47068
H  -1.00900 -1.98145  3.99250
C  -3.65200  0.12039  4.89239
H  -3.16582  0.57471  5.77298
H  -4.35148 -0.65206  5.25772
H  -4.25249  0.90385  4.40069
C   1.47348 -1.30597 -0.49298
C   2.56559 -2.90917 -1.68848
H   2.65346 -3.72222 -2.40308
C   3.47320 -2.33228 -0.89418
H   4.53572 -2.51198 -0.76458
C   0.14366 -2.82153 -2.03501
C  -0.23759 -2.37760 -3.31186
C  -1.32481 -3.03961 -3.89005
H  -1.65949 -2.73615 -4.88689
C  -1.97380 -4.04869 -3.24617
H  -2.81899 -4.54695 -3.73486
C  -1.58264 -4.45379 -1.99482
H  -2.10687 -5.27384 -1.49406
C  -0.48659 -3.84927 -1.35925
```

```
C   0.48468 -1.27690 -4.03172
H   0.91199 -0.60733 -3.26002
C   1.66143 -1.79595 -4.86248
H   2.41345 -2.32258 -4.25414
H   2.17195 -0.95917 -5.36968
H   1.31173 -2.49531 -5.64275
C  -0.41928 -0.44160 -4.91070
H  -0.76066 -0.99969 -5.80068
H   0.12161  0.44317 -5.28433
H  -1.31783 -0.09188 -4.37529
C   0.01055 -4.40139 -0.03906
H   0.73481 -3.68216  0.38417
C  -1.07357 -4.61014  1.00147
H  -1.64983 -3.69211  1.20475
H  -0.62594 -4.94512  1.95288
H  -1.79574 -5.38919  0.69953
C   0.77779 -5.71888 -0.28899
H   0.10812 -6.48764 -0.71246
H   1.18964 -6.11437  0.65536
H   1.61543 -5.58196 -0.99222
C   3.48377 -0.63432  0.88309
C   3.50883 -1.20216  2.16601
C   4.23311 -0.52865  3.14002
H   4.27428 -0.93144  4.15593
C   4.90236  0.64685  2.84885
H   5.46275  1.16071  3.63755
C   4.88333  1.16371  1.57528
H   5.44068  2.07954  1.35804
C   4.18987  0.52351  0.54685
C   4.27432  1.04427 -0.87216
H   3.39739  0.66827 -1.43240
C   5.53493  0.50080 -1.56467
H   6.44458  0.83605 -1.03622
H   5.59572  0.86622 -2.60443
H   5.55601 -0.60039 -1.59612
C   4.25173  2.57188 -0.94563
H   3.37001  2.99852 -0.43976
H   4.23094  2.89867 -1.99809
H   5.15350  3.01919 -0.49321
C   2.83751 -2.53072  2.48729
H   1.95201 -2.62798  1.83088
C   3.78253 -3.70055  2.18193
H   4.08971 -3.73968  1.12610
H   3.29667 -4.66248  2.42183
H   4.69718 -3.62831  2.79641
C   2.35516 -2.64116  3.93179
H   3.19815 -2.72402  4.64018
H   1.75512 -3.55895  4.05548
H   1.73776 -1.78412  4.23913
C   0.50952 -0.02094  2.03292
C   1.61579  2.15102 -3.24502
H   0.87324  2.34426 -4.03571
H   2.39321  1.48385 -3.65420
H   2.08257  3.10860 -2.96966
H  -1.42514 -1.13244  0.28431
H  -0.73672 -1.64122  0.33990
```

ZnMeH

```
BP86 energy =   -267.732063359
Enthalpy 0K =   -267.691450
Enthalpy 298K = -267.686684
Free energy 298K = -267.716458
Low Freq. = 435.2689 cm-1

Zn  0.42778  0.00009  0.00006
C  -1.51237 -0.00012 -0.00008
H  -1.90779  0.40518 -0.94742
H  -1.90798  0.61754  0.82457
H  -1.90765 -1.02325  0.12248
H   1.96410 -0.00156 -0.00109
```

Functional screening

2_bp86

```
BP86 energy =     -2732.72996875
Enthalpy 0K =     -2731.880442
Enthalpy 298K =   -2731.816941
Free energy 298K =      -2731.982300
Low Freq. = 13.6828 cm-1

Ru  0.02231 -0.36429  0.22856
Zn  0.11304 -0.24706 -2.29095
Zn  0.09288 -2.54821 -1.05624
P   2.38836 -0.84115  0.38409
P   0.25154  2.06900  0.03744
P  -2.36541 -0.50447  0.10759
C   0.30543  0.27569 -4.19790
H   0.03076  1.33037 -4.37944
H  -0.36664 -0.35432 -4.80787
H   1.33794  0.11603 -4.55589
C   0.23578 -4.37511 -1.78847
H   1.23745 -4.52323 -2.22694
H  -0.51196 -4.53734 -2.58537
H   0.08156 -5.13303 -1.00156
C   0.43248 -2.14992  1.35244
C  -0.29620 -3.08348  2.13185
H  -1.38713 -3.12517  2.07603
C   0.36877 -3.96595  3.00780
H  -0.22279 -4.67570  3.59856
C   1.76734 -3.94556  3.15484
H   2.25920 -4.62954  3.85470
C   2.52929 -3.03680  2.39733
H   3.61826 -2.98294  2.51101
C   1.85443 -2.19194  1.50501
C   3.29019 -1.65420 -1.03626
C   3.75393 -2.98297 -0.94416
H   3.58654 -3.55509 -0.02674
C   4.40949 -3.58277 -2.03274
H   4.75503 -4.61881 -1.95066
C   4.61895 -2.86158 -3.21846
H   5.12866 -3.33180 -4.06587
C   4.16829 -1.53424 -3.31511
H   4.32736 -0.96447 -4.23672
C   3.50222 -0.93573 -2.23420
H   3.15208  0.09867 -2.31836
C   3.76523  0.10116  1.23090
C   3.58888  0.51893  2.56940
H   2.65671  0.29227  3.09397
C   4.60571  1.22392  3.23087
H   4.45430  1.53551  4.26989
C   5.80722  1.52632  2.56916
H   6.60083  2.07348  3.08935
C   5.98624  1.11804  1.23862
H   6.92059  1.34394  0.71351
C   4.97312  0.41052  0.57171
H   5.13241  0.09169 -0.46218
```

```
C   0.14442  0.63136  2.11519
C   0.15691  0.22994  3.47467
H   0.09173 -0.83399  3.73410
C   0.23749  1.18746  4.50537
H   0.23856  0.85483  5.55115
C   0.30634  2.56669  4.22271
H   0.35395  3.29494  5.03961
C   0.31327  3.00230  2.88587
H   0.35285  4.07170  2.64658
C   0.25065  2.02918  1.87216
C  -0.92779  3.44060 -0.47425
C  -2.02125  3.76300  0.35841
H  -2.15642  3.24030  1.30977
C  -2.93598  4.75766 -0.02330
H  -3.77498  4.99544  0.63869
C  -2.77856  5.43862 -1.24045
H  -3.49292  6.21463 -1.53517
C  -1.69450  5.12283 -2.07551
H  -1.55525  5.65468 -3.02304
C  -0.77489  4.13247 -1.69624
H   0.07758  3.91565 -2.34741
C   1.87452  2.79800 -0.53899
C   2.27329  2.63374 -1.88413
H   1.65324  2.04641 -2.57329
C   3.46289  3.21672 -2.35236
H   3.75308  3.08906 -3.40090
C   4.27702  3.95438 -1.47789
H   5.20714  4.40446 -1.84062
C   3.89727  4.10362 -0.13469
H   4.53453  4.66363  0.55756
C   2.70217  3.53327  0.33282
H   2.42132  3.64433  1.38399
C  -3.45256  0.62556  1.16373
C  -3.11374  0.82665  2.51996
H  -2.21370  0.36088  2.92805
C  -3.91014  1.63653  3.34682
H  -3.62359  1.78173  4.39376
C  -5.05746  2.26102  2.83140
H  -5.67674  2.89594  3.47422
C  -5.40588  2.06270  1.48583
H  -6.30150  2.53977  1.07297
C  -4.61229  1.25121  0.65831
H  -4.90160  1.10884 -0.38614
C  -3.16500 -0.18744 -1.57639
C  -2.73447  0.93379 -2.31937
H  -1.95506  1.58692 -1.91633
C  -3.32022  1.25133 -3.55503
H  -2.97021  2.13106 -4.10447
C  -4.34258  0.44478 -4.07952
H  -4.79230  0.68324 -5.04910
C  -4.78892 -0.66481 -3.34570
H  -5.59440 -1.29581 -3.73698
C  -4.21510 -0.97242 -2.09983
H  -4.59587 -1.82737 -1.53536
C  -3.14496 -2.16843  0.53765
C  -2.91347 -3.28032 -0.30304
H  -2.34602 -3.15919 -1.23284
C  -3.41669 -4.54995  0.01950
H  -3.22018 -5.39311 -0.65067
C  -4.16356 -4.73376  1.19452
H  -4.55297 -5.72441  1.45152
C  -4.41188 -3.63571  2.03203
H  -5.00171 -3.76294  2.94625
C  -3.91194 -2.36296  1.70521
H  -4.12151 -1.51926  2.36823
```

2_blyp

```
BLYP energy =     -2731.09924341
Enthalpy 0K =     -2730.250159
Enthalpy 298K =   -2730.186417
Free energy 298K =      -2730.353124
Low Freq. = 12.5020 cm-1

Ru  0.03280 -0.38393  0.21044
Zn  0.13267 -0.26278 -2.35142
Zn  0.08773 -2.57715 -1.14539
P   2.44718 -0.86832  0.38103
P   0.25314  2.12781  0.02742
P  -2.41915 -0.52586  0.11759
C   0.32963  0.31406 -4.26078
H   0.03252  1.36469 -4.41853
H  -0.31889 -0.31772 -4.89157
H   1.36875  0.18822 -4.60801
C   0.22709 -4.43212 -1.85705
H   1.22075 -4.58406 -2.30853
H  -0.53267 -4.60484 -2.63825
H   0.08677 -5.17899 -1.05915
C   0.46048 -2.18950  1.32906
C  -0.26817 -3.13237  2.10039
H  -1.35706 -3.16066  2.06097
C   0.39713 -4.04752  2.94491
H  -0.19429 -4.76266  3.52609
C   1.80009 -4.05137  3.06631
H   2.29476 -4.76017  3.73608
C   2.56055 -3.12977  2.32016
H   3.64976 -3.09666  2.41902
C   1.88516 -2.24622  1.46108
C   3.38742 -1.63245 -1.05390
C   3.86389 -2.96177 -1.00072
H   3.68635 -3.56967 -0.11205
C   4.55127 -3.51763 -2.09555
H   4.90533 -4.55076 -2.04081
C   4.78149 -2.75294 -3.25203
H   5.31504 -3.18737 -4.10158
C   4.31882 -1.42592 -3.31168
H   4.49304 -0.82260 -4.20678
C   3.62119 -0.87144 -2.22421
H   3.26466  0.15913 -2.28535
C   3.81510  0.04216  1.29847
C   3.66273  0.31088  2.68000
H   2.76278 -0.01266  3.20256
C   4.66498  0.99499  3.38856
H   4.52965  1.18894  4.45621
C   5.83247  1.42477  2.73316
H   6.61291  1.95355  3.28731
C   5.99053  1.16504  1.36136
H   6.89571  1.48945  0.84042
C   4.99076  0.48045  0.64799
H   5.13941  0.28088 -0.41398
C   0.15667  0.63821  2.11044
C   0.16786  0.23788  3.47215
H   0.10344 -0.82312  3.73274
C   0.24305  1.19327  4.50741
H   0.24255  0.85890  5.55081
C   0.30800  2.57403  4.22511
H   0.35225  3.30202  5.04007
```

```
C   0.31462  3.00860  2.88653
H   0.34949  4.07702  2.65195
C   0.25604  2.03792  1.86540
C  -0.94641  3.50827 -0.45086
C  -2.02360  3.83569  0.40507
H  -2.13697  3.32260  1.36063
C  -2.95170  4.82727  0.03974
H  -3.77377  5.06669  0.71941
C  -2.82612  5.50277 -1.18605
H  -3.54837  6.27382 -1.46765
C  -1.76014  5.18331 -2.04578
H  -1.64561  5.70768 -2.99878
C  -0.82825  4.19571 -1.68253
H   0.00307  3.97871 -2.35600
C   1.87675  2.89454 -0.53398
C   2.28702  2.75051 -1.88078
H   1.68460  2.16176 -2.57771
C   3.46842  3.36085 -2.34096
H   3.76395  3.24632 -3.38772
C   4.26582  4.11000 -1.45802
H   5.18611  4.58130 -1.81351
C   3.87594  4.24270 -0.11409
H   4.49558  4.81307  0.58320
C   2.68979  3.64271  0.34538
H   2.40641  3.74668  1.39365
C  -3.50177  0.59865  1.20490
C  -3.15135  0.78923  2.56207
H  -2.24973  0.32619  2.95944
C  -3.94583  1.58390  3.40872
H  -3.64900  1.71791  4.45249
C  -5.10554  2.20589  2.91429
H  -5.72189  2.82661  3.57056
C  -5.46687  2.02009  1.56865
H  -6.36945  2.49323  1.17138
C  -4.67503  1.22344  0.72186
H  -4.98065  1.09242 -0.31621
C  -3.23577 -0.18510 -1.56612
C  -2.81165  0.94601 -2.30249
H  -2.02666  1.58978 -1.90715
C  -3.40963  1.28090 -3.53008
H  -3.06336  2.16532 -4.07085
C  -4.44064  0.48320 -4.05591
H  -4.89929  0.73507 -5.01584
C  -4.88148 -0.63609 -3.33054
H  -5.69121 -1.25966 -3.71998
C  -4.29440 -0.96125 -2.09297
H  -4.67440 -1.82102 -1.54148
C  -3.19655 -2.20620  0.54009
C  -2.98825 -3.31069 -0.32074
H  -2.43639 -3.18378 -1.25447
C  -3.49980 -4.58236 -0.01069
H  -3.32326 -5.41442 -0.69725
C  -4.23082 -4.77996  1.17404
H  -4.62584 -5.76922  1.42000
C  -4.45332 -3.69221  2.03451
H  -5.02787 -3.82795  2.95511
C  -3.94623 -2.41709  1.71942
H  -4.14010 -1.58883  2.40124
```

2_b3lyp

```
B3LYP energy =     -2732.41356713
Enthalpy 0K =      -2731.537820
Enthalpy 298K =    -2731.475939
Free energy 298K =       -2731.638499
Low Freq. = 12.9369 cm-1

Ru  0.03395 -0.37794  0.20277
Zn  0.12659 -0.26352 -2.33609
Zn  0.08955 -2.56370 -1.11049
P   2.42287 -0.84732  0.37692
P   0.24981  2.09696  0.03028
P  -2.40223 -0.51349  0.11930
C   0.32484  0.37217 -4.21383
H   0.01091  1.41528 -4.34232
H  -0.30239 -0.24354 -4.86996
H   1.36063  0.28058 -4.56177
C   0.20772 -4.41089 -1.81209
H   1.19491 -4.58040 -2.25585
H  -0.54350 -4.58158 -2.59287
H   0.05554 -5.15075 -1.01888
C   0.46313 -2.18175  1.31239
C  -0.24718 -3.13019  2.07569
H  -1.32760 -3.18749  2.02054
C   0.42176 -4.01166  2.93571
H  -0.15698 -4.73011  3.51212
C   1.81224 -3.97905  3.07895
H   2.30974 -4.66224  3.76165
C   2.55545 -3.05685  2.33639
H   3.63548 -2.99693  2.44708
C   1.87599 -2.20782  1.46244
C   3.34170 -1.62350 -1.04415
C   3.81704 -2.94120 -0.97735
H   3.65337 -3.53151 -0.08248
C   4.48381 -3.51025 -2.06548
H   4.83715 -4.53546 -2.00036
C   4.69300 -2.76965 -3.22912
H   5.20986 -3.21461 -4.07449
C   4.23072 -1.45363 -3.30307
H   4.38770 -0.86916 -4.20518
C   3.55485 -0.88710 -2.22158
H   3.19819  0.13572 -2.29311
C   3.77829  0.08003  1.25826
C   3.61620  0.40857  2.61487
H   2.71411  0.11743  3.13900
C   4.60965  1.11074  3.29747
H   4.46642  1.35215  4.34700
C   5.77845  1.49873  2.63954
H   6.55265  2.04256  3.17373
C   5.94720  1.17781  1.29262
H   6.85420  1.46958  0.77046
C   4.95582  0.47429  0.60603
H   5.11177  0.22806 -0.43794
C   0.15074  0.62328  2.09223
C   0.16313  0.22269  3.44376
H   0.10855 -0.83290  3.69995
C   0.22844  1.16970  4.47272
H   0.22906  0.83589  5.50878
C   0.28308  2.54258  4.19592
H   0.32003  3.26370  5.00786
C   0.29045  2.97638  2.86859
H   0.32075  4.03845  2.63641
C   0.24134  2.01328  1.85364
C  -0.94231  3.45180 -0.46489
C  -2.01807  3.77894  0.37512
H  -2.13405  3.27744  1.32906
C  -2.94451  4.75276 -0.00380
```

```
H  -3.76777  4.99097  0.66331
C  -2.81663  5.41076 -1.22714
H  -3.53796  6.16846 -1.51983
C  -1.75058  5.09237 -2.07083
H  -1.63483  5.60403 -3.02241
C  -0.82118  4.12238 -1.69356
H   0.00991  3.90450 -2.35582
C   1.86491  2.84793 -0.51792
C   2.27826  2.70779 -1.85309
H   1.67731  2.13414 -2.55347
C   3.46095  3.30168 -2.29968
H   3.75967  3.18987 -3.33841
C   4.25614  4.03079 -1.41391
H   5.17852  4.48939 -1.75884
C   3.86283  4.15989 -0.08132
H   4.48208  4.71337  0.61869
C   2.67546  3.57558  0.36413
H   2.39043  3.67341  1.40540
C  -3.46654  0.60891  1.19209
C  -3.11720  0.80022  2.53863
H  -2.22758  0.33010  2.93628
C  -3.89487  1.60390  3.37514
H  -3.59775  1.73892  4.41124
C  -5.03752  2.23453  2.88011
H  -5.64146  2.86331  3.52850
C  -5.39881  2.04742  1.54522
H  -6.28886  2.52771  1.14785
C  -4.62282  1.24168  0.70908
H  -4.92644  1.11269 -0.32267
C  -3.21455 -0.18968 -1.54754
C  -2.78688  0.91915 -2.29512
H  -1.99700  1.55512 -1.91542
C  -3.38496  1.24356 -3.51375
H  -3.03365  2.11057 -4.06509
C  -4.42223  0.45741 -4.01717
H  -4.88247  0.70100 -4.97044
C  -4.86762 -0.63967 -3.27983
H  -5.68250 -1.25390 -3.65306
C  -4.27807 -0.95462 -2.05251
H  -4.66004 -1.79827 -1.49079
C  -3.15768 -2.17902  0.55090
C  -2.94934 -3.27645 -0.30169
H  -2.41697 -3.14849 -1.23878
C  -3.43639 -4.54359  0.01873
H  -3.25746 -5.37178 -0.66058
C  -4.14474 -4.74227  1.20544
H  -4.52102 -5.72883  1.46024
C  -4.37009 -3.66057  2.05614
H  -4.92842 -3.79792  2.97804
C  -3.88572 -2.39018  1.73073
H  -4.07981 -1.56573  2.40626
```

2_pbe

```
PBE energy =       -2729.60357164
Enthalpy 0K =      -2728.751126
Enthalpy 298K =    -2728.687837
Free energy 298K =       -2728.852166
Low Freq. = 13.9864 cm-1

Ru  0.02276 -0.36069  0.23777
Zn  0.10153 -0.24936 -2.28109
Zn  0.09534 -2.55316 -1.02793
P   2.37616 -0.83774  0.38269
P   0.25483  2.05815  0.04030
P  -2.35714 -0.49918  0.11238
C   0.28630  0.28496 -4.18427
H   0.02438  1.34467 -4.35101
H  -0.40029 -0.32790 -4.79398
H   1.31282  0.11491 -4.55239
C   0.23251 -4.38563 -1.74300
H   1.23010 -4.54059 -2.18633
H  -0.52145 -4.55633 -2.53103
H   0.08219 -5.13232 -0.94551
C   0.43336 -2.14005  1.36593
C  -0.29171 -3.07160  2.14829
H  -1.38162 -3.11932  2.09215
C   0.37601 -3.94413  3.02920
H  -0.21254 -4.65286  3.62269
C   1.77261 -3.91574  3.17890
H   2.26571 -4.59219  3.88387
C   2.53121 -3.01039  2.41713
H   3.61930 -2.95108  2.53011
C   1.85366 -2.17674  1.51951
C   3.25953 -1.66650 -1.03734
C   3.71290 -2.99648 -0.93942
H   3.55827 -3.55743 -0.01359
C   4.33918 -3.61304 -2.03374
H   4.67602 -4.65082 -1.94796
C   4.52955 -2.90772 -3.23013
H   5.01543 -3.39203 -4.08252
C   4.09107 -1.57835 -3.33152
H   4.23545 -1.02019 -4.26184
C   3.45438 -0.96286 -2.24499
H   3.11195  0.07295 -2.33310
C   3.75300  0.12211  1.20118
C   3.58339  0.55986  2.53231
H   2.66154  0.32685  3.07079
C   4.59272  1.29514  3.16794
H   4.44620  1.62427  4.20150
C   5.77970  1.60636  2.48774
H   6.56758  2.17841  2.98773
C   5.95279  1.17449  1.16559
H   6.87655  1.40715  0.62655
C   4.94702  0.43728  0.52386
H   5.10032  0.10277 -0.50540
C   0.14736  0.63554  2.11759
C   0.16351  0.23635  3.47603
H   0.10347 -0.82666  3.73785
C   0.24102  1.19556  4.50290
H   0.24480  0.86542  5.54858
C   0.30331  2.57316  4.21825
H   0.34820  3.30214  5.03350
C   0.30816  3.00635  2.88242
H   0.34469  4.07452  2.64023
C   0.24892  2.03160  1.87289
C  -0.92373  3.41869 -0.49035
C  -2.01601  3.75032  0.33691
H  -2.14335  3.25044  1.30080
C  -2.94288  4.72182 -0.06816
H  -3.78366  4.96460  0.58875
C  -2.79739  5.37057 -1.30223
H  -3.52237  6.12780 -1.61610
C  -1.71179  5.04792 -2.13006
H  -1.58146  5.55638 -3.09070
C  -0.78024  4.08041 -1.72817
H   0.07195  3.85597 -2.37615
```

```
C   1.87982  2.77905 -0.53346
C   2.28336  2.60607 -1.87409
H   1.66064  2.02491 -2.56497
C   3.48269  3.16978 -2.33559
H   3.77775  3.03419 -3.38107
C   4.30101  3.89669 -1.45878
H   5.24006  4.33039 -1.81599
C   3.91471  4.05609 -0.12043
H   4.55639  4.60589  0.57476
C   2.70997  3.50541  0.34056
H   2.42573  3.62168  1.38960
C  -3.43707  0.63783  1.16288
C  -3.09634  0.83664  2.51715
H  -2.20784  0.35335  2.92816
C  -3.87278  1.66905  3.33728
H  -3.58251  1.81477  4.38247
C  -5.00291  2.31687  2.81697
H  -5.60639  2.97093  3.45419
C  -5.35544  2.11724  1.47437
H  -6.23895  2.61208  1.05839
C  -4.58098  1.28420  0.65293
H  -4.87136  1.14401 -0.39098
C  -3.15454 -0.18714 -1.57001
C  -2.72160  0.93161 -2.31161
H  -1.94314  1.58324 -1.90675
C  -3.30289  1.24886 -3.54731
H  -2.95055  2.12805 -4.09517
C  -4.32324  0.44330 -4.07270
H  -4.77015  0.68058 -5.04294
C  -4.77240 -0.66357 -3.33937
H  -5.57724 -1.29314 -3.73197
C  -4.20346 -0.97048 -2.09301
H  -4.58727 -1.82314 -1.52811
C  -3.13237 -2.15951  0.54763
C  -2.89893 -3.27016 -0.29117
H  -2.34495 -3.14579 -1.22792
C  -3.38283 -4.54253  0.04215
H  -3.18270 -5.38593 -0.62560
C  -4.11389 -4.72895  1.22465
H  -4.48784 -5.72223  1.49080
C  -4.36810 -3.63079  2.05763
H  -4.94794 -3.76058  2.97688
C  -3.88611 -2.35552  1.72126
H  -4.09805 -1.51138  2.38215
```

2_pbe0

```
PBE0 energy =     -2729.70162679
Enthalpy 0K =     -2728.819107
Enthalpy 298K =   -2728.757884
Free energy 298K =      -2728.917643
Low Freq. = 14.6168 cm-1

Ru  0.02528 -0.35932  0.21915
Zn  0.08822 -0.24221 -2.28320
Zn  0.10424 -2.54489 -1.02094
P   2.36248 -0.81764  0.37421
P   0.24773  2.04024  0.04279
P  -2.34570 -0.49413  0.11466
C   0.26896  0.36497 -4.15840
H  -0.01675  1.41609 -4.29023
H  -0.39242 -0.23389 -4.79610
H   1.29243  0.23948 -4.53197
C   0.22923 -4.36869 -1.74342
H   1.21405 -4.52796 -2.19607
H  -0.52587 -4.54064 -2.52006
H   0.08835 -5.11659 -0.95540
C   0.44172 -2.14213  1.33302
C  -0.26380 -3.08628  2.10006
H  -1.34316 -3.16456  2.02812
C   0.40673 -3.93120  2.98937
H  -0.16819 -4.64770  3.57197
C   1.78956 -3.86643  3.16000
H   2.28484 -4.52166  3.87116
C   2.53002 -2.95399  2.40877
H   3.60702 -2.86826  2.53410
C   1.84950 -2.14783  1.50342
C   3.23946 -1.64476 -1.03212
C   3.70940 -2.95684 -0.92400
H   3.57014 -3.50664  0.00158
C   4.33268 -3.57251 -2.00803
H   4.68236 -4.59676 -1.91415
C   4.50181 -2.88337 -3.20494
H   4.98462 -3.36678 -4.04938
C   4.04472 -1.57167 -3.31813
H   4.17082 -1.02706 -4.24965
C   3.41234 -0.95856 -2.24110
H   3.05588  0.06341 -2.33811
C   3.72475  0.14648  1.17692
C   3.55967  0.58951  2.49580
H   2.64636  0.36039  3.03458
C   4.56241  1.32431  3.12030
H   4.41945  1.65741  4.14456
C   5.73914  1.62929  2.44002
H   6.52227  2.20051  2.93106
C   5.90846  1.19219  1.12995
H   6.82407  1.42045  0.59142
C   4.90858  0.45520  0.50040
H   5.05946  0.11651 -0.51937
C   0.15108  0.61354  2.09335
C   0.17612  0.20863  3.44013
H   0.12848 -0.84887  3.69354
C   0.24798  1.15439  4.46503
H   0.25876  0.81998  5.50086
C   0.29680  2.52537  4.19206
H   0.33767  3.24332  5.00665
C   0.29468  2.96335  2.86956
H   0.32278  4.02550  2.63552
C   0.24103  2.00109  1.86088
C  -0.92973  3.38144 -0.48116
C  -2.01595  3.70287  0.34010
H  -2.14248  3.20161  1.29468
C  -2.93985  4.66573 -0.05863
H  -3.77627  4.90070  0.59324
C  -2.79592  5.31590 -1.28047
H  -3.51799  6.06649 -1.58936
C  -1.71597  5.00356 -2.10277
H  -1.58778  5.51304 -3.05395
C  -0.78817  4.04484 -1.70648
H   0.05849  3.82797 -2.35075
C   1.85890  2.76034 -0.52116
C   2.26311  2.59584 -1.85159
H   1.64723  2.02390 -2.54363
C   3.45353  3.15966 -2.30498
H   3.74902  3.03029 -3.34271
C   4.26189  3.88009 -1.42965
H   5.19370  4.31469 -1.78037
```

```
C   3.87503  4.03206 -0.10123
H   4.50904  4.57655  0.59248
C   2.67970  3.47931  0.35128
H   2.39665  3.58843  1.39302
C  -3.40898  0.62332  1.17238
C  -3.06088  0.81339  2.51522
H  -2.17633  0.33098  2.91440
C  -3.82563  1.63377  3.34108
H  -3.52916  1.77236  4.37702
C  -4.95238  2.27875  2.83714
H  -5.54719  2.92343  3.47846
C  -5.31279  2.08842  1.50590
H  -6.19330  2.58160  1.10290
C  -4.54916  1.26726  0.67937
H  -4.84501  1.13689 -0.35591
C  -3.15289 -0.18084 -1.54353
C  -2.72440  0.92285 -2.28994
H  -1.93751  1.56254 -1.90398
C  -3.31822  1.24118 -3.50796
H  -2.96662  2.10690 -4.06154
C  -4.35049  0.45274 -4.00825
H  -4.80865  0.69118 -4.96389
C  -4.79658 -0.63904 -3.26935
H  -5.61007 -1.25491 -3.64301
C  -4.21193 -0.94801 -2.04245
H  -4.59203 -1.78929 -1.47323
C  -3.09447 -2.14942  0.54196
C  -2.86380 -3.24208 -0.30380
H  -2.33357 -3.10512 -1.24319
C  -3.32442 -4.51328  0.02333
H  -3.12478 -5.34297 -0.64855
C  -4.03130 -4.71566  1.20638
H  -4.38727 -5.70799  1.46802
C  -4.28529 -3.63497  2.04502
H  -4.84686 -3.77776  2.96415
C  -3.82483 -2.36084  1.71482
H  -4.03530 -1.53095  2.38099
```

2_b97d

```
B97D energy =     -2732.21063623
Enthalpy 0K =     -2731.354504
Enthalpy 298K =   -2731.292422
Free energy 298K =      -2731.451534
Low Freq. = 17.1341 cm-1

Ru  0.01467 -0.38309  0.24056
Zn  0.21002 -0.30646 -2.29038
Zn  0.02014 -2.61099 -1.02481
P   2.35895 -0.81135  0.39901
P   0.22712  2.01774  0.04747
P  -2.33119 -0.46365  0.14163
C   0.45283  0.39910 -4.15376
H   0.09382  1.43956 -4.23138
H  -0.13169 -0.21093 -4.86235
H   1.51004  0.36906 -4.46331
C   0.04261 -4.51459 -1.61316
H   0.87740 -4.68615 -2.31088
H  -0.89681 -4.78080 -2.12585
H   0.16512 -5.18193 -0.74467
C   0.45000 -2.15576  1.37831
C  -0.25716 -3.10070  2.15570
H  -1.33691 -3.19089  2.08349
C   0.42693 -3.94172  3.05654
H  -0.14517 -4.66202  3.64641
C   1.82147 -3.86720  3.22475
H   2.32446 -4.52024  3.94002
C   2.56264 -2.94636  2.46140
H   3.64488 -2.85581  2.57569
C   1.86634 -2.14221  1.55346
C   3.14632 -1.65430 -1.05739
C   3.35481 -3.04751 -1.05780
H   3.14283 -3.62722 -0.16008
C   3.80582 -3.69245 -2.22156
H   3.94876 -4.77402 -2.21468
C   4.06191 -2.95438 -3.38681
H   4.40526 -3.45906 -4.29096
C   3.87119 -1.56157 -3.38605
H   4.06668 -0.97970 -4.28808
C   3.41003 -0.91534 -2.23134
H   3.24869  0.16170 -2.24034
C   3.71085  0.20672  1.15794
C   3.49759  0.75918  2.43983
H   2.56614  0.56938  2.96741
C   4.48425  1.55644  3.03498
H   4.30563  1.97616  4.02632
C   5.68920  1.81534  2.36105
H   6.45610  2.43706  2.82632
C   5.90300  1.26844  1.08702
H   6.83547  1.46408  0.55509
C   4.92023  0.46751  0.48589
H   5.09833  0.04795 -0.50288
C   0.11300  0.60885  2.11436
C   0.14158  0.18880  3.46306
H   0.11798 -0.87528  3.70595
C   0.18524  1.14278  4.49784
H   0.19620  0.80759  5.53848
C   0.20814  2.52624  4.22339
H   0.23000  3.24585  5.04377
C   0.20983  2.97413  2.89062
H   0.23015  4.04045  2.65528
C   0.17688  2.00565  1.87609
C  -0.92855  3.34432 -0.56950
C  -2.01586  3.75068  0.22812
H  -2.14204  3.34123  1.22878
C  -2.94688  4.67504 -0.27053
H  -3.78483  4.97564  0.35887
C  -2.81032  5.19228 -1.56727
H  -3.53986  5.90601 -1.95335
C  -1.72647  4.79037 -2.36644
H  -1.60684  5.19366 -3.37359
C  -0.78872  3.87417 -1.87062
H   0.05479  3.57742 -2.49338
C   1.88433  2.68915 -0.47065
C   2.32623  2.50067 -1.79590
H   1.70386  1.96152 -2.51104
C   3.57642  2.99092 -2.20426
H   3.90452  2.83950 -3.23424
C   4.40200  3.66130 -1.28824
H   5.37959  4.03155 -1.60084
C   3.97066  3.84080  0.03499
H   4.61628  4.33825  0.75890
C   2.71726  3.36109  0.44296
H   2.39888  3.48226  1.47664
C  -3.34943  0.80621  1.09144
C  -3.02212  1.08245  2.43558
H  -2.20630  0.55248  2.91533
```

```
C  -3.73053  2.05055  3.16268
H  -3.44545  2.25593  4.19542
C  -4.78578  2.75384  2.56000
H  -5.33357  3.51247  3.12174
C  -5.13382  2.46912  1.23084
H  -5.95583  3.00393  0.75206
C  -4.42115  1.50573  0.50062
H  -4.69723  1.31428 -0.53427
C  -3.09669 -0.25899 -1.56330
C  -2.63434  0.80953 -2.35869
H  -1.85494  1.46552 -1.98143
C  -3.18373  1.06001 -3.62299
H  -2.80538  1.89537 -4.21308
C  -4.20848  0.23799 -4.11865
H  -4.63056  0.42016 -5.10817
C  -4.69452 -0.81293 -3.32580
H  -5.50307 -1.44747 -3.69299
C  -4.15537 -1.05084 -2.04988
H  -4.56827 -1.85043 -1.43789
C  -3.10610 -2.06501  0.72641
C  -2.94840 -3.23054 -0.05333
H  -2.52077 -3.16704 -1.05330
C  -3.34898 -4.48260  0.43234
H  -3.20300 -5.36889 -0.18630
C  -3.92407 -4.58995  1.70885
H  -4.22740 -5.56433  2.09460
C  -4.11416 -3.43318  2.48035
H  -4.57363 -3.50249  3.46778
C  -3.71214 -2.17879  1.99254
H  -3.86047 -1.29351  2.60813
```

2_b97d3

```
B97D3 energy =    -2732.46637208
Enthalpy 0K =     -2731.606664
Enthalpy 298K =   -2731.544419
Free energy 298K =       -2731.704609
Low Freq. = 16.1221 cm-1
```

```
Ru  0.01661 -0.39001  0.23457
Zn  0.10289 -0.20023 -2.27818
Zn  0.05496 -2.53503 -1.12055
P   2.35133 -0.83406  0.38394
P   0.26184  2.00781  0.03984
P  -2.33132 -0.47685  0.14961
C   0.28438  0.51574 -4.13204
H  -0.00519  1.57622 -4.18210
H  -0.38596 -0.04523 -4.79995
H   1.31288  0.41244 -4.50857
C   0.15397 -4.41329 -1.74855
H   1.04468 -4.55054 -2.37695
H  -0.73288 -4.68483 -2.34059
H   0.22072 -5.09438 -0.88783
C   0.43012 -2.15781  1.37259
C  -0.28544 -3.08332  2.16067
H  -1.36778 -3.14207  2.10540
C   0.39367 -3.93205  3.05428
H  -0.18284 -4.63857  3.65477
C   1.78891 -3.87958  3.20659
H   2.28850 -4.53653  3.91901
C   2.53537 -2.97271  2.43545
H   3.61822 -2.89468  2.54340
C   1.84653 -2.16401  1.52925
C   3.18198 -1.66705 -1.05025
C   3.48710 -3.03894 -1.01397
H   3.30135 -3.61032 -0.10666
C   3.99351 -3.67716 -2.15560
H   4.21111 -4.74473 -2.12166
C   4.20711 -2.95281 -3.33507
H   4.59250 -3.45384 -4.22294
C   3.91927 -1.57952 -3.37115
H   4.08225 -1.00796 -4.28505
C   3.40491 -0.94111 -2.23783
H   3.17370  0.12131 -2.27096
C   3.69593  0.17037  1.16688
C   3.48566  0.68283  2.46327
H   2.56028  0.46472  2.98840
C   4.46376  1.47614  3.07186
H   4.28641  1.86728  4.07399
C   5.65892  1.76762  2.39847
H   6.41992  2.38669  2.87435
C   5.87049  1.25840  1.11104
H   6.79513  1.48173  0.57840
C   4.89493  0.46425  0.49498
H   5.06961  0.07735 -0.50638
C   0.12760  0.57806  2.12944
C   0.14632  0.17792  3.48157
H   0.11374 -0.88148  3.73908
C   0.18844  1.14324  4.50436
H   0.19248  0.82015  5.54770
C   0.21438  2.51984  4.21269
H   0.23074  3.25013  5.02226
C   0.22586  2.94902  2.87540
H   0.24430  4.01155  2.62773
C   0.20224  1.97173  1.87185
C  -0.89211  3.33885 -0.55342
C  -2.00398  3.69599  0.23123
H  -2.12940  3.26896  1.22283
C  -2.96085  4.58759 -0.27039
H  -3.81941  4.84711  0.34773
C  -2.82381  5.12522 -1.55646
H  -3.57526  5.81157 -1.94700
C  -1.71092  4.78125 -2.33823
H  -1.58957  5.20357 -3.33622
C  -0.74819  3.89603 -1.84015
H   0.11318  3.63848 -2.45318
C   1.91865  2.68611 -0.45623
C   2.37434  2.50289 -1.77539
H   1.75768  1.96649 -2.49593
C   3.62446  2.99756 -2.16965
H   3.96396  2.84842 -3.19514
C   4.43596  3.66941 -1.24595
H   5.41447  4.04339 -1.54724
C   3.99086  3.84419  0.07091
H   4.62688  4.34189  0.80170
C   2.73878  3.35754  0.46596
H   2.41378  3.47075  1.49700
C  -3.35592  0.74599  1.13659
C  -3.00013  1.01107  2.47285
H  -2.15171  0.50100  2.91513
C  -3.71399  1.94783  3.23026
H  -3.40579  2.15050  4.25585
C  -4.80319  2.62655  2.66571
H  -5.35498  3.36304  3.25047
C  -5.18157  2.34679  1.34569
H  -6.03284  2.86158  0.89915
C  -4.46367  1.41475  0.58477
```

```
H  -4.76110  1.22239 -0.44287
C  -3.10578 -0.21608 -1.54002
C  -2.71682  0.93153 -2.25977
H  -2.01238  1.63110 -1.82196
C  -3.25660  1.20778 -3.51997
H  -2.93891  2.10570 -4.04899
C  -4.19013  0.33153 -4.09126
H  -4.60222  0.53460 -5.07974
C  -4.59853 -0.80150 -3.37576
H  -5.33693 -1.48097 -3.80262
C  -4.07278 -1.06767 -2.10216
H  -4.42502 -1.93547 -1.55021
C  -3.11312 -2.08961  0.67990
C  -2.88382 -3.24845 -0.08763
H  -2.35718 -3.17871 -1.03801
C  -3.32078 -4.50195  0.35249
H  -3.11916 -5.38357 -0.25545
C  -3.99956 -4.61822  1.57398
H  -4.33121 -5.59477  1.92656
C  -4.25218 -3.46955  2.33507
H  -4.78805 -3.54736  3.28143
C  -3.81725 -2.21238  1.89030
H  -4.01626 -1.33065  2.49505
```

2_m06

```
M06 energy =       -2730.74326942
Enthalpy 0K =      -2729.871249
Enthalpy 298K =    -2729.810160
Free energy 298K =       -2729.967270
Low Freq. = 18.8759 cm-1

Ru  0.02971 -0.35714  0.20351
Zn  0.04757 -0.24433 -2.31617
Zn  0.20175 -2.53220 -1.07733
P   2.38392 -0.78364  0.37894
P   0.22637  2.06344  0.01152
P  -2.36183 -0.52588  0.13817
C   0.14772  0.48630 -4.15193
H  -0.14964  1.54478 -4.17436
H  -0.54000 -0.06457 -4.80570
H   1.15853  0.40338 -4.57376
C   0.53231 -4.38769 -1.61903
H   1.42796 -4.44325 -2.25014
H  -0.31205 -4.80431 -2.18171
H   0.69477 -5.00348 -0.72445
C   0.46988 -2.13293  1.32871
C  -0.23059 -3.09117  2.07822
H  -1.30967 -3.19782  1.97671
C   0.44086 -3.91886  2.98102
H  -0.12784 -4.65112  3.55269
C   1.81799 -3.82111  3.17906
H   2.31383 -4.46539  3.90176
C   2.55198 -2.89475  2.44042
H   3.62816 -2.78597  2.58165
C   1.87258 -2.10452  1.52170
C   3.25073 -1.63555 -1.01686
C   3.82349 -2.89753 -0.85006
H   3.78610 -3.38515  0.12252
C   4.40277 -3.55441 -1.93279
H   4.83184 -4.54420 -1.79477
C   4.41756 -2.95617 -3.18807
H   4.85950 -3.47649 -4.03464
C   3.86104 -1.69064 -3.36066
H   3.86798 -1.21576 -4.33939
C   3.28219 -1.03528 -2.28033
H   2.84517 -0.04574 -2.41928
C   3.72414  0.22910  1.14752
C   3.56938  0.68977  2.46077
H   2.68371  0.41703  3.03148
C   4.53828  1.50288  3.03689
H   4.40246  1.85359  4.05791
C   5.67122  1.86501  2.31270
H   6.43009  2.49923  2.76613
C   5.82939  1.41175  1.00747
H   6.70948  1.69304  0.43270
C   4.86099  0.59954  0.42569
H   4.99384  0.25643 -0.59890
C   0.15952  0.64221  2.08146
C   0.21550  0.25220  3.42960
H   0.19494 -0.80754  3.69564
C   0.28758  1.20811  4.44196
H   0.32382  0.88504  5.48238
C   0.30821  2.57628  4.15347
H   0.35001  3.30433  4.96083
C   0.27483  2.99819  2.82773
H   0.28247  4.05998  2.57628
C   0.22181  2.02529  1.82977
C  -0.97938  3.36237 -0.52993
C  -2.10493  3.62335  0.25662
H  -2.21697  3.13898  1.22689
C  -3.09315  4.49070 -0.19920
H  -3.96446  4.68130  0.42451
C  -2.97088  5.10396 -1.44156
H  -3.74621  5.77913 -1.79646
C  -1.84813  4.85526 -2.22669
H  -1.73991  5.33872 -3.19534
C  -0.85813  3.99027 -1.77439
H   0.01869  3.80710 -2.39453
C   1.83842  2.77785 -0.54980
C   2.26258  2.56971 -1.86676
H   1.64136  1.99221 -2.55490
C   3.47583  3.08745 -2.30983
H   3.79088  2.91856 -3.33762
C   4.28312  3.81094 -1.43710
H   5.23626  4.20887 -1.77849
C   3.87221  4.01375 -0.12355
H   4.50750  4.56166  0.56912
C   2.65568  3.50313  0.31881
H   2.35356  3.64676  1.35452
C  -3.34565  0.48055  1.35898
C  -2.97068  0.42133  2.70609
H  -2.15933 -0.23772  3.00643
C  -3.60612  1.20830  3.65913
H  -3.28223  1.15968  4.69676
C  -4.63902  2.06183  3.27944
H  -5.13529  2.68345  4.02145
C  -5.03499  2.11263  1.94727
H  -5.84726  2.77030  1.64338
C  -4.39079  1.33145  0.99036
H  -4.69933  1.40412 -0.05106
C  -3.26319 -0.07782 -1.43275
C  -2.78587  0.97192 -2.21948
H  -1.86961  1.48571 -1.93815
C  -3.47818  1.39600 -3.34955
H  -3.08005  2.21781 -3.94179
C  -4.66535  0.76891 -3.71081
```

```
H  -5.20809  1.09173 -4.59637
C  -5.15834 -0.27311 -2.93022
H  -6.09013 -0.76445 -3.20193
C  -4.46726 -0.69020 -1.79698
H  -4.87583 -1.49354 -1.18653
C  -3.05768 -2.23310  0.41450
C  -2.76976 -3.22660 -0.52828
H  -2.23447 -2.96979 -1.44381
C  -3.17271 -4.54098 -0.32895
H  -2.92587 -5.29718 -1.07073
C  -3.88895 -4.88242  0.81566
H  -4.20062 -5.91146  0.97860
C  -4.21428 -3.89746  1.74061
H  -4.79047 -4.15034  2.62799
C  -3.80490 -2.57994  1.54081
H  -4.06686 -1.82273  2.27638
```

2_tpss

```
TPSS energy =      -2732.64968804
Enthalpy 0K =      -2731.787454
Enthalpy 298K =    -2731.724428
Free energy 298K =       -2731.889586
Low Freq. = 13.0500 cm-1

Ru  0.02287 -0.34969  0.23467
Zn  0.07066 -0.27844 -2.27607
Zn  0.14182 -2.54453 -1.00256
P   2.38627 -0.81009  0.38684
P   0.23027  2.08006  0.03057
P  -2.36130 -0.51508  0.11499
C   0.23353  0.24130 -4.19301
H  -0.02143  1.29710 -4.36944
H  -0.45975 -0.37013 -4.78931
H   1.25015  0.06460 -4.57410
C   0.31621 -4.37811 -1.72351
H   1.31011 -4.51395 -2.17100
H  -0.43434 -4.56427 -2.50595
H   0.18545 -5.12906 -0.93219
C   0.44646 -2.13451  1.36510
C  -0.26894 -3.07614  2.14350
H  -1.35149 -3.14488  2.07423
C   0.40393 -3.93023  3.03634
H  -0.17445 -4.64345  3.62450
C   1.79696 -3.87383  3.20135
H   2.29214 -4.53315  3.91291
C   2.54676 -2.96112  2.44213
H   3.62671 -2.88342  2.56757
C   1.86583 -2.14503  1.53103
C   3.27170 -1.65400 -1.02493
C   3.80274 -2.95064 -0.88667
H   3.69715 -3.48146  0.05695
C   4.44437 -3.57152 -1.96792
H   4.84010 -4.57929 -1.85114
C   4.57204 -2.90338 -3.19271
H   5.06826 -3.38920 -4.03127
C   4.05337 -1.60851 -3.33677
H   4.14627 -1.08326 -4.28608
C   3.40196 -0.98999 -2.26197
H   2.99793  0.01417 -2.38080
C   3.77148  0.14813  1.19559
C   3.61761  0.58567  2.52756
H   2.70279  0.36038  3.07004
C   4.63884  1.30917  3.15563
H   4.50617  1.63604  4.18603
C   5.82187  1.60963  2.46599
H   6.61592  2.16971  2.95799
C   5.97874  1.18094  1.14180
H   6.89527  1.40569  0.59795
C   4.96094  0.45541  0.50803
H   5.10016  0.12374 -0.51842
C   0.15872  0.65758  2.11704
C   0.19210  0.26650  3.47719
H   0.13935 -0.78970  3.74475
C   0.27999  1.23045  4.49703
H   0.29780  0.90817  5.53946
C   0.33514  2.60579  4.20418
H   0.38824  3.33547  5.01129
C   0.32132  3.03169  2.86777
H   0.35073  4.09376  2.62266
C   0.25250  2.05209  1.86333
C  -0.96337  3.44076 -0.47257
C  -2.05556  3.74602  0.36380
H  -2.18027  3.22022  1.30767
C  -2.98115  4.72996 -0.00930
H  -3.81641  4.95415  0.65210
C  -2.83501  5.41723 -1.22123
H  -3.55510  6.18195 -1.50845
C  -1.75171  5.11941 -2.05930
H  -1.62257  5.65481 -2.99911
C  -0.82146  4.13950 -1.68885
H   0.02549  3.93487 -2.34099
C   1.83881  2.81197 -0.57571
C   2.22523  2.61516 -1.91688
H   1.60358  2.01091 -2.57982
C   3.40394  3.19158 -2.41133
H   3.68423  3.03772 -3.45260
C   4.21865  3.95705 -1.56623
H   5.13748  4.40027 -1.94711
C   3.85041  4.14119 -0.22686
H   4.48688  4.72091  0.43977
C   2.66656  3.57633  0.26656
H   2.39702  3.71331  1.31114
C  -3.45925  0.57348  1.19722
C  -3.10802  0.75597  2.55010
H  -2.20417  0.29414  2.93712
C  -3.90079  1.54421  3.39621
H  -3.60618  1.67616  4.43626
C  -5.05615  2.16547  2.90263
H  -5.67009  2.78237  3.55747
C  -5.41642  1.98618  1.56022
H  -6.31443  2.46100  1.16671
C  -4.62654  1.19623  0.71313
H  -4.92195  1.07068 -0.32554
C  -3.16652 -0.18466 -1.55891
C  -2.72254  0.92385 -2.30713
H  -1.92884  1.55540 -1.91324
C  -3.31324  1.24994 -3.53529
H  -2.95369  2.11564 -4.08847
C  -4.35431  0.46331 -4.04511
H  -4.80699  0.70669 -5.00503
C  -4.81323 -0.63502 -3.30647
H  -5.62925 -1.24782 -3.68729
C  -4.23390 -0.95119 -2.06879
H  -4.62038 -1.79435 -1.50182
C  -3.10348 -2.20079  0.50519
C  -2.84252 -3.28134 -0.36268
```

```
H  -2.28908 -3.11643 -1.28760
C  -3.30696 -4.57014 -0.07160
H  -3.08810 -5.38636 -0.75779
C  -4.04596 -4.80245  1.09664
H  -4.40420 -5.80415  1.32852
C  -4.32712 -3.73465  1.95831
H  -4.91096 -3.90070  2.86267
C  -3.86385 -2.44298  1.66477
H  -4.09503 -1.62594  2.34397
```

2_wb97xd

```
wB97XD energy =   -2731.80737552
Enthalpy 0K =     -2730.919032
Enthalpy 298K =   -2730.859030
Free energy 298K =      -2731.014221
Low Freq. = 17.1393 cm-1

Ru  0.03390 -0.37533  0.21222
Zn  0.09112 -0.21779 -2.28367
Zn  0.08409 -2.53085 -1.06871
P   2.36245 -0.82301  0.35871
P   0.25558  2.01198  0.06243
P  -2.35262 -0.47999  0.14937
C   0.25976  0.54376 -4.09813
H  -0.06504  1.59231 -4.12645
H  -0.37612 -0.00985 -4.79915
H   1.28882  0.49780 -4.47609
C   0.18661 -4.38044 -1.71221
H   1.10509 -4.53500 -2.28951
H  -0.66096 -4.63333 -2.36109
H   0.19052 -5.08217 -0.87039
C   0.47435 -2.17261  1.32563
C  -0.20893 -3.12471  2.09849
H  -1.28418 -3.23044  2.02647
C   0.47839 -3.94236  2.99882
H  -0.08053 -4.66530  3.58745
C   1.85864 -3.84204  3.17208
H   2.36728 -4.47688  3.89106
C   2.57768 -2.92141  2.41219
H   3.65236 -2.81023  2.53185
C   1.87890 -2.14170  1.49989
C   3.19115 -1.65080 -1.06507
C   3.56185 -2.99551 -1.00810
H   3.41475 -3.55987 -0.09327
C   4.08639 -3.62836 -2.13389
H   4.35566 -4.67844 -2.08035
C   4.25183 -2.92431 -3.32133
H   4.65232 -3.42195 -4.19884
C   3.89635 -1.57768 -3.38291
H   4.01960 -1.02045 -4.30624
C   3.36579 -0.94804 -2.26385
H   3.08372  0.09878 -2.32146
C   3.69634  0.19233  1.11864
C   3.52027  0.66496  2.42508
H   2.62496  0.40795  2.97913
C   4.48741  1.46809  3.01810
H   4.33362  1.82701  4.03103
C   5.64262  1.80787  2.31837
H   6.39839  2.43401  2.78296
C   5.82485  1.33822  1.02210
H   6.72119  1.59839  0.46772
C   4.85747  0.53616  0.42327
H   5.01599  0.18083 -0.58889
C   0.15324  0.58532  2.09653
C   0.18607  0.16875  3.43787
H   0.17418 -0.89136  3.68030
C   0.21535  1.10806  4.46873
H   0.23029  0.76800  5.50162
C   0.21505  2.48212  4.20481
H   0.22206  3.19427  5.02436
C   0.21343  2.92920  2.88655
H   0.21302  3.99241  2.65846
C   0.20180  1.97381  1.87208
C  -0.92077  3.30027 -0.54075
C  -2.01386  3.66971  0.24662
H  -2.12601  3.26719  1.24831
C  -2.97659  4.54333 -0.25277
H  -3.82241  4.81206  0.37174
C  -2.86210  5.05350 -1.54107
H  -3.61769  5.72877 -1.93033
C  -1.76881  4.69883 -2.32879
H  -1.66480  5.09980 -3.33235
C  -0.80333  3.83114 -1.83137
H   0.04355  3.56682 -2.45638
C   1.88280  2.72015 -0.44186
C   2.34288  2.53487 -1.74947
H   1.74768  1.97212 -2.46423
C   3.56493  3.06434 -2.15378
H   3.90508  2.91228 -3.17369
C   4.34657  3.77852 -1.25078
H   5.30421  4.18495 -1.56081
C   3.90111  3.95720  0.05516
H   4.51511  4.49241  0.77208
C   2.67723  3.43241  0.45858
H   2.35530  3.55428  1.48669
C  -3.36126  0.70024  1.17460
C  -2.99030  0.92424  2.50512
H  -2.14421  0.39615  2.92407
C  -3.67967  1.83685  3.29676
H  -3.35620  2.00426  4.31922
C  -4.76325  2.53644  2.77187
H  -5.29890  3.25576  3.38393
C  -5.15941  2.30083  1.45889
H  -6.00899  2.83279  1.04145
C  -4.46446  1.39186  0.66541
H  -4.78129  1.24072 -0.35985
C  -3.16716 -0.21328 -1.50253
C  -2.74899  0.88161 -2.26547
H  -1.97095  1.53518 -1.89230
C  -3.33919  1.17633 -3.48870
H  -2.99002  2.03547 -4.05252
C  -4.36357  0.37086 -3.97752
H  -4.82139  0.58842 -4.93738
C  -4.80310 -0.71105 -3.22099
H  -5.61084 -1.33816 -3.58562
C  -4.21880 -0.99563 -1.98824
H  -4.59570 -1.82923 -1.40698
C  -3.07535 -2.11139  0.65434
C  -2.85696 -3.22849 -0.16090
H  -2.38189 -3.11364 -1.13089
C  -3.24919 -4.49895  0.24142
H  -3.05265 -5.34807 -0.40504
C  -3.87846 -4.67481  1.47179
H  -4.17809 -5.66659  1.79530
C  -4.12726 -3.56949  2.27834
H  -4.62859 -3.69348  3.23321
```

```
C  -3.73222 -2.29566  1.87210
H  -3.93088 -1.44876  2.51902
```

S-4 References

(1) O'Leary, N.; Miloserdov, F. M.; Mahon, M. F.; Whittlesey, M. K. Transforming PPh_3 into bidentate phosphine ligands at Ru-Zn heterobimetallic complexes. *Dalton Trans.* **2019,** *48*, 14000-14009.

(2) Specklin, D.; Fliedel, C.; Gourlaouen, C.; Bruyere, J. C.; Avilés, T.; Boudon, C.; Ruhlmann, L.; Dagorne, S. N-Heterocyclic carbene based tri-organyl-Zn-alkyl cations: Synthesis, structures, and use in CO_2 functionalization. *Chem. Eur. J.* **2017,** *23*, 5509-5519.

(3) Rit, A. S., T. P.; Maron, L.; Okuda, J. Mixed alkyl hydrido complexes of zinc: Synthesis, structure, and reactivity. *Organometallics* **2014,** *33*, 2039-2047.

(4) Frisch, M. J.; Trucks, G. W.; Schlegel, H. B.; Scuseria, G. E.; Robb, M. A.; Cheeseman, J. R.; Scalmani, G.; Barone, V.; Petersson, G. A.; Nakatsuji, H.; Li, X.; Caricato, M.; Marenich, A. V.; Bloino, J.; Janesko, B. G.; Gomperts, R.; Mennucci, B.; Hratchian, H. P.; Ortiz, J. V.; Izmaylov, A. F.; Sonnenberg, J. L.; Williams-Young, D.; Ding, F.; Lipparini, F.; Egidi, F.; Goings, J.; Peng, B.; Petrone, A.; Henderson, T.; Ranasinghe, D.; Zakrzewski, V. G.; Gao, J.; Rega, N.; Zheng, G.; Liang, W.; Hada, M.; Ehara, M.; Toyota, K.; Fukuda, R.; Hasegawa, J.; Ishida, M.; Nakajima, T.; Honda, Y.; Kitao, O.; Nakai, H.; Vreven, T.; Throssell, K.; Montgomery, J. A., Jr.; Peralta, J. E.; Ogliaro, F.; Bearpark, M. J.; Heyd, J. J.; Brothers, E. N.; Kudin, K. N.; Staroverov, V. N.; Keith, T. A.; Kobayashi, R.; Normand, J.; Raghavachari, K.; Rendell, A. P.; Burant, J. C.; Iyengar, S. S.; Tomasi, J.; Cossi, M.; Millam, J. M.; Klene, M.; Adamo, C.; Cammi, R.; Ochterski, J. W.; Martin, R. L.; Morokuma, K.; Farkas, O.; Foresman, J. B.; Fox, D. J. *Gaussian16, Revision C.01*, Gaussian, Inc.: Wallingford CT, 2016.

(5) Andrae, D.; Häußermann, U.; Dolg, M.; Stoll, H.; Preuß, H. Energy-adjusted ab initio pseudopotentials for the second and third row transition elements. *Theor. Chim. Acta* **1990**, *77*, 123-141.

(6) Hehre, W. J.; Ditchfield, R.; Pople, J. A. Self-consistent molecular orbital methods. XII. Further extensions of Gaussian-type basis sets for use in molecular orbital studies of organic molecules. *J. Chem. Phys.* **1972**, *56*, 2257-2261.

(7) Hariharan, P. C.; Pople, J. A. The influence of polarization functions on molecular orbital hydrogenation energies. *Theor. Chim. Acta* **1973**, *28*, 213-222.

(8) Höllwarth, A.; Böhme, M.; Dapprich, S.; Ehlers, A. W.; Gobbi, A.; Jonas, V.; Köhler, K. F.; Stegmann, R.; Veldkamp, A.; Frenking, G. A set of d-polarization functions for pseudo-potential basis sets of the main group elements Al-Bi and f-type polarization functions for Zn, Cd, Hg. *Chem. Phys. Lett.* **1993**, *208*, 237-240.

(9) Becke, A. D. Density-functional exchange-energy approximation with correct asymptotic behavior. *Phys. Rev. A* **1988**, *38*, 3098-3100.

(10) Perdew, J. P., Density-functional approximation for the correlation energy of the inhomogeneous electron gas. *Phys. Rev. B* **1986**, *33*, 8822-8824.

(11) Bader, R. F. W. *Atoms in Molecules: A Quantum Theory*. Clarendon Press: 1994.

(12) Keith, T. A. *AIMAll (Version 17.11.14)*, TK Gristmill Software: Overland Park KS, USA, 2017.

(13) Contreras-García, J.; Johnson, E. R.; Keinan, S.; Chaudret, R.; Piquemal, J.-P.; Beratan, D. N.; Yang, W. NCIPLOT: A Program for Plotting Noncovalent Interaction Regions. *J. Chem. Theory Comp.* **2011**, *7*, 625-632.

(14) Humphrey, W.; Dalke, A.; Schulten, K. VMD: Visual molecular dynamics. *J. Mol. Graph.* **1996**, *14*, 33-38.

(15) Mitoraj, M. P.; Parafiniuk, M.; Srebro, M.; Handzlik, M.; Buczek, A.; Michalak, A. Applications of the ETS-NOCV method in descriptions of chemical reactions. *J. Mol. Model.* **2011**, *17*, 2337.

(16) te Velde, G.; Bickelhaupt, F. M.; Baerends, E. J.; Guerra, C. F.; van Gisbergen, S. J. A.; Snijders, J. G.; Ziegler, T. Chemistry with ADF. *J. Comp. Chem.* **2001**, *22*, 931-967.

(17) www.chemcraftprog.com.

(18) Perdew, J. P.; Burke, K.; Ernzerhof, M. Generalized gradient approximation made simple. *Phys. Rev. Lett.* **1996**, *77*, 3865-3868.

(19) Lee, C.; Yang, W.; Parr, R. G. Development of the Colle-Salvetti correlation-energy formula into a functional of the electron density. *Phys. Rev. B* **1988**, *37*, 785-789.

(20) Grimme, S. Semiempirical GGA-type density functional constructed with a long-range dispersion correction. *J. Comp. Chem.* **2006**, *27*, 1787-1799.

(21) Tao, J.; Perdew, J. P.; Staroverov, V. N.; Scuseria, G. E. Climbing the density functional ladder: Nonempirical meta-generalized gradient approximation designed for molecules and solids. *Phys. Rev. Lett.* **2003**, *91*, 146401.

(22) Adamo, C.; Barone, V. Toward reliable density functional methods without adjustable parameters: The PBE0 model. *J. Chem. Phys.* **1999**, *110*, 6158-6170.

(23) Becke, A. D., Density-functional thermochemistry. III. The role of exact exchange. *J. Chem. Phys.* **1993**, *98*, 5648-5652.

(24) Chai, J.-D.; Head-Gordon, M. Long-range corrected hybrid density functionals with damped atom–atom dispersion corrections. *Phys. Chem. Chem. Phys.* **2008**, *10*, 6615-6620.

(25) Zhao, Y.; Truhlar, D. G., The M06 suite of density functionals for main group thermochemistry, thermochemical kinetics, noncovalent interactions, excited states, and transition elements: two new functionals and systematic testing of four M06-class functionals and 12 other functionals. *Theor. Chem. Acc.* **2008**, *120*, 215-241.

(26) Riddlestone, I. M.; Rajabi, N. A.; Lowe, J. P.; Mahon, M. F.; Macgregor, S. A.; Whittlesey, M. K. Activation of H_2 over the Ru-Zn bond in the transition metal-Lewis acid heterobimetallic species $[Ru(IPr)_2(CO)ZnEt]^+$. *J. Am. Chem. Soc.* **2016**, *138*, 11081-11084.
